# K29-Selective Ubiquitin Binding Domain Reveals Structural Basis of Specificity and Heterotypic Nature of K29 Polyubiquitin

**DOI:** 10.1016/j.molcel.2015.01.041

**Published:** 2015-04-02

**Authors:** Yosua Adi Kristariyanto, Syed Arif Abdul Rehman, David G. Campbell, Nicholas A. Morrice, Clare Johnson, Rachel Toth, Yogesh Kulathu

**Affiliations:** 1 MRC Protein Phosphorylation and Ubiquitylation Unit, College of Life Sciences, University of Dundee, Dow Street, Dundee DD1 5EH, UK

## Abstract

Polyubiquitin chains regulate diverse cellular processes through the ability of ubiquitin to form chains of eight different linkage types. Although detected in yeast and mammals, little is known about K29-linked polyubiquitin. Here we report the generation of K29 chains in vitro using a ubiquitin chain-editing complex consisting of the HECT E3 ligase UBE3C and the deubiquitinase vOTU. We determined the crystal structure of K29-linked diubiquitin, which adopts an extended conformation with the hydrophobic patches on both ubiquitin moieties exposed and available for binding. Indeed, the crystal structure of the NZF1 domain of TRABID in complex with K29 chains reveals a binding mode that involves the hydrophobic patch on only one of the ubiquitin moieties and exploits the flexibility of K29 chains to achieve linkage selective binding. Further, we establish methods to study K29-linked polyubiquitin and find that K29 linkages exist in cells within mixed or branched chains containing other linkages.

## Highlights

• Large-scale enzymatic assembly and purification of K29-linked polyubiquitin chains
• K29 diubiquitin adopts extended conformation in crystal structure
• Crystal structure of K29 diubiquitin in complex with selective binding domain
• Presence of K29 chains within mixed/branched chains containing other linkages

## Graphical Abstract

**Figure:**
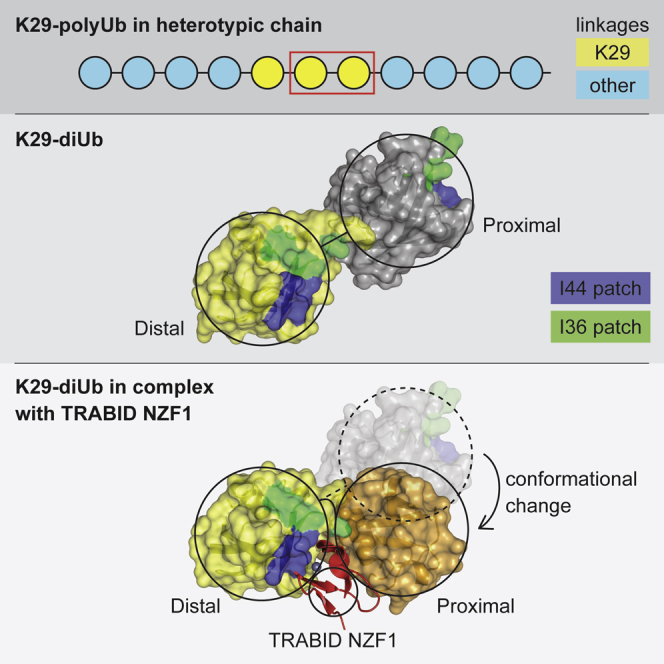

## Teaser

Kristariyanto et al. find that K29-linked ubiquitin chains are present within ubiquitin chains containing other linkage types. They describe a method to assemble K29 chains, and they characterize a protein domain that selectively binds to these chains.

## Introduction

Protein ubiquitylation is a reversible posttranslational modification that regulates the activity, function, and fate of modified proteins and is fundamental to diverse biological processes (Komander and Rape, 2012). Topologically distinct ubiquitin (Ub) signals are produced when proteins are monoubiquitylated at one or multiple sites or polyubiquitylated by modification with Ub polymers (Haglund and Dikic, 2005). Homotypic Ub polymers of eight different linkage types can be assembled in which the C terminus of a distal Ub is attached to one of the seven lysine residues (K6, K11, K27, K29, K33, K48, and K63) or to the N-terminal M1 of the proximal Ub. In addition to homotypic chains, heterotypic polyUb of complex topology involving mixed (alternating linkages) or branched (single Ub within a chain modified at two or more sites) types can be formed and have roles in endocytosis, immune signaling, and protein degradation (Boname et al., 2010; Emmerich et al., 2013; Meyer and Rape, 2014).

Differently linked polyUb chains act as functionally distinct signals, and this is mediated in part by Ub binding domains (UBDs) that selectively bind and decode different polyUb modifications (Hurley et al., 2006; Husnjak and Dikic, 2012). UBDs and enzymes of the Ub system exploit the unique conformational and dynamic properties of differently linked polyUb types to achieve linkage selectivity. Structural analyses of five linkage types (K6, K11, K48, K63, and M1) reveal that Ub chains adopt distinct conformations that can be broadly classified as “open” or “compact.” In the compact conformation adopted by K6-, K11-, and K48-linked diUb, an intermolecular interface is present between the distal and proximal Ub, whereas in the extended conformation of M1- and K63-linked polyUb, the linkage is the only point of contact between the two Ub molecules (Datta et al., 2009; Komander et al., 2009). However, polyUb is flexible, as compact conformations have been observed for K63 and M1 linkages (Datta et al., 2009; Rohaim et al., 2012).

Proteomic analyses have detected all eight Ub linkages in cells and their relative abundance varies between different cell types and organisms (Dammer et al., 2011; Peng et al., 2003; Ziv et al., 2011). The abundance of particular linkage types increases in response to a specific stimulus as has been observed for M1-, K11-, and K63-linked polyUb (Behrends and Harper, 2011). K48-linked Ub chains, the most abundant linkage type detected in resting cells, function as a signal for proteasomal degradation, while K63- and M1-linked chains have non-proteolytic roles in DNA damage response and immune signaling pathways (Behrends and Harper, 2011; Chau et al., 1989; Spence et al., 1995; Wang et al., 2001). In contrast, the cellular roles of polyUb linked via K6, K27, K29, and K33 (also referred to as atypical polyUb), have yet to be elucidated (Kulathu and Komander, 2012).

Of the atypical linkages, K29 polyUb is the most abundant in resting mammalian cells (Dammer et al., 2011). While the cellular function of this linkage type is unclear, the amount of K29 polyUb increases following proteasomal inhibition. In yeast, K29-linked polyUb has been linked to the Ub-fusion-degradation pathway, where it mediates substrate turnover (Johnson et al., 1995; Koegl et al., 1999). Several HECT E3 ligases, including ITCH, UBR5, and UBE3C, have been suggested to make K29 linkages in cells (Chastagner et al., 2006; Hay-Koren et al., 2011; You and Pickart, 2001). Recently, the deubiquitinase (DUB) TRABID was identified as having a preference for hydrolyzing K29 and K33 linkages (Licchesi et al., 2012; Virdee et al., 2010). TRABID was discovered as a positive regulator of Wnt signaling and has three tandem NPL4 zinc fingers (NZFs) that are thought to have a preference for binding to K63- and M1-linked polyUb (Komander et al., 2009; Tran et al., 2008).

Research into understanding roles for atypical chains is hampered by the lack of tools and the inability to assemble polyUb chains of different lengths on a large-scale. Although chemical biology approaches can be used to make diUb of all linkage types (Kumar et al., 2010; El Oualid et al., 2010; Virdee et al., 2010), there are no known methods for the large-scale assembly of K27, K29, and K33 linkages using enzymatic means. Further, UBDs that bind specifically to polyUb of a particular linkage type are very useful as polyUb sensors to probe the cellular roles of that linkage (van Wijk et al., 2012). However, no such linkage-selective UBDs have been characterized for atypical chains.

Here we establish a Ub chain-editing complex consisting of an E3 ligase and DUB combination for the large-scale assembly of K29-linked polyUb. This enabled us to determine the structure of K29-linked diUb, which reveals an open conformation. Furthermore, we identify and structurally characterize the first NZF domain (NZF1) of the DUB TRABID as being a highly selective binder of K29- and K33-linked polyUb. Finally, using these selective NZFs, we demonstrate that K29-linked chains feature in mixed or branched chains in cells. Interestingly, short K29-linked chains (diUb to tetraUb) are present in long complex polyUb mixtures suggesting that these heterotypic chains may have distinct properties with specialized function.

## Results

### Assembly of K29-Linked PolyUb

PolyUb of several linkage types, including K11, K48, and K63, can be assembled in vitro by E2 enzymes (Bremm et al., 2010; Pickart and Raasi, 2005; Ye and Rape, 2009). In addition, HECT-family ligases have been used to catalyze assembly of K6-linked polyUb (Hospenthal et al., 2013). UBE3C, a relatively uncharacterized HECT E3 ligase, has been reported to mainly assemble K29 and K48 linkages (You and Pickart, 2001). In vitro, UBE3C generates small amounts of free diUb and triUb that is rapidly converted to high-molecular weight polyUb, and is mostly autoubiquitylated UBE3C (Figure S1A). Autoubiquitylation of UBE3C occurs in *trans*, as the catalytic dead UBE3C C1051A can be ubiquitylated by wild-type UBE3C (Figure S1C).

**Figure 1:**
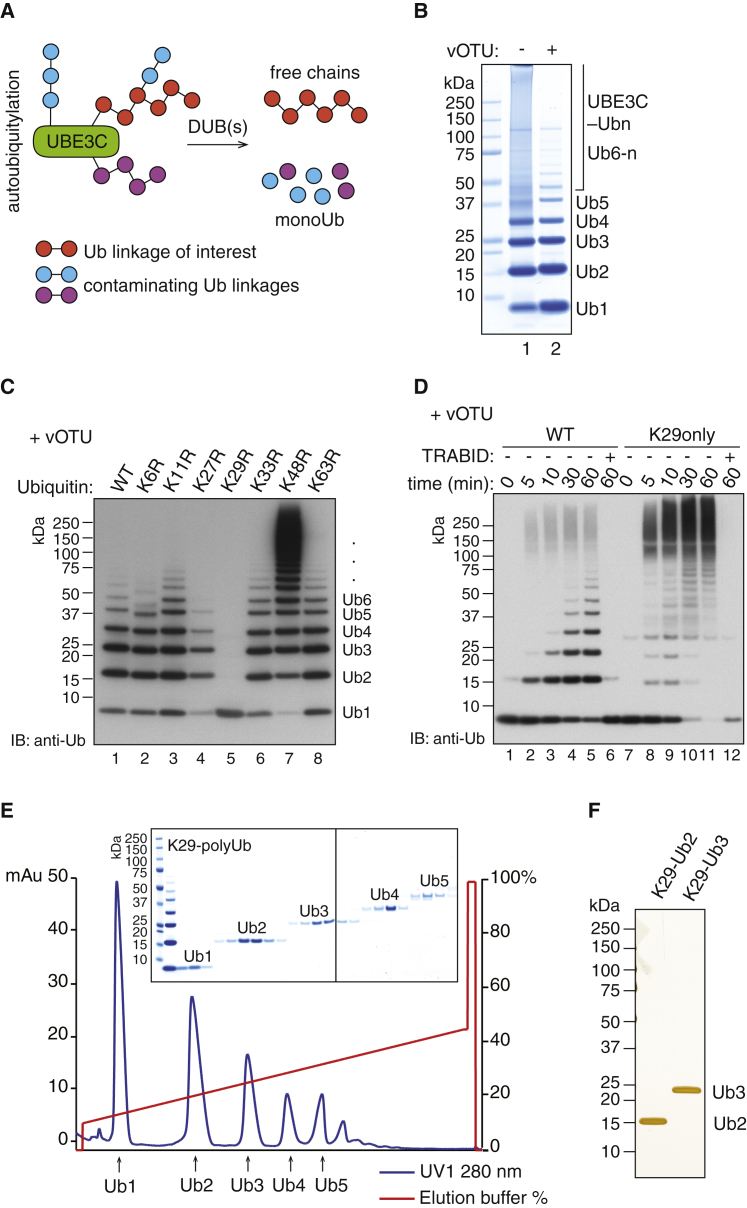
Assembly and Purification of K29-Linked PolyUb (A) A schematic illustrates how E3 ligase and DUB(s) are used to assemble free polyUb chains. (B) Large-scale assembly of polyUb chains by UBE3C in the presence of UBE1, UBE2D3, and Ub. The addition of vOTU releases free polyUb chains. (C) Ubiquitylation assays of UBE3C in the presence of UBE1, UBE2D3, and wild-type Ub or K-to-R Ub mutants. vOTU was added at the end of the ubiquitylation reaction. (D) Time course of ubiquitylation by UBE3C in the presence of UBE1, UBE2D3, vOTU, and wild-type Ub or K29only Ub mutant. To one half of the reaction stopped at 60 min, apyrase was added to quench ATP followed by DUB hydrolysis with TRABID. (E) Purification of K29-linked diUb, triUb, tetraUb, and pentaUb by cation-exchange chromatography. Proteins from the corresponding peak fractions were visualized on Coomassie-stained SDS-PAGE gel. (F) The K29-linked diUb and triUb purified in (E) are visualized in silver-stained SDS-PAGE gel. See also Figure S1.

In order to obtain free K29 polyUb chains, a DUB that catalyzes removal of contaminating linkages and releases free chains from autoubiquitylated UBE3C is required (Figure 1A). The viral DUB vOTU has recently been shown to catalyze cleavage of all linkages except M1, K27, and K29 (Ritorto et al., 2014). Thus we hypothesized that vOTU may release free polyUb chains from autoubiquitylated UBE3C, thereby enhancing the yield of free chains. Indeed, inclusion of vOTU in the assembly reactions results in unanchored polyUb chains (Figure 1B). We then performed a linkage type analysis by using Ub mutants containing Lys to Arg substitutions. Free polyUb chains that were assembled in the presence of vOTU were not impaired with K6R, K11R, K33R, K48R, or K63R mutants (Figure 1C). By contrast, formation of free polyUb chains was significantly reduced in the presence of Ub K29R, demonstrating that the enzymes UBE3C, UBE2D3, and vOTU can together function as a Ub chain-editing complex for the specific assembly of K29 linkages (Figure 1C). In order to confirm the linkage type of the chains formed, we incubated the chains assembled by this complex with the DUB TRABID that has known specificity for hydrolyzing K29 and K33 linkages. Indeed, chains treated with TRABID were reduced to monoUb, while the M1-specific DUB OTULIN did not hydrolyze these chains (Figure S1B). When the K29only mutant of Ub, where all Lys residues but K29 are mutated to Arg, was used in the assembly reaction, long polyUb chains are assembled and these were hydrolyzed down to monoUb by TRABID (Figure 1D). Using parallel reaction monitoring (pRM) liquid chromatography tandem mass spectrometry (LC-MS/MS) analysis of tryptic fragments (Peterson et al., 2012), we further verified that the Ub chains produced using the described method only contained K29 linkages, and other linkages were not detected (Figure S1D). By scaling up the assembly reactions using the chain-editing complex, we were able to generate and purify K29-linked polyUb chains on a large scale and chains of defined lengths could be separated by cation-exchange chromatography (Figures 1E and 1F). Taken together, these data reveal a robust reproducible method for generating milligram quantities of K29 polyUb.

### Crystal Structures of K29-Linked diUb

**Figure 2:**
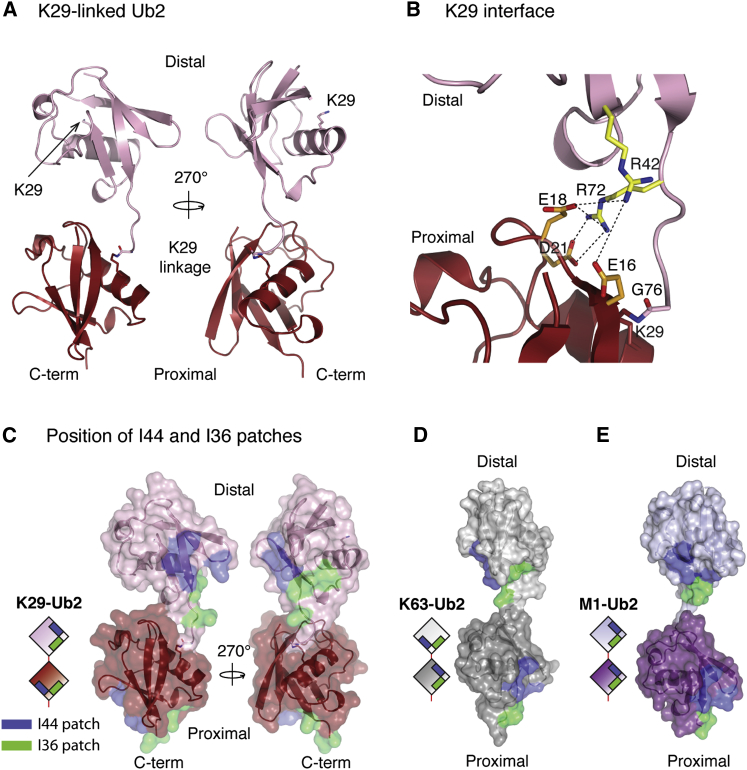
Crystal Structure of K29-Linked diUb (A) The crystal structure of K29-linked diUb is shown in two orientations. (B) Residues at the interface between the two Ub moieties are shown in stick representation. Polar interactions are shown as dotted lines. (C) Positioning of surface hydrophobic patches in the structure of K29-linked diUb. A semitransparent surface, colored blue for I44 patch (I44, L8, H68, and V70) and green for I36 patch (I36, L71, and L73), shows that the hydrophobic patches are not involved in the interface. (D and E) Relative orientations of hydrophobic patches in K63-linked (D) and M1-linked (E) diUb. A semitransparent surface as in (C), showing the I36 and I44 patches (PDB accession numbers 2JF5 and 2W9N) (Komander et al., 2009). See also Figure S2.

We crystallized K29-linked Ub dimers, and the structure was determined by molecular replacement and refined to the final statistics as shown in Table 1. The asymmetric unit (ASU) contains two K29-linked diUb molecules (Figure S2A). The distal Ub is bound via its C terminus to K29 of the proximal Ub, and there is visible electron density for the isopeptide linkage (Figure S2B). In the crystal structure, K29-linked diUb adopts an open conformation (Figure 2A). Contacts between the two isopeptide-linked Ub molecules are entirely polar and involve R42 and R72 on the distal Ub and E16, E18, and D21 of the proximal Ub (Figure 2B). The crystal structure reveals an extended conformation, but in solution, K29 chains may adopt other conformations.

This open conformation of K29 diUb is distinct from the completely extended conformation observed for K63 and M1 chains, where there is no contact between the two Ub moieties besides the linker (Figures 2C–2E). The hydrophobic I44 patch (made up of L8, I44, H68, and V70) and the I36 patch (made up of I36, L71, and L73) facilitate the majority of interactions between Ub and UBDs. Like K63 and M1 chains, these hydrophobic patches are exposed and accessible in the observed K29 diUb structure (Figure 2C).

### Profiling Linkage Selectivity of UBDs

**Figure 3:**
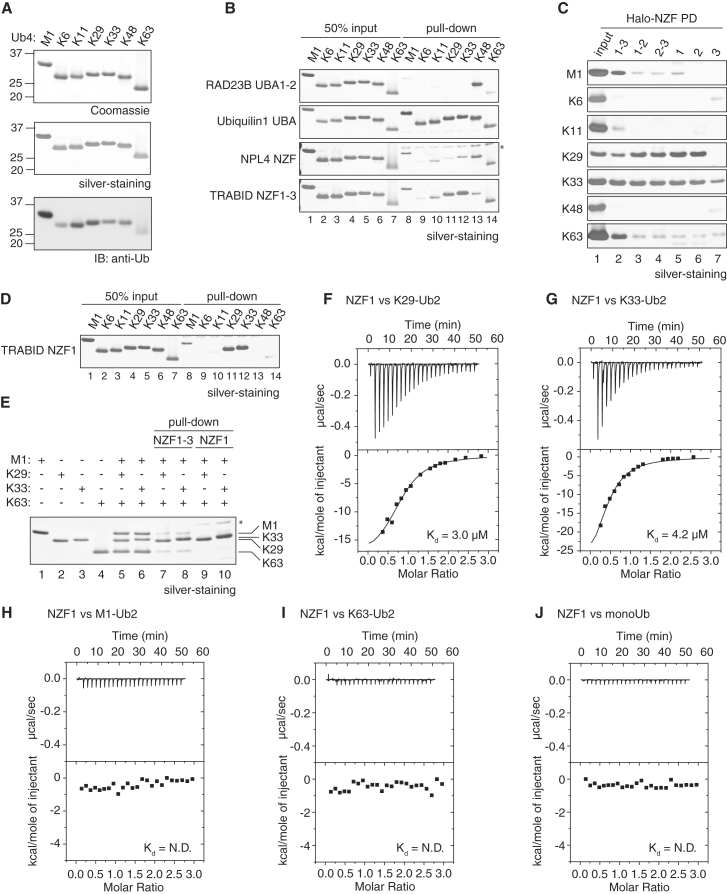
The NZF1 Domain of TRABID Is Highly Selective for K29 and K33 Chains (A) Purified tetraUb of M1, K6, K11, K29, K33, K48, and K63 linkages were separated on 4% to 12% SDS-PAGE gel and visualized by Coomassie and silver staining, and anti-Ub immunoblotting. (B) Linkage selectivity of different UBDs was investigated using pull-down assays. Halo-tagged RAD23B UBA1-2, Ubiquilin-1 UBA, NPL4 NZF, and TRABID NZF1-3 were used in pull-down assays with tetraUb of the indicated linkage types. The chains were visualized by silver-staining. Fifty percent of tetraUb input used in the pull-down assay was included as control. Asterisk shows NPL4 released from Halo resins. (C) Pull-down assays to determine the linkage selectivity of the individual or tandem NZF domains of TRABID. Halo-tagged fusions of the indicated TRABID NZF domains were used in pull-down assays with tetraUb of seven different linkage types. One hundred percent of tetraUb input used in the pull-down assay was included as control. (D) Linkage selectivity of TRABID NZF1 assayed as in (B). (E) Linkage preference of TRABID NZF domains was assayed using a competition pull-down assay. K29- or K33-linked tetraUb was mixed with M1- and K63-tetraUb chains, and pull-downs performed using Halo-TRABID NZF1-3 or NZF1. Asterisk shows NZF1 released from Halo beads. (F–J) ITC measurements for the NZF1 domain of TRABID with K29-Ub2 (F), K33-Ub2 (G), M1-Ub2 (H), K63-Ub2 (I), and monoUb (J). The *K*_d_ value for each measurement is indicated. See also Figure S3.

Because there are no UBDs characterized to date that bind to K29 chains, we next wanted to identify a UBD that can selectively recognize K29-linked chains. In order to systematically test linkage specificity of UBDs, we assembled and purified polyUb tetramers of seven different linkage types (Figure 3A). Each tetramer has a characteristic electrophoretic mobility, allowing distinction between linkage types (Figure 3A). Note that when equal amounts of the different tetramers are present as detected by Coomassie and silver staining, detection by the anti-Ub antibody varies across linkages. To assess the linkage preferences of UBDs, we expressed and purified them as Halo-fusion proteins (Figure S3A). We chose to use Halo-tagged UBDs in this study to characterize polyUb binding as this tag does not dimerize and circumvents the artifacts of tag-induced avidity (Sims and Cohen, 2009). We first analyzed the linkage preference of the proteasome shuttling protein RAD23B that has been well characterized as a K48 binding protein (Rao and Sastry, 2002; Varadan et al., 2005). When compared across seven linkage types, the UBA domains of RAD23B still exhibit exquisite preference for K48 chains (Figure 3B). In contrast, the UBA domain of Ubiquilin-1 (UBQLN1) is non-selective and binds to all linkages, while the NZF of NPL4 binds to all linkages with the exception of K6 and K29 linkages.

Both K29 and K33 linkages are hydrolyzed by the catalytic domain of the DUB TRABID (Licchesi et al., 2012). TRABID is a multi-domain protein with the catalytic domain at the C terminus and UBDs consisting of three tandem NZF domains at the N terminus. We hypothesized that TRABID uses the NZF domains for targeting to its substrates, namely K29 and K33 polyUb. Previous studies using two or three linkage types have shown that TRABID NZFs can bind to K63 and M1 chains (Komander et al., 2009; Tran et al., 2008). When we tested the tandem NZFs of TRABID (NZF1-3), we find that in addition to K63 and M1 chains that they were previously described to bind to, the tandem NZF1-3 domains also bind to K11, K29, and K33 chains (Figure 3B).

In order to test if the binding properties of the isolated NZF domains varied in comparison with the tandem repeats, we expressed Halo-fusions spanning permutations of NZF1-3 and individual domains (Figure S3A). In comparison with NZF1-3, the double repeat UBDs NZF1-2 and NZF2-3 showed reduced binding to K63 and M1 chains while maintaining binding to K29 and K33 chains (Figure 3C, lanes 1–4). The individual NZF1 and NZF2 domains showed selectivity toward K29 and K33 chains, binding most of the input tetraUb (Figure 3C, lanes 5 and 6). Interestingly, the binding properties of NZF3 were very different from those of NZF1 and NZF2, as it was able to bind to K33 chains and showed weak binding to K6, K48, and K63 chains but did not interact with K29 chains (Figure 3C, lane 7). In summary, the individual NZF1 and NZF2 domains were selective for K29 and K33 chains, whereas the tandem NZF1-3 domains were able to bind to multiple linkages.

We focused on the NZF1 of TRABID, which binds to K29 and K33 polyUb and pulls down small amounts of M1 and K63 chains (Figure 3D). Our data predict that in a mixed environment, NZF1 will bind preferentially to K29 and K33 chains. We therefore performed a direct competition experiment where a mixture of chains was used in the pull-down assay and the differences in electrophoretic mobility were used to distinguish the different linkage types. As predicted, NZF1 preferentially binds to K29 and K33 chains over K63 and M1 linkages (Figure 3E). We next performed isothermal titration calorimetry (ITC) to compare the binding affinities of the individual NZF domains for diUb of different linkage types. In agreement with the results observed with the pull-down experiments, NZF1 binds to K29- and K33-linked diUb with higher affinities, 3.0 and 4.2 μM, respectively, whereas no detectable binding was observed for monoUb, M1 or K63 diUb (Figures 3F–3J). Although NZF2 has similar binding affinities for K29 and K33 chains as NZF1, the affinity of NZF3 for K33-linked diUb is too low to be measured by ITC (Figures S3B–S3D). In summary, these results reveal that the NZF1 of TRABID preferentially binds to K29 and K33 chains over K63 and M1.

### Structural Basis for Linkage-Selective Recognition

To understand the structural basis of selective interaction between TRABID NZF1 and K29 chains, we determined the structure of TRABID NZF1 in complex with K29-linked diUb. Diffraction data were obtained at 3.0 Å resolution, and the structure was solved by molecular replacement and refined to the final statistics as shown in Table 1. TRABID NZF1 crystallized as a stoichiometric complex with K29 dimers, and the ASU contains five Ub and five NZF molecules (Figure S4A). The Ub moieties are arranged in such a way that the C terminus of one Ub points toward K29 of the next to form a continuous polyUb chain, and each NZF recognizes one diUb (Figures S4A and 4A). In the crystal lattice, this arrangement allows the chain to be extended infinitely and gives it the appearance of a helical filament (Figure S4A). The complexes found in the ASU superpose with a root-mean-square deviation (rmsd) between 0.363 and 0.883 (Figure S4B).

**Figure 4:**
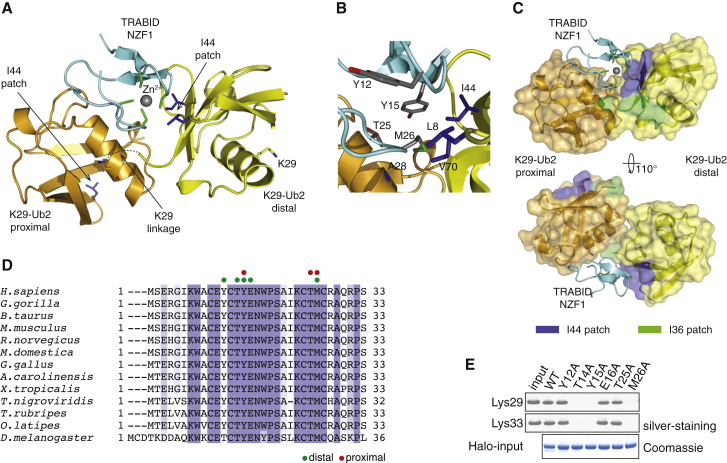
Crystal Structure of TRABID NZF1 in Complex with K29-Linked diUb (A) Structure of TRABID NZF1 (cyan; zinc as gray sphere coordinated by cysteine residues [green]) bound to diUb (orange, proximal Ub; yellow, distal Ub). I44, V70, and L8 are shown as blue sticks. (B) Selected residues of TRABID NZF1 (gray) and K29-diUb (blue) in the interface are shown. (C) A semitransparent surface shows that only the Ile44 patch (blue) of the distal diUb interacts with TRABID NZF1. (D) Sequence alignment of TRABID NZF1 from different species. Green and red circles indicate residues of TRABID NZF1 involved in binding to the distal and proximal Ub, respectively. (E) Residues of TRABID NZF1 involved in Ub binding were mutated to alanine and the binding to K29- and K33-tetraUb was assayed as in Figure 3C. Halo-fusion proteins coupled to the resin were visualized on Coomassie-stained SDS-PAGE gel. See also Figure S4.

The structure of the NZF1 of TRABID is almost identical to the NZF domains of NPL4, TAB2, TAB3, and HOIL-1L (rmsd < 0.9 Å) (Alam et al., 2004; Kulathu et al., 2009; Sato et al., 2011, 2009). The four cysteine residues of TRABID NZF1, C10, C13, C24, and C27, coordinate a zinc ion. The TRABID NZF binds to the distal and proximal Ub moieties with buried surface areas of 350 and 320 Å^2^, respectively. Although TRABID NZF1 interacts with both proximal and distal Ub, it does not directly recognize the K29-linked isopeptide bond of the diUb. The NZF domain of TRABID interacts with the I44-centered hydrophobic patch on the distal Ub (Figures 4B and 4C). In most NZFs, distal Ub recognition is mediated by a hydrophobic patch centered on the consensus T-F motif (T14 and Y15 in TRABID NZF1) (Alam et al., 2004; Haglund and Dikic, 2005) (Figure 4B). An additional hydrophobic residue present 10 amino acids away from the T-F motif (M26 in NZF1) also mediates interactions with the I44 patch on the distal Ub (Figures 4B and 4D). Thus in the NZF1 of TRABID, residues T14, Y15, and M26 interact with the distal Ub, and these residues are conserved in evolution (Figure 4D). Indeed, mutation of T14, Y15, or M26 to alanine is sufficient to disrupt the binding of TRABID NZF1 to K29 and K33 chains (Figure 4E).

**Figure 5:**
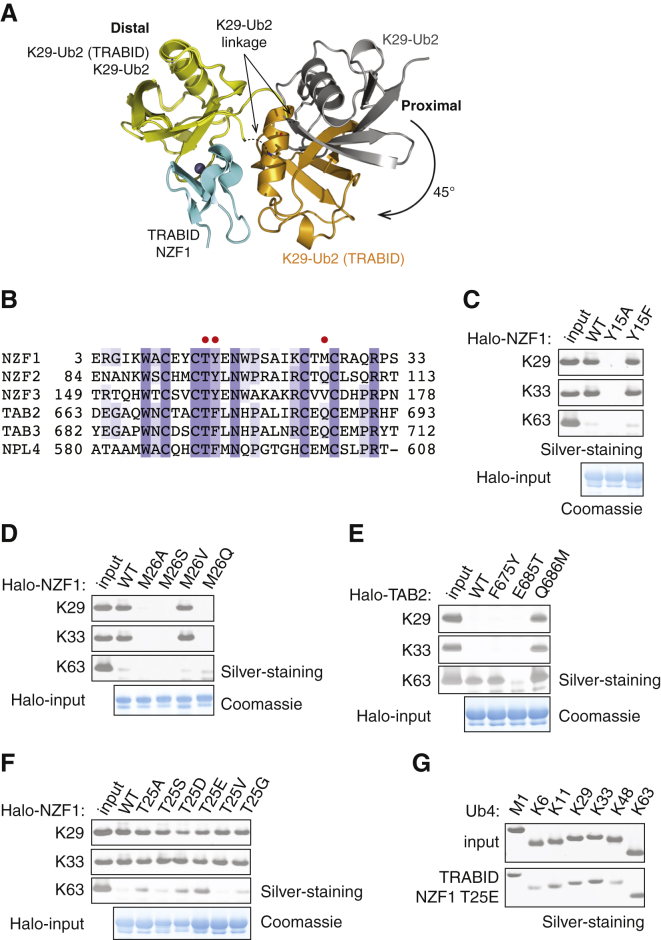
Determinants of NZF Binding to PolyUb (A) Superposition of the distal Ub moieties (yellow) of K29-diUb alone and K29-diUb in the complex with TRABID NZF1. In the complex, the proximal moiety of free K29-diUb (gray) rotates approximately 45° to interact with NZF1 (cyan). (B) Sequence alignment of TRABID NZF1, NZF2, and NZF3 with NZF domains of TAB2, TAB3, and NPL4. The hydrophobic residues making up the T-F/Φ binding patch are highlighted by red spheres. (C and D) TRABID NZF1 mutants Y15 (C) and M26 (D) were assayed for binding to K29, K33, and K63 tetraUb. (E) TAB2 NZF mutants were assayed as in (C). (F) T25 of TRABID NZF1 was mutated and polyUb binding was assayed as in (C). (G) Linkage specificity of TRABID NZF1 T25E mutant was assayed as in Figure 3B. See also Figure S5.

In contrast to other NZF domains, TRABID NZF1 does not make contacts with either the I44 or F4 hydrophobic patches on the proximal Ub (Figures 4C and S5). Instead, TRABID NZF1 recognizes residues around K29 in the proximal Ub. Interestingly, TRABID M26 makes contacts with both the distal and proximal Ub moieties, and mutation of this residue disrupts NZF1 binding to K29 chains (Figures 4E and S3E). The overall conformation and relative orientation of the distal and proximal Ub moieties of the K29-linked diUb bound to TRABID NZF1 are considerably different from that of free K29-linked diUb (Figure 5A). When superposed on the distal Ub, the proximal Ub of the complex is rotated by 45° and moved by around 20 Å, thus remodeling the K29 chain. This remodeling enables TRABID NZF1 to make simultaneous interactions with both the distal and proximal moieties. A network of hydrogen bonds and ionic interactions further stabilizes the complex and is summarized in Table S3. TRABID NZF1, thus binds to K29 chains by binding to the I44 patch on the distal Ub and the Ub helix in the proximal Ub.

Linkage selective polyUb recognition requires simultaneous binding of the NZF to both distal and proximal moieties. If the binding of the NZF to the distal Ub is too strong, then polyUb binding will not depend on contribution from the proximal Ub, and hence polyUb binding will not be linkage selective. An example is the NZF domain of NPL4 that binds to monoUb via a distal Ub recognition site made up of T590, F591, and M602 and is therefore non-selective (Figures 3B and 5B) (Alam et al., 2004). In contrast, the NZF domain of TAB2 or TAB3 and TRABID NZF1 cannot bind to monoUb, as they have sub-optimal distal Ub recognition sites made up of T674, F675, and Q686 (TAB2) or T14, Y15, and M26 (TRABID) (Figure 3J) (Kulathu et al., 2009). Indeed, TRABID-NZF1 requires hydrophobic residues at each of these positions to bind polyUb (Figures 5C and 5D). As expected, strengthening distal Ub binding by mutating the hydrophilic Q686 of TAB2 to methionine (Q686M) not only increases binding of the mutant NZF to K63 chains but also enables it to bind to K29 and K33 chains (Figure 5E).

To address the importance of proximal Ub recognition, we focused on residue T25 of TRABID-NZF1. Although mutation of T25 to alanine does not affect TRABID-NZF1 binding to K29 or K33 chains (Figure 5F), the corresponding residue in TAB2 (E685) interacts with the proximal Ub and is important for binding K63 chains (Kulathu et al., 2009; Sato et al., 2009). This raises the possibility that the type of residue present at this position would influence specificity. Indeed, some T25 mutants show additional binding to K63 chains, suggesting that the residue at this position may influence linkage selectivity (Figure 5F). Interestingly, introducing a negatively charged residue at this position converts TRABID-NZF1 to be able to bind to multiple linkage types (Figure 5G). In summary, achieving linkage-selective polyUb recognition requires binding to the distal Ub with modest affinity and linkage-dependent interactions with the proximal Ub.

### Heterotypic PolyUb Containing K29 Linkages Are Captured by TRABID

**Figure 6:**
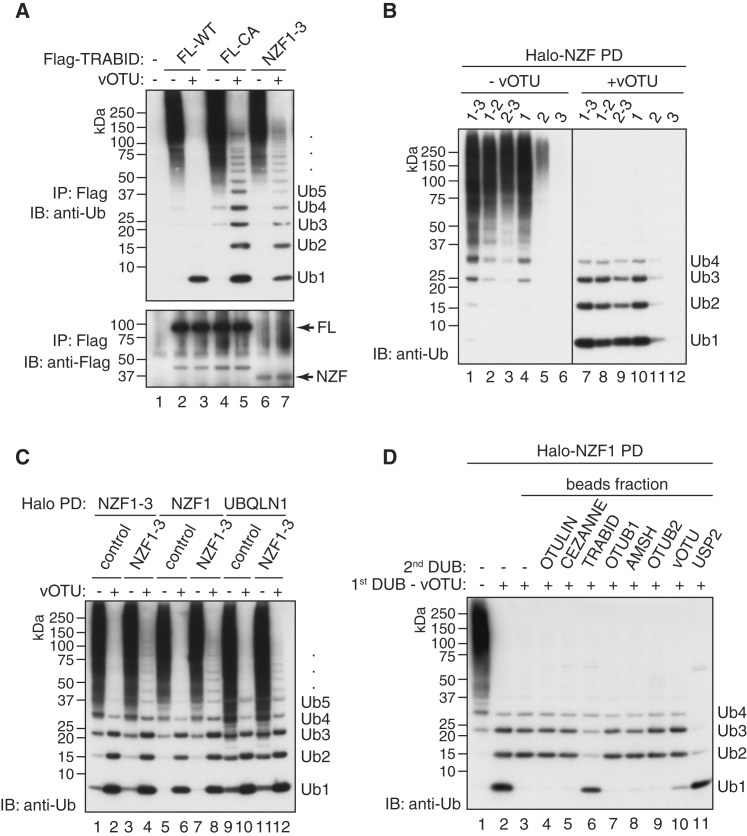
Isolation and Analysis of K29 Chains from Mammalian Cells (A) Analysis of polyUb captured from HEK293 cells transiently expressing Flag-tagged full-length TRABID (FL-WT), full-length catalytically dead (FL-CA), or tandem NZF1-3 domains (NZF1-3). One half of the Flag-immunopurified sample was incubated with vOTU that hydrolyzes all linkage types excepting M1, K27, and K29 linkages and visualized by anti-Ub immunoblotting. (B) PolyUb chains from HEK293 extracts were captured using indicated bacterially expressed Halo NZF domains of TRABID, followed by DUB treatment as in (A). (C) PolyUb chains from untransfected (control) or Flag-TRABID NZF1-3 (NZF1-3)-expressing HEK293 cells were captured using Halo-TRABID NZF1-3, TRABID NZF1, or Ubiquilin-1 (UBQLN1) UBA domain. The isolated polyUb chains were either left untreated or treated with vOTU. (D) Immunobloting analysis of polyUb chains captured from HEK293 extracts using the minimal NZF1 that is selective for K29 and K33 linkages. Presence of K29 linkages was assayed by incubation with vOTU, which does not hydrolyze K29 linkages. The vOTU-resistant polyUb chains that bound to Halo-TRABID NZF1 were separated from monoUb, washed, and the linkage-types of these chains were assayed by incubation with a panel of DUBs that hydrolyze different linkage types. See also Figures S6 and S7.

K29 linkages can be detected in proteomic studies (Dammer et al., 2011). Therefore, we hypothesized that the selective recognition of these linkages by TRABID could be exploited to capture K29 chains from cells. To test this hypothesis, we transiently expressed Flag-tagged versions of different TRABID constructs in HEK293 cells and analyzed the polyUb chains captured from cell extracts by TRABID (Figure 6A). Although full-length wild-type TRABID will bind to K29 linkages but hydrolyze them, the catalytically inactive mutant C443A should bind to K29 chains but not hydrolyze them (Figure S6A). Both full-length TRABID and the tandem NZFs capture high-molecular mass polyUb from resting cells (Figure 6A, lanes 2, 4, and 6). To detect if K29 linkages are present in the captured polyUb material, we incubated the pull-down with the DUB vOTU that does not hydrolyze M1, K27, and K29 linkages (Ritorto et al., 2014). Although incubation with vOTU reduced the high-molecular weight ubiquitylated species captured by wild-type TRABID down to monoUb, free polyUb chains resistant to vOTU cleavage were released from the TRABID C443A isolates (Figure 6A, lanes 3 and 5). Despite their poor expression in HEK293 cells, the tandem NZF domains of TRABID also captured ubiquitylated proteins and free polyUb chains of a similar pattern were released upon vOTU treatment (Figure 6A, lane 7, and Figure S6D).

To ensure that what we were observing was not an artifact of overexpressing TRABID domains in cells, we used bacterially expressed and purified Halo-fusions of TRABID NZFs to capture polyUb from cell extracts. Halo-NZF1-3, NZF1-2, and NZF2-3 all captured polyUb from cell extracts and released short chains when treated with vOTU (Figure 6B). Interestingly, the single NZF1 captured as much polyUb as the tandem fusion, while isolated NZF2 captured only a limited amount of polyUb (Figure 6B, lanes 4, 5, 10, and 11). However, the K33-selective NZF3 does not pull down any polyUb from cells.

We observed that only short chains are released from the Halo-NZF capture after vOTU treatment, in contrast to the longer chains released when the capture was done using Flag-tagged tandem NZF domains expressed in cells (Figures 6A and 6B). This may be because the Flag-tagged NZFs capture longer vOTU-resistant chains, or, alternatively, expression of the UBD in cells protected the chains from hydrolysis by cellular DUBs. To test this idea, we compared the polyUb that we captured from untransfected cells or from cells expressing NZF1-3 of TRABID. Further, we tested if our findings could be recapitulated using a different UBD, for which we used the UBA domain of Ubiquilin-1 (UBQLN1), which is a non-selective polyUb binder capable of binding to several linkage types (Figure 3B). Immobilized Halo-NZF1-3, NZF1, and UBQLN1 all captured polyUb from extracts of both untransfected and transfected cells (Figure 6C). When treated with vOTU, polyUb chains of defined lengths were released from the captured material and significantly longer vOTU-resistant chains were present in pull-downs when cells expressed NZF1-3 (Figure 6C).

We have established a method to investigate K29 chains in cells that relies on TRABID-NZF1 binding to K29 and K33 chains and subsequent cleavage with a DUB that does not hydrolyze K29 linkages (Figure S7A). However, because vOTU does not hydrolyze M1 and K27 linkages in addition to K29 linkages, the short Ub chains released by vOTU from the high-molecular weight ubiquitylated mixture could contain these linkages. In order to determine the exact linkage type, we used linkage-selective DUBs. The polyUb species captured from HEK293 cell extracts using Halo-TRABID NZF1 beads were first incubated with vOTU. Interestingly, these short vOTU-resistant chains stayed bound to the Halo-TRABID NZF1 bead fraction, while the supernatant contained only monoUb (Figures 6D and S6B). The bead fraction containing the short chains was first washed to remove vOTU along with any of the released monoUb and then subsequently incubated with different linkage-selective DUBs. USP2, a linkage promiscuous DUB, hydrolyzed these short chains to monoUb, while DUBs that cleave M1 (OTULIN), K11 (CEZANNE), K48 (OTUB1), and K63 (AMSH) did not disassemble these short chains (Figure 6D). Incubation with TRABID cleaved these chains down to monoUb, indicating that the vOTU-resistant chains are K29-linked. Using pRM LC-MS/MS analysis, we further confirmed that these short vOTU resistant chains isolated from cell extracts are indeed K29-linked (Figures S7B and S7C).

### K29 Linkages Are Present in Heterotypic Chains

**Figure 7:**
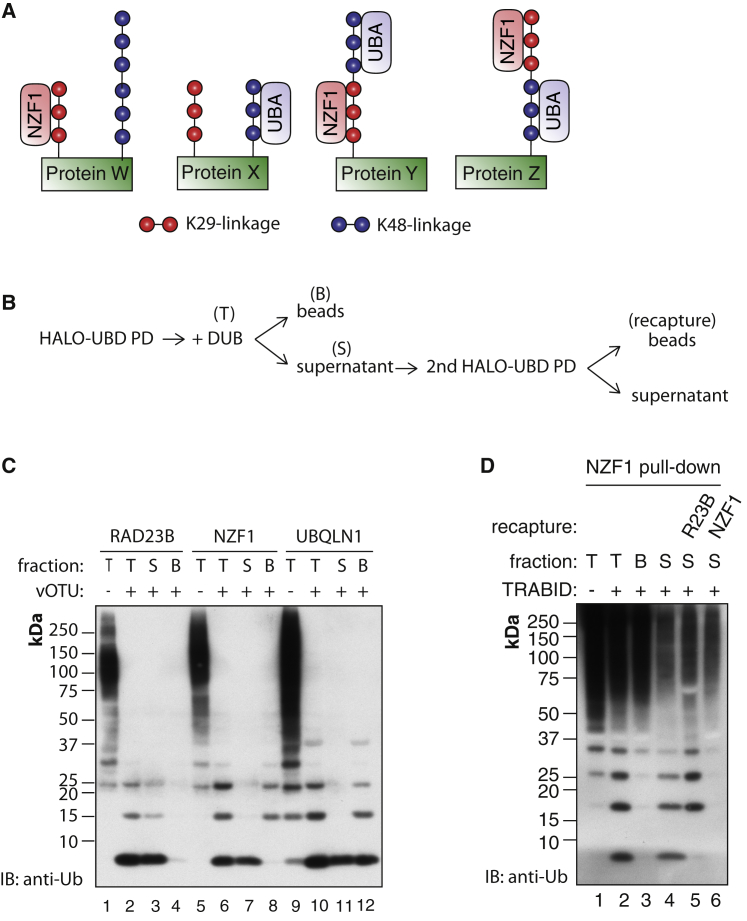
K29-Linked Chains Are Present within Mixed or Branched Heterotypic Chains (A) Cartoon illustrating the different possibilities of how K29 linkages may be present in the heterotypic chains captured by TRABID NZF1. (B) A schematic illustrates the experimental procedure for determining the linkage composition of the mixed or branched heterotypic chains isolated by the linkage-selective UBDs. (C) PolyUb chains from the HEK293 were first captured using Halo-tagged RAD23B (K48-selective binder), TRABID NZF1 (K29- and K33-selective binder), or UBQLN1 (non-selective binder). Subsequently, samples in the indicated lanes were treated with vOTU. The total capture (T), supernatant (S), and beads (B) fractions after the DUB reaction were analyzed separately as shown in (B). (D) Recapture assay using linkage-selective UBDs to determine the linkage type of the chains remaining after removing K29 linkages. PolyUb chains captured from HEK293 cell extracts using Halo-NZF1 were incubated with TRABID that preferentially hydrolyzes K29 and K33 linkages. The polyUb chains that were released into the supernatant fraction after removing K29 linkages were recaptured using either the K48-selective Halo-RAD23B or the K29 and K33 selective Halo-TRABID NZF1. See also Figure S8.

The above experiments reveal that NZF1 captures K29 polyUb present in cells and suggest that these K29 linkages are present in heterotypic chains containing other linkages. We observe similar results when we perform pull-downs from extracts of different mouse tissues, suggesting that these heterotypic chains may be ubiquitous (Figure S8A). With available methods, it is challenging to determine the topology of these chains. Nevertheless, we used a combination of linkage-selective UBDs and DUBs to investigate the different possibilities of how these chains may be present (Figures 7A and 7B). To test if these heterotypic chains are made up of K48 and K29 linkages, we used Halo-RAD23B fusion that binds selectively to K48 chains to capture polyUb from cell extracts and the captured material was subsequently treated with vOTU (Figure 7C). Similar to what we observe with TRABID NZF1, short vOTU-resistant K29 chains were released, but in contrast to NZF1, these chains were not bound to RAD23B but are instead present in the supernatant (Figure 7C, lanes 3 and 4). Although short chains are also released from TRABID NZF1 captured material, these chains remain bound to the beads and the supernatants contained only monoUb (Figure 7C, lanes 7 and 8). Similar results were observed when the non-specific UBD of UBQLN1 was used (Figure 7C, lanes 11 and 12). We confirmed that these short chains are K29 linked, as subsequent incubation of these short chains with the DUB TRABID results in their hydrolysis down to monoUb (Figure S8C). Collectively, these results demonstrate that when we isolate polyUb chains from cells using a K29-selective UBD or a K48-selective UBD, we can detect the presence of short K29 chains.

In a converse experiment, we first captured polyUb from cell extracts using TRABID NZF1 and treated the captured material with TRABID that hydrolyzes K29 and K33 linkages (Figure 7D). The chains released into the supernatant were then subsequently incubated with Halo resin containing either RAD23B or TRABID NZF1. Importantly, the released chains bound only to RAD23B and not to TRABID NZF1.

To provide further evidence for the existence of heterotypic chains containing K29 linkages, we performed serial polyUb capture experiments (Figures S8D and S8E). When TRABID NZF1 was used in the first pull-down, this captured all the K29-linked chains from cells. A second pull-down using a K48-specific UBD captured polyUb from the cell extracts, but these did not contain any K29 linkages (Figure S8D). In a converse experiment, if the first capture was done with a K48-specific UBD, all the K29 chains were present in this fraction, and no further K29 chains could be isolated in a subsequent capture with a K29-specific UBD (Figure S8D). These data further support our hypothesis that K29 linkages are present in chains that also contain K48 linkages. Moreover, these results also suggest the existence of two pools of K48 chains, one with and another without K29 linkages. Taken together, these data show that multiple blocks of K29-linked polyUb exist in cells as part of heterotypic chains that also contain K48 linkages.

## Discussion

K29 linkages are an abundant atypical linkage type, but their cellular roles are not completely understood, as tools such as linkage-specific antibodies are not available to study these chains. Our work shows that K29-linked polyUb adopt an open conformation and are recognized by the NZF domains of TRABID and has revealed the use of NZF1 of TRABID as a tool to study K29-linked polyUb in cells. Importantly, the use of this tool has shown that K29 chains exist as heterotypic chains exclusively, mainly containing K48 linkages, and furthermore that K48 chains exist in two distinct pools, one containing K29 linkages and one without.

The ability to enzymatically assemble K29 chains in vitro is an advance that will pave the way for many subsequent studies. For the enzymatic assembly of K29 polyUb chains, we have combined the HECT ligase UBE3C with the DUB vOTU. Although we have synthetically created a Ub chain-editing complex for our purpose, such complexes containing ligases and DUBs have been described in vivo. These complexes are an efficient mechanism to rapidly remodel the topology of polyUb on a substrate and thereby modulate the outcome of the Ub signal (Newton et al., 2008; Wertz et al., 2004). Interestingly, several DUBs are found associated with Ub ligases (Sowa et al., 2009), and we speculate that these uncharacterized complexes may assemble atypical chains in cells.

UBE3C, the enzyme used for assembling K29 chains, is involved in regulating protein turnover (Chu et al., 2013). Recent work demonstrated that UBE3C associates with the proteasome and its levels at the proteasome increase upon proteasome inhibition (Besche et al., 2014; Crosas et al., 2006). In yeast, the UBE3C homolog Hul5 functions as an E4 to extend Ub chains on ubiquitylated substrates to promote degradation of misfolded proteins (Fang et al., 2011). Such an extension of polyUb on substrates at the proteasome by UBE3C may increase the residence time of the substrate at the proteasome and leads to the speculation that K29-K48 heterotypic chains provide an important signal for difficult to degrade substrates to be proteolyzed.

The assembly of K29 chains has not only allowed us to structurally characterize these linkages but has also provided insights into their recognition and clues into their biological roles. Until recently, K48 and K63 chains were the only linkage types that could be assembled in vitro, which permitted better analyses of these linkages compared with the remaining six linkages. With the availability of tetraUb of seven linkage types, we can now systematically profile UBDs to reveal their polyUb binding characteristics. The NZF domains of TRABID were previously described as selective K63 and M1 binders, but the availability of tetraUb of seven different linkage types has allowed a more complete assessment of linkage selectivity and led us to discover that the individual NZFs of TRABID interact specifically with K29 and K33 polyUb. Excitingly, this provides the description of a UBD that is selective for these uncharacterized linkages.

In all the structural studies analyzing polyUb binding by NZF domains, the NZF does not make direct contact with the linker between the two Ub moieties but rather exploits the relative orientation of surface hydrophobic patches on Ub (Husnjak and Dikic, 2012). NZF1 of TRABID remodels the open conformation of K29 chains to satisfy a two-sided interaction. In contrast to other NZF domains that recognize the proximal Ub via one of the hydrophobic patches, TRABID NZF1 uses a distinct mode of Ub recognition whereby it makes contacts with the helix in Ub. Given that both K29 and K33 lie on the Ub helix, we speculate that NZF1 and NZF2 may use similar mechanisms to bind to both K29 and K33 chains. In support of this idea, we find that mutations that disrupt binding to K29 chains also affect binding to K33 chains. In tandem, the UBDs have a different polyUb binding preference that could be explained in part by the relative spatial organization of the domains, using a mechanism similar to that described for the tandem UIMs of RAP80 (Sims and Cohen, 2009).

Our results suggest that rheostat-like modulation of the affinity of the NZF for distal and proximal Ub moieties is central to achieving linkage-selective polyUb recognition. NZF domains like that of NPL4 can bind strongly to the distal Ub, thus making them non-specific binders. In contrast, NZF domains like that of TAB2 and TRABID, which have weaker binding to distal Ub, now rely on additional interactions mediated with the proximal Ub, and these interactions with the proximal Ub are determinants of linkage-selective recognition. It is tempting to speculate that by modulating the proximal Ub binding site designer NZFs specific for desired linkage types can be created.

The availability of linkage-specific antibodies and polyUb sensors has greatly advanced our understanding of the cellular roles and the spatiotemporal dynamics of M1, K11, K48, and K63 polyUb in cells (Matsumoto et al., 2010; Newton et al., 2008; van Wijk et al., 2012). We exploited our identification of UBDs that selectively bind to these two linkages to use these NZFs as an affinity reagent to capture K29 cells from cells. In agreement with previous proteomic analyses, we can detect K29 linkages in resting mammalian cells and in different mouse tissues. Unexpectedly, we find that K29 chains are present as heterotypic chains. It has only recently emerged that mixed and branched polyUb chains serve specialized signaling functions (Emmerich et al., 2013; Meyer and Rape, 2014). Although we have been able to readily isolate heterotypic chains containing short K29-linked polyUb containing no more than four moieties, longer K29 chains were detected only when the tandem NZFs of TRABID was expressed in cells. This suggests that K29 chains do not grow very long and are hydrolyzed by cellular DUBs. Because several proteomic analyses find that the abundance of K29 linkages increases upon proteasomal inhibition, we speculate that short K29-linked chains may be added as capping modifications. Further, the helical nature of K29 polymers, as observed in the structure of K29 chains in complex with TRABID NZF1, may denote a specialized signal.

Determining the precise topology of these heterotypic polyUb chains and whether they are present as mixed or branched chains will be challenging. It will be of great interest to investigate the conditions under which these chains are made in cells and the precise biological processes that are regulated by these heterotypic chains. Given the paucity of tools available to study different linkage types, our work reveals how linkage selective UBDs can be exploited to capture particular linkages from cells and provides a valuable tool to investigate cellular roles of this enigmatic linkage.

## Experimental Procedures

An extended version can be found in Supplemental Experimental Procedures.

### K29-Linked PolyUb Assembly

Large-scale K29-linked polyUb chain assembly was carried out in 1.5-ml reactions of 25 mg Ub (Sigma), 644 nM UBE1, 9.5 μM UBE2D3, 3 μM UBE3C, 10 mM ATP, 50 mM Tris-HCl (pH 7.5), 10 mM MgCl_2_, and 0.6 mM DTT at 30°C overnight. To release K29-polyUb chains, a total of 2 μM vOTU and 5 mM DTT were added in to the assembly reaction and incubated further at 30°C overnight. Chains of defined lengths were purified as described in Supplemental Experimental Procedures.

### Preparation of Halo-UBD Resins

UBDs used in this study were expressed in *E. coli* as GST-Halo fusion protein (see Table S1). GST was removed using C3 protease. Halo-tagged UBDs (21 nmol) were incubated with 200 μl of the HaloLink resin (Promega) in 1 ml binding buffer (50 mM Tris-HCl [pH 7.5], 150 mM NaCl, 0.05% NP-40, 1 mM DTT) overnight at 4°C. Pull-down and analysis of polyUb chains using Halo-tagged UBDs were performed as described in Supplemental Experimental Procedures.

## Author Contributions

Y.A.K. and Y.K. designed, performed, and analyzed all experiments. S.A.A.R. performed crystallographic analyses. D.G.C. and N.A.M. did the mass spectrometry analyses. C.J. helped with protein purification. R.T. cloned all the DNA constructs. Y.K. wrote the manuscript with input from all authors.

## Figures and Tables

**Table 1:** Data Collection and Refinement Statistics

Data Collection	K29-linked diUb	K29-linked diUb–TRABID NZF1 complex
Wavelength (Å)	1.033	0.9795
Resolution range (Å)	60.05–2.30 (2.38–2.30)	76.10–3.00 (3.14–3.00)
Space group	P2_1_	C2
Unit cell		
a, b, c (Å)	33.45 69.25 60.06	99.22 123.97 78.31
α, β, γ (°)	90.00 90.22 90.00	90.00 103.68 90.00
Total reflections	22,801 (2,317)	61,923 (5,809)
Unique reflections	11,542 (1,185)	17,755 (1,691)
Multiplicity	1.9 (2.0)	3.5 (3.4)
Completeness (%)	94.12 (92.88)	98.97 (95.32)
I/σI	8.71 (3.07)	15.64 (2.05)
R_merge_	0.09621 (0.4483)	0.07207 (0.4869)
CC1/2	0.977 (0.614)	0.998 (0.914)

**Refinement**		

Number of atoms		
Protein	2,366	3,622
Ligand/ion	16	5
Water	43	0
R_work_/R_free_	0.1981/0.2456	0.2222/0.2702
Rmsd		
Bond lengths (Å)	0.018	0.005
Bond angles (°)	1.94	0.84
Average B-factor (Å^2^)	29.2	81.9

The highest resolution shell is shown in parentheses.

## Accession Numbers

The Protein Data Bank (PDB) accession numbers for the structures reported in this paper are 4S22 and 4S1Z.

## Supplemental Information

Document S1. Supplemental Experimental Procedures, Figures S1–S8, and Tables S1–S4

Document S2. Article plus Supplemental Information
