# Supplementary material for: K29-Selective Ubiquitin Binding Domain Reveals Structural Basis of Specificity and Heterotypic Nature of K29 Polyubiquitin
Source: Mol Cell. 2015 Apr 2;58(1):83–94. doi: 10.1016/j.molcel.2015.01.041 (PMC4386640; doi:10.1016/j.molcel.2015.01.041)
Supplement: Document S1. Supplemental Experimental Procedures, Figures S1–S8, and Tables S1–S4 [file mmc1.pdf]

Molecular Cell, Volume *58*

Supplemental Information

K29-Selective Ubiquitin Binding Domain Reveals Structural Basis of Specificity and Heterotypic Nature of K29 Polyubiquitin

Yosua Adi Kristariyanto, Syed Arif Abdul Rehman, David G. Campbell, Nicholas A. Morrice, Clare Johnson, Rachel Toth, and Yogesh Kulathu

K29-selective ubiquitin binding domain reveals structural basis of specificity and heterotypic nature of K29 polyubiquitin

Yosua Adi Kristariyanto, Syed Arif Abdul Rehman, David G. Campbell, Nicholas A. Morrice[1], Clare Johnson, Rachel Toth & Yogesh Kulathu[#]

Supplemental Information

MRC Protein Phosphorylation and Ubiquitylation Unit, College of Life Sciences, University of Dundee, Dow Street, Dundee DD1 5EH, UK
[1]Present address: AB Sciex UK Limited, Warrington, Cheshire WA1 1RX, UK

[#]Correspondence should be addressed to Yogesh Kulathu, MRC Protein Phosphorylation and Ubiquitylation Unit, College of Life Sciences, University of Dundee, Dow Street, Dundee DD1 5EH, UK.
Email: ykulathu@dundee.ac.uk, Tel: +44 1382 388163, Fax: +44 1382 223778

Figure S1

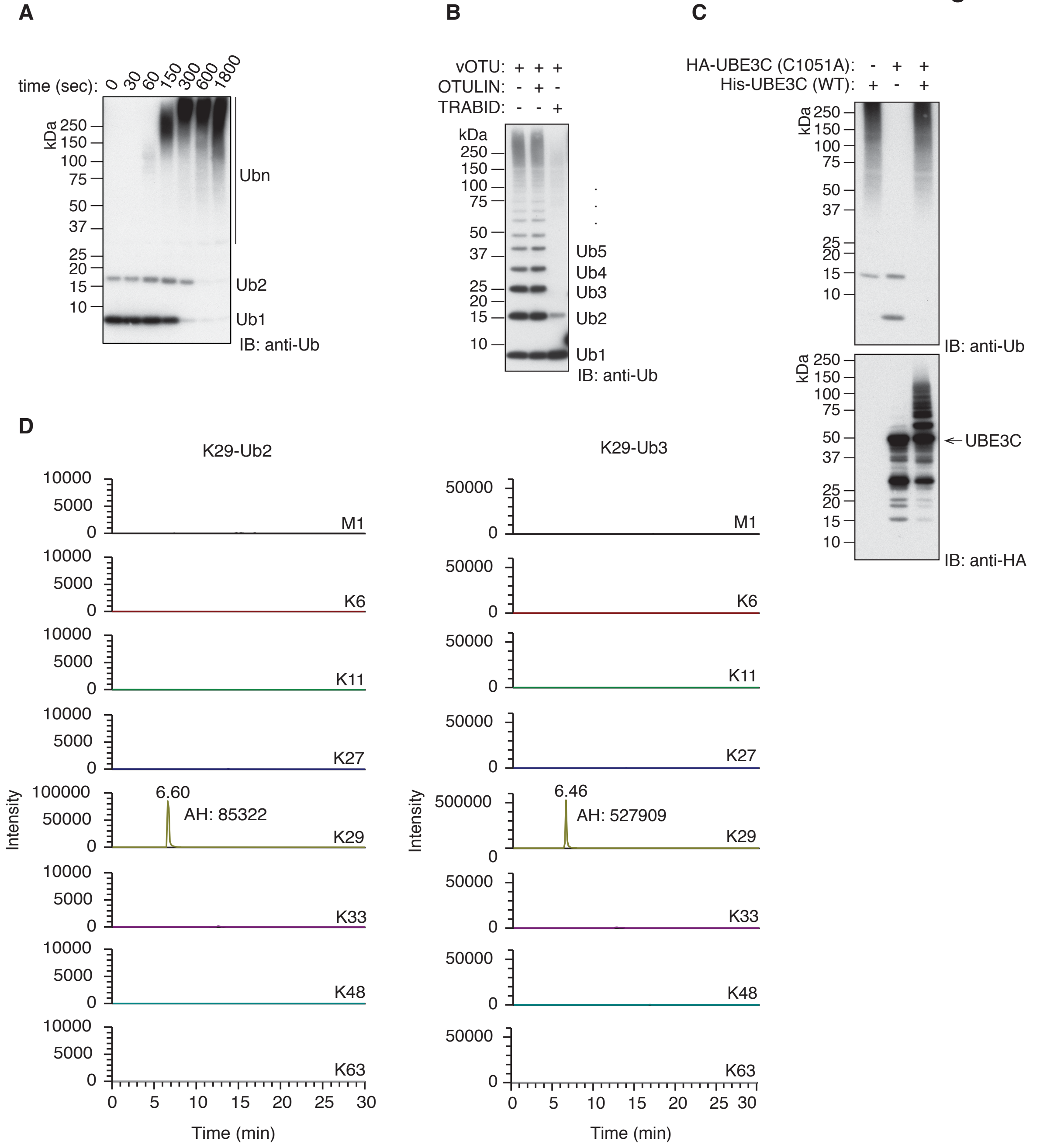

A
time (sec): 0 30 60 150 300 600 1800
kDa
250
150
100
75
50
37
25
20
15
10
Ubn
Ub2
Ub1
IB: anti-Ub
B
vOTU: + + +
OTULIN: - + -
TRABID: - - +
Ub5
Ub4
Ub3
Ub2
Ub1
IB: anti-Ub
C
HA-UBE3C (C1051A): - + +
His-UBE3C (WT): + - +
IB: anti-Ub
UBE3C
IB: anti-HA
D
K29-Ub2
K29-Ub3
M1
K6
K11
K27
K29
K33
K48
K63
Intensity
6.60
AH: 85322
6.46
AH: 527909
Time (min)

Figure S1. Polyubiquitin chains assembled using UBE3C and vOTU are K29-linked, Related to Figure 1.

(**A**) Ubiquitylation kinetics of UBE3C assayed using *in vitro* ubiquitylation reactions containing UBE1, UBE2D3, UBE3C, and Ub were incubated for the indicated time periods.

(**B**) Ubiquitylation assays of UBE3C containing UBE1, UBE2D3, UBE3C and Ub were performed in the presence of vOTU, M1 linkages-specific OTULIN, and K29 linkages-specific TRABID as indicated. The vOTU resistant chains are not hydrolyzed by OTULIN but are cleaved by TRABID.

(**C**) UBE3C is ubiquitylated in *trans.* Ubiquitylation reactions were performed using His tagged UBE3C wild type or HA-UBE3C C1051A or containing both together. Ubiquitylation of catalytically dead UBE3C C1051A was visualized by anti-HA immunoblotting (bottom panel).

(**D**) Parallel Reaction Monitoring (pRM) analysis of seven different Ub linkages in the purified K29-diUb and triUb (see **Figure 1F**). To highlight the purity of the K29-linkage and the absence of other linkages, the intensity scale for the non-K29 linkages was set 10-fold lower. AH: refers to automated height as determined by the XCalibur software.

Figure S2

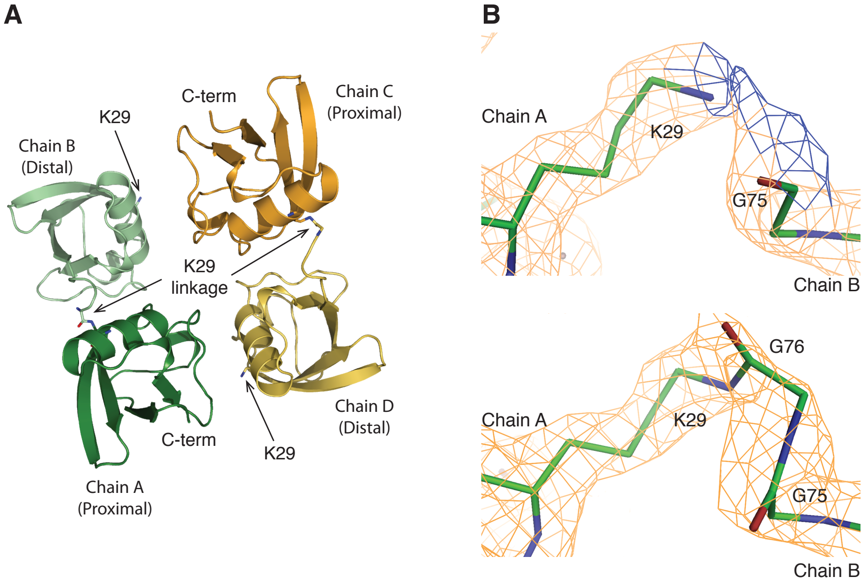

Figure S2. Structure of K29-linked diUb, Related to Figure 2.

(**A**) The asymmetric unit contains four Ub molecules and makes up two K29-linked dimers. Cartoon representation of one dimer (Chain A and B) is colored in shades of green and the other dimer (Chain C and D) is colored in shades of orange. The overall arrangement of the distal and proximal Ub moieties in both K29-linked diUb molecules present in the ASU are similar and both dimers adopt the same conformation.

(**B**) Electron density for the K29 linkage in the diUb structure. Electron density maps (orange represents $2|F_o|-|F_c|$ contoured at 0.7σ; blue represents $|F_o|-|F_c|$ contoured at 3σ) spanning K29 of the proximal Ub and the C-terminus of Ub before (top) and after (bottom) refinement with the isopeptide linkage. The linker region containing the isopeptide linkage has high temperature factors indicative of a high degree of flexibility.

Figure S3

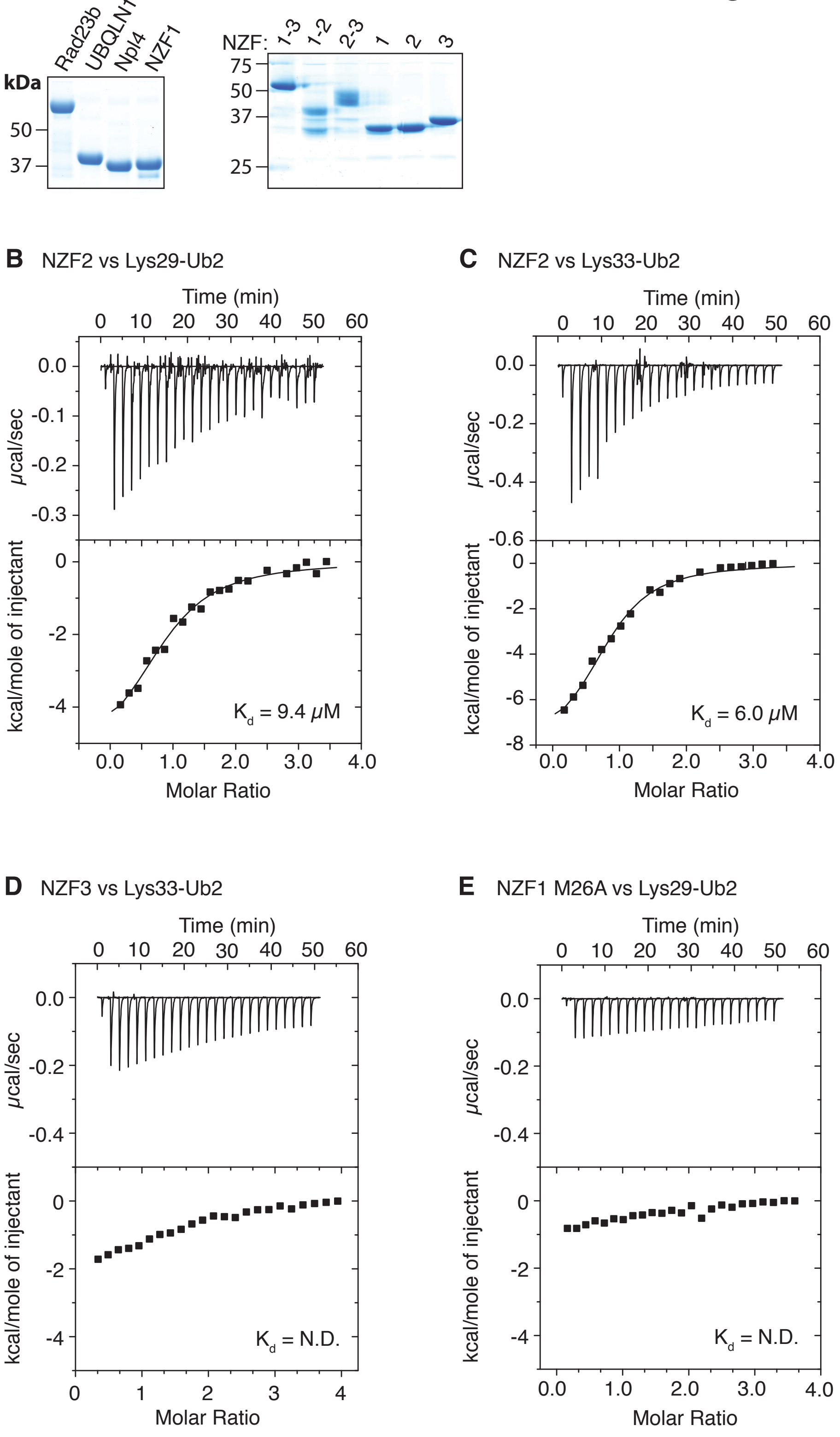

A
kDa
Rad23b
UBQLN1
Npl4
NZF1
50
37
NZF:
1-3
1-2
2-3
1
2
3
75
50
37
25
B NZF2 vs Lys29-Ub2
Time (min)
0 10 20 30 40 50 60
0.0
-0.1
-0.2
-0.3
µcal/sec
0
-2
-4
kcal/mole of injectant
K_d = 9.4 µM
0.0 1.0 2.0 3.0 4.0
Molar Ratio
C NZF2 vs Lys33-Ub2
Time (min)
0 10 20 30 40 50 60
0.0
-0.2
-0.4
-0.6
µcal/sec
0
-2
-4
-6
-8
kcal/mole of injectant
K_d = 6.0 µM
0.0 1.0 2.0 3.0 4.0
Molar Ratio
D NZF3 vs Lys33-Ub2
Time (min)
0 10 20 30 40 50 60
0.0
-0.2
-0.4
µcal/sec
0
-2
-4
kcal/mole of injectant
K_d = N.D.
0 1 2 3 4
Molar Ratio
E NZF1 M26A vs Lys29-Ub2
Time (min)
0 10 20 30 40 50 60
0.0
-0.2
-0.4
µcal/sec
0
-2
-4
kcal/mole of injectant
K_d = N.D.
0.0 1.0 2.0 3.0 4.0
Molar Ratio

Figure S3. Linkage specificity analysis of TRABID NZF domains, Related to Figure 3.

(**A**) Input proteins (1%) that were coupled to the HaloLink resin in Figure 3B and 3C were visualized on Coomassie-stained SDS-PAGE gel.

(**B-C**) Isothermal titration calorimetry (ITC) measurements for the NZF2 domain of TRABID with K29-Ub2 (B) and K33-Ub2 (C).

(**D**) ITC measurement for NZF3 domain of TRABID with K33-Ub2.

(**E**) ITC measurement for NZF1 M26A mutant of TRABID with K29-Ub2. The K_d value for each measurement is indicated.

Figure S4

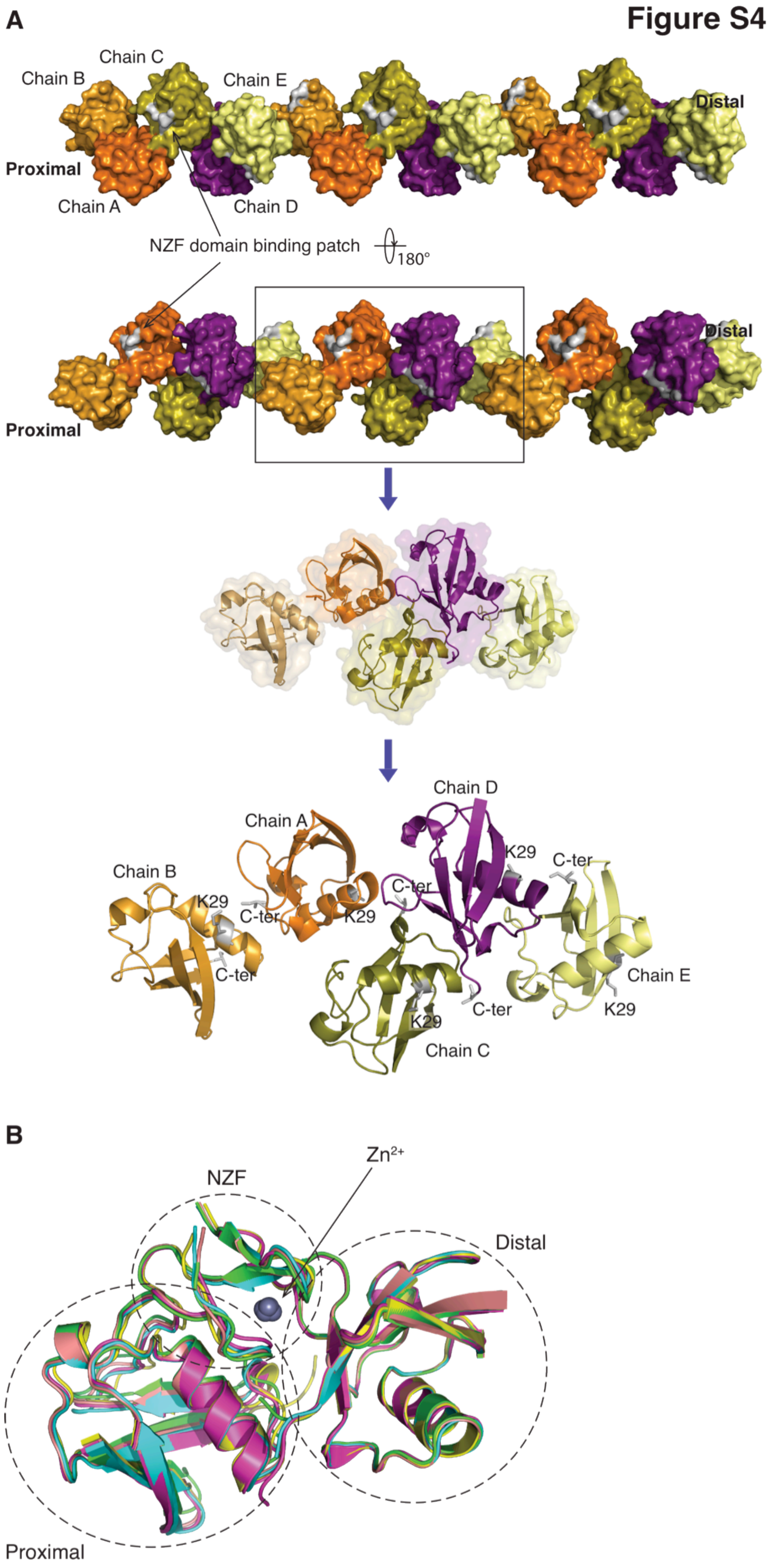
A
Chain C
Chain B
Chain E
Distal
Proximal
Chain A
Chain D
NZF domain binding patch
180°
Distal
Proximal
Chain D
Chain A
K29
C-ter
Chain B
K29
C-ter
K29
C-ter
C-ter
Chain E
C-ter
K29
K29
Chain C
B
Zn2+
NZF
Distal
Proximal

Figure S4. K29-linked chains form a helical filament like structure, Related to Figure 4. (**A**) The asymmetric unit (ASU) of the complex, contains five Ub moieties (chain A, B, C, D, E) and five NZF domains (chain F, G, H, I, J) (top panel). Symmetry related molecules of the ASU are expanded and the orientation of K29 of each Ub moiety and the C-terminal of the adjacent Ub moiety are shown (bottom panel). (**B**) Superposition of all NZF1-K29-diUb complexes within the ASU. The rmsd values range between 0.363 and 0.883 Å.

Figure S5

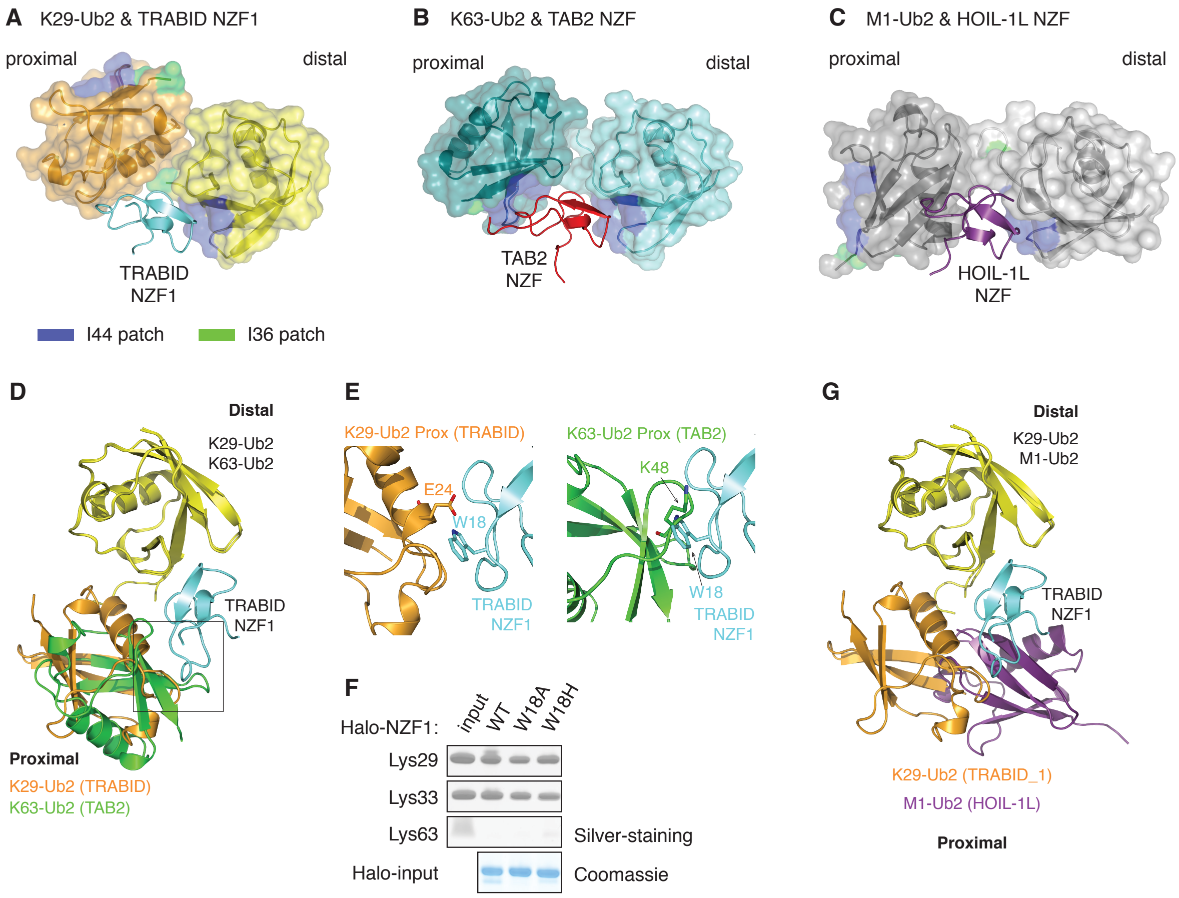

A K29-Ub2 & TRABID NZF1
proximal
distal
TRABID NZF1
I44 patch
I36 patch
B K63-Ub2 & TAB2 NZF
proximal
distal
TAB2 NZF
C M1-Ub2 & HOIL-1L NZF
proximal
distal
HOIL-1L NZF
D
Distal
K29-Ub2
K63-Ub2
TRABID NZF1
Proximal
K29-Ub2 (TRABID)
K63-Ub2 (TAB2)
E
K29-Ub2 Prox (TRABID)
E24
W18
TRABID NZF1
K63-Ub2 Prox (TAB2)
K48
W18
TRABID NZF1
F
Halo-NZF1:
input
WT
W18A
W18H
Lys29
Lys33
Lys63
Silver-staining
Halo-input
Coomassie
G
Distal
K29-Ub2
M1-Ub2
TRABID NZF1
K29-Ub2 (TRABID_1)
M1-Ub2 (HOIL-1L)
Proximal

Figure S5. Two-sided Ub-binding mode of NZF domains, Related to Figure 5.

(**A-C**) A semitransparent surface of diUb and NZF domains in cartoon representative of K29-Ub2 and TRABID NZF1 (A), K63-Ub2 and TAB2 NZF (B), and M1-Ub2 and HOIL-1L NZF (C). Residues comprising I44 patch (I44, L8, H68, and V70) and I36 patch (I36, L71, and L73) are colored blue and green, respectively.

(**D**) Superposition of the distal Ub moieties (yellow) of K29-Ub2-TRABID NZF1 (orange) and K63-Ub2-TAB2 (green). For clarity, only the NZF1 of TRABID is shown (cyan).

(**E**) Zoom-in of the boxed region in (D) was reoriented to show the position of W18 of TRABID NZF1 in proximity of E24 on the proximal Ub of K29-diUb (top) and steric clashes of TRABID NZF1 with the proximal Ub of the superposed K63-diUb (bottom).

(**F**) Mutating W18 residue of NZF1 does not increase binding to K63-Ub2. Halo-tagged NZF1 wild type or mutants were used in pull-down assay with K29-, K33-, and K63-Ub2. The captured chains were visualized by silver-staining. 50% of tetraUb input used in the pull down assay was included as control.

(**G**) Superposition of the distal Ub moieties (yellow) of K29-Ub2-TRABID NZF1 (orange) and Met1-Ub2-HOIL-1L (purple). For clarity, only the NZF1 of TRABID is shown (cyan). PDB ID: 2WWZ, (Kulathu et al., 2009); 3B08, (Sato et al., 2011).

Figure S6

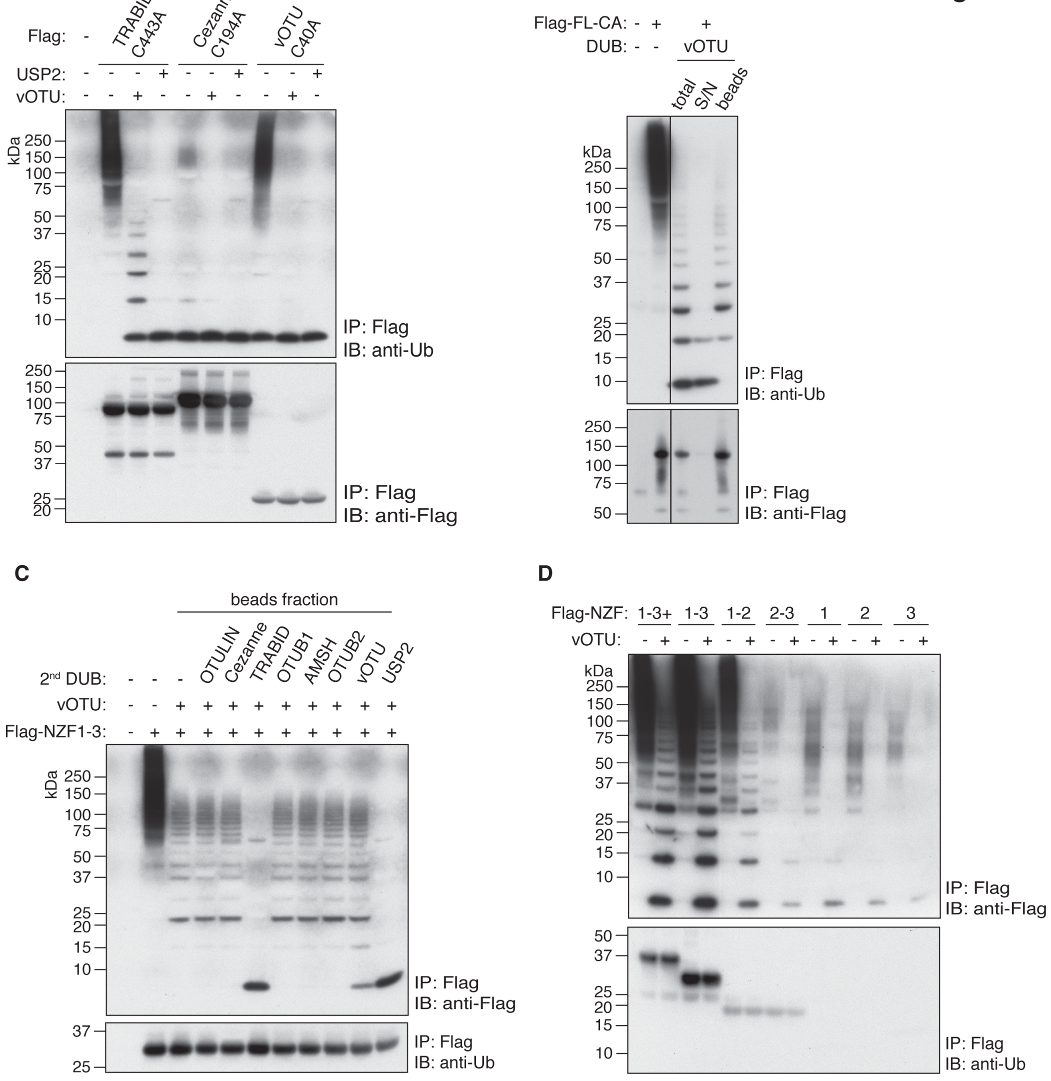
A
Flag:
TRABID C443A
Cezanne C194A
vOTU C40A
USP2:
vOTU:
kDa
250
150
100
75
50
37
25
20
15
10
IP: Flag
IB: anti-Ub
IP: Flag
IB: anti-Flag
B
Flag-FL-CA:
DUB:
vOTU
total
S/N
beads
IP: Flag
IB: anti-Ub
IP: Flag
IB: anti-Flag
C
beads fraction
2nd DUB:
vOTU:
Flag-NZF1-3:
OTULIN
Cezanne
TRABID
OTUB1
AMSH
OTUB2
vOTU
USP2
IP: Flag
IB: anti-Flag
IP: Flag
IB: anti-Ub
D
Flag-NZF:
1-3+
1-3
1-2
2-3
1
2
3
vOTU:
IP: Flag
IB: anti-Flag
IP: Flag
IB: anti-Ub

Figure S6. The tandem NZF1-3 domain of TRABID captures polyUb chains containing K29 linkages from HEK293 cells, Related to Figure 6.

(**A**) Flag-tagged catalytic dead TRABID (C443A), Cezanne (C194A), and vOTU (C40A) were transiently expressed in HEK293 cells. The Flag-fusion proteins were immunoprecipitated and the captured polyUb material was incubated with vOTU to determine if K29 linkages are present. Short chains are released from TRABID isolates but not from vOTU or Cezanne pull-downs.

(**B**) K29 chains are present in the bead fraction bound to TRABID. Flag-tagged full-length catalytic dead TRABID (FL-CA) was transiently expressed in HEK293 cells. The Flag-fusion proteins were immunoprecipitated and the captured polyUb material was incubated with vOTU. The ubiquitin content of the supernatant and bead fraction were analyzed by anti-Ub immunoblotting.

(**C**) Flag-tagged TRABID NZF1-3 was immunoprecipitated from transfected HEK293 cells and incubated with vOTU. The bead fraction containing vOTU resistant chains was assayed for its Ub linkage content by incubation with DUBs that exhibit preference for cleaving different linkages.

(**D**) The indicated Flag-tagged TRABID constructs were transiently expressed in HEK293 cells, immunoprecipitated using Flag-M2 beads, and the captured polyUb material was treated with vOTU. Ectopic TRABID NZF expression was visualized by anti-FLAG immunoblotting.

Figure S7

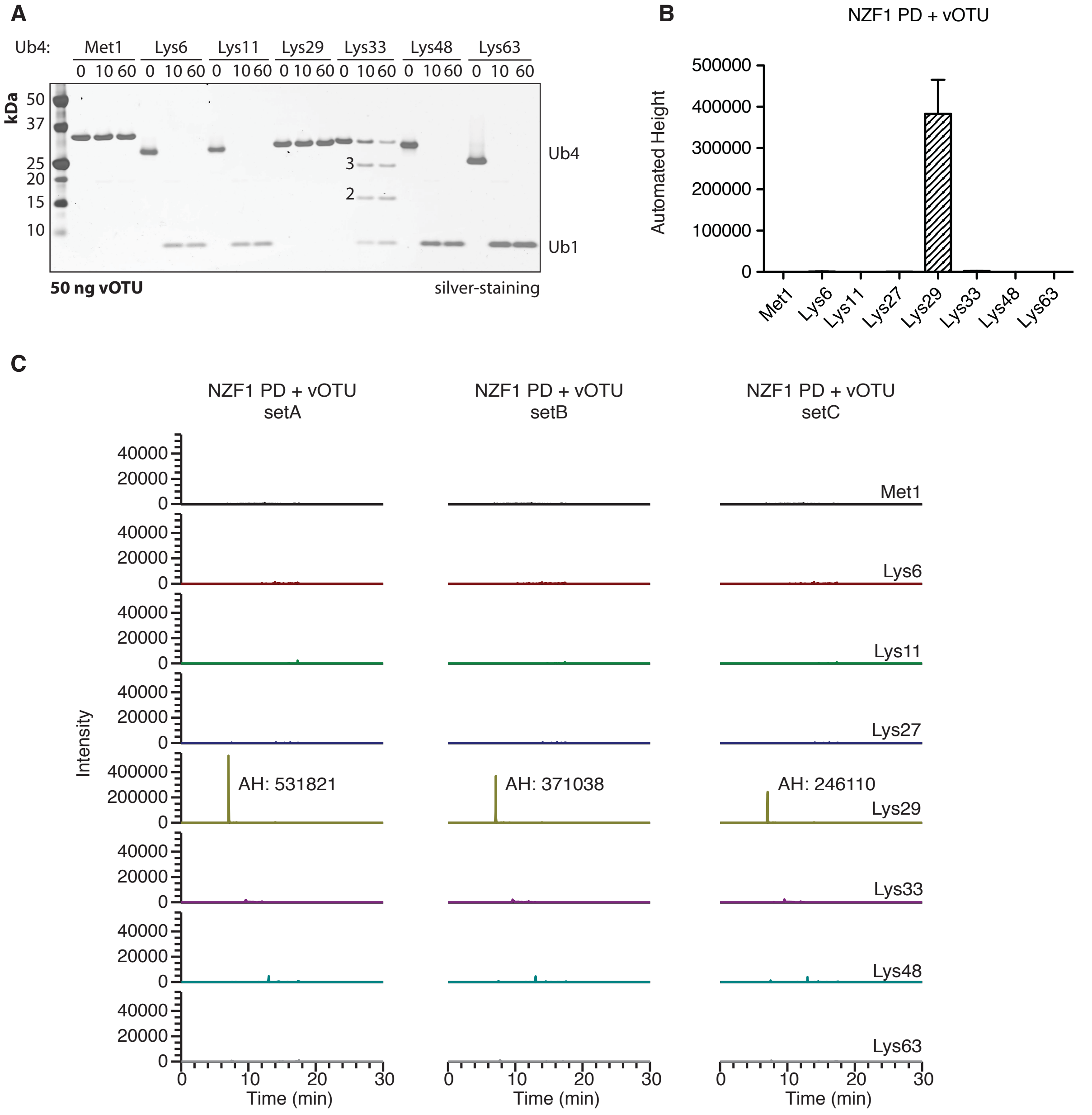
A
Ub4:
Met1
Lys6
Lys11
Lys29
Lys33
Lys48
Lys63
0 10 60
kDa
50
37
25
20
15
10
3
2
Ub4
Ub1
50 ng vOTU
silver-staining
B
NZF1 PD + vOTU
Automated Height
500000
400000
300000
200000
100000
0
Met1
Lys6
Lys11
Lys27
Lys29
Lys33
Lys48
Lys63
C
NZF1 PD + vOTU setA
NZF1 PD + vOTU setB
NZF1 PD + vOTU setC
Intensity
40000
20000
0
400000
200000
AH: 531821
AH: 371038
AH: 246110
Met1
Lys6
Lys11
Lys27
Lys29
Lys33
Lys48
Lys63
0
10
20
30
Time (min)

Figure S7. Verification that the linkage type present in the vOTU-resistant polyUb chains is K29, Related to Figure 6.

(**A**) Deubiquitinase reactions were carried out in 10 µl reaction of 50 ng vOTU and 0.5 µg tetraUb of seven linkages at 30 °C for the indicated time. Reaction was quenched by adding LDS sample buffer and analyzed on silver-stained 4-12% SDS-PAGE gel.

(**B**) PolyUb chains captured from HEK293 cells using Halo-NZF1 were treated with vOTU. The abundance of Ub linkages of the vOTU-resistant chains that were still captured on the resin was analyzed by parallel reaction monitoring (pRM) as in Figure S1D. The error bar represents the standard error of the mean of three measurements.

(**C**) The extracted-ion chromatography of vOTU-resistant chains analyzed by pRM in (B). To highlight the abundance of the K29-linkage over other linkages, the intensity scale for the non-K29 linkages was set 10-fold lower. AH: refers to automated height as determined by the XCalibur software.

Figure S8

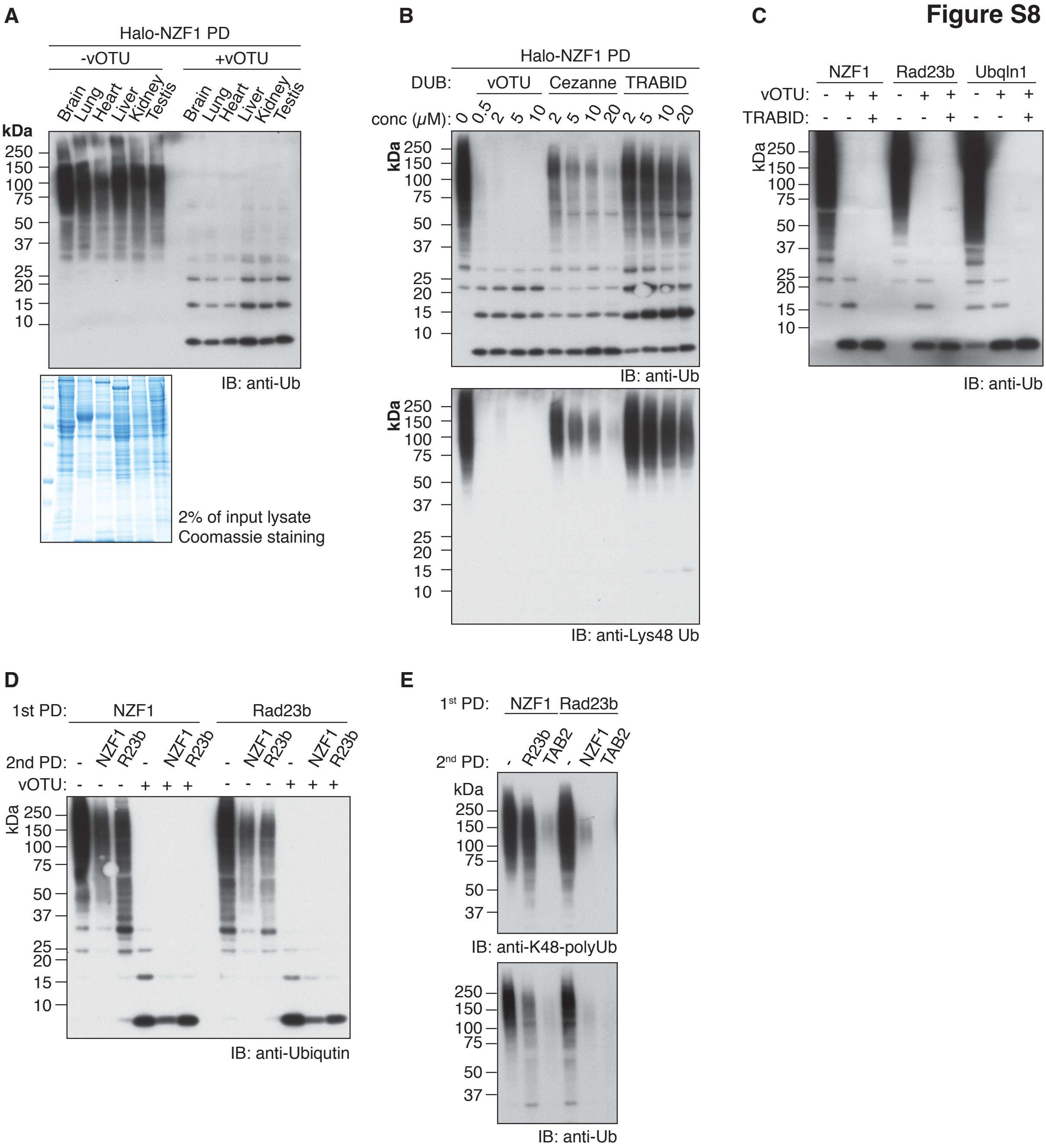
A
Halo-NZF1 PD
-vOTU
+vOTU
Brain Lung Heart Liver Kidney Testis
kDa
250 150 100 75 50 37 25 20 15 10
IB: anti-Ub
2% of input lysate
Coomassie staining
B
Halo-NZF1 PD
DUB: vOTU Cezanne TRABID
conc (μM): 0 0.5 2 5 10 2 5 10 20 2 5 10 20
IB: anti-Ub
IB: anti-Lys48 Ub
C
NZF1 Rad23b Ubqln1
vOTU:
TRABID:
IB: anti-Ub
D
1st PD: NZF1 Rad23b
2nd PD: - NZF1 R23b - NZF1 R23b - NZF1 R23b - NZF1 R23b
vOTU: - - - + + + - - - + + +
IB: anti-Ubiqutin
E
1st PD: NZF1 Rad23b
2nd PD: - R23b TAB2 - NZF1 TAB2
IB: anti-K48-polyUb
IB: anti-Ub

Figure S8. K29-linked chains are present within mixed or branched heterotypic chains, Related to Figure 7.

(**A**) PolyUb chains from the indicated mouse tissues were captured using Halo-TRABID NZF1 and the presence of K29 chains was assayed by treating one half of the samples with vOTU. For a loading control, 2% of input lysate for IP was separated and visualized on Coomassie-stained SDS-PAGE gel.

(**B**) PolyUb chains from HEK293 were captured using Halo-TRABID NZF1 and incubated with an increasing concentration of vOTU, Cezanne, and TRABID for 1 h, 2 h, and 2 h, respectively. The K48-linked polyUb chains were analyzed using anti-K48 Ub linkage.

(**C**) PolyUb materials from HEK293 cells captured by Halo-TRABID NZF1, Halo-Rad23b UBA1-2, and Halo-Ubiquilin1 UBA were treated with vOTU and TRABID as indicated.

(**D**) PolyUb materials from HEK293 cells were captured by Halo-TRABID NZF1 or Halo-Rad23b, and where indicated, the supernatants from the first pull-down were subjected to a second pull-down. The presence of K29 linkages was assayed by incubating one half of the pull-down material with vOTU.

(**E**) As in (D), except the captured polyUb species were not treated with vOTU. The presence of K48 linkages in the pulled-down polyUb material was visualized using anti-K48 polyUb immunoblotting.

Table S1. cDNA constructs used in this study, Related to Figure 1, 3-7, S1, S3, S5-S8.

Protein	Tag	Accession No	Construct Boundaries	Expression system	Plasmid	DU number
UBE3C	GST-cleaved	Q15386	641-1083	insect	pFastbac	45301
vOTU	GST-cleaved	3ZNH_A	1-183	bacterial	pGEX6P	45351
TRABID NZF1	GST-cleaved	Q9UGI0	3-33	bacterial	pGEX6P	23225
TRABID NZF1-3	GST-cleaved, Halo	Q9UGI0	3-178	bacterial	pGEX6P	24214
TRABID NZF1-2	GST-cleaved, Halo	Q9UGI0	3-110	bacterial	pGEX6P	49556
TRABID NZF2-3	GST-cleaved, Halo	Q9UGI0	82-187	bacterial	pGEX6P	24485
TRABID NZF1	GST-cleaved, Halo	Q9UGI0	1-33	bacterial	pGEX6P	24486
TRABID NZF2	GST-cleaved, Halo	Q9UGI0	82-113	bacterial	pGEX6P	24487
TRABID NZF3	GST-cleaved, Halo	Q9UGI0	139-187	bacterial	pGEX6P	24488
Npl4 NZF	GST-cleaved, Halo	Q8TAT6	575-608	bacterial	pGEX6P	49635
Ubiquilin1 UBA	GST-cleaved, Halo	Q9UMX0	539-587	bacterial	pGEX6P	49634
Rad23b UBA1-2	GST-cleaved, Halo	P54727	186-407	bacterial	pGEX6P	49626
TAB2	GST-cleaved, Halo	Q9NYJ8	663-693	bacterial	pGEX6P	49635
TRABID FL-WT	3xFlag	Q9UGI0	full length	mammalian	pcDNA5	49067
TRABID FL-C443A	3xFlag	Q9UGI0	full length	mammalian	pcDNA5	49089
TRABID NZF1-3	3xFlag	Q9UGI0	1-187	mammalian	pcDNA5	24389
Cezanne C194A	3xFlag	Q6GQQ9	full length	mammalian	pcDNA5	49193
vOTU C40A	3xFlag	3ZNH_A	1-183	mammalian	pcDNA5	49177
TRABID NZF1-3+	3xFlag	Q9UGI0	1-245	mammalian	pcDNA5	49140
TRABID NZF1-2	3xFlag	Q9UGI0	1-113	mammalian	pcDNA5	24391
TRABID NZF2-3	3xFlag	Q9UGI0	82-187	mammalian	pcDNA5	24390
TRABID NZF1	3xFlag	Q9UGI0	1-33	mammalian	pcDNA5	24394
TRABID NZF2	3xFlag	Q9UGI0	82-113	mammalian	pcDNA5	24393
TRABID NZF3	3xFlag	Q9UGI0	139-187	mammalian	pcDNA5	24392

Table S2. Parameter used in the parallel Reaction Monitoring (pRM) analysis, Related to Figure S1 and S7.

Linkage	Peptide Sequence	Charge	Retention Time	Precursor (m/z)	Daughter masses used for quantification
M1	M(gg)QIFVK	2+	8.7	440.34	y_3, 393.25; y_4, 506.33; y_5, 634.39; b_7, 733.37
K6	MQIFVK(gg)TLTGK	2+	13.6	690.50	y_6, 761.45; y_7, 860.50; y_8, 1007.59; y_9, 1120.67
K11	TLTGK(gg)TITLEVEPSDTIENVK	3+	14.6	801.84	y_8, 905.46; y_9 1002.51; y_{10}, 1131.55
K27	TITLEVEPSDTIENVK(gg)AK	2+	13.9	1051.56	y_8, 1016.57; y_{11}, 1315.68; y_{12}, 1444.73; y_{13}, 1543.79
K29	AK(gg)IQDK	2+	6.6	408.80	b_3, 427.26; y_4, 503.30; b_5, 670.35; y_5, 745.40
K33	IQDK(gg)EGIPPDQQR	3+	9.3	546.61	y_6^{2+}, 370.90; y_6, 740.30; b_7, 898.30
K48	LIFAGK(gg)QLEDGR	2+	12.7	731.36	y_4, 476.40; y_5, 589.45; y_6, 717.20
K63	TLSDYNIQK(gg)ESTLHLVLR	2+	14.5	1122.67	y_5, 637.41; y_8,938.58; y_9, 1067.62

Table S3. Summary of interactions in K29-linked diUb – TRABID NZF1 complex, Related to Figure 4.

Distal Ub with NZF

Ub^{dist}	NZF	Interaction
Leu8	Tyr12	Hydrophobic
Ile44	Tyr15	Hydophobic
Ile44	Met26	Hydophobic
Val70	Met26	Hydophobic
Gly47	Glu16	Hydophobic
Gln49	Met26	Hydrogen

Proximal Ub with NZF

Ub^{prox}	NZF	Interaction
Ala28	Met26	Hydophobic
Glu24	Tyr15	Hydrogen
Glu24	Thr25	Hydrogen

Distal Ub with Proximal Ub

Ub^{dist}	Ub^{prox}	Interaction
Arg42	Asp32	Ionic, Hydrogen
Gln49	Gln31	Hydrogen
Leu73	Asp32	Hydrogen
Arg72	Asp32	Ionic

Table S4. K_d values of TRABID NZF domain binding to polyubiquitin, Related to Figure 3 and S3.

	K29-Ub2	K33-Ub2
NZF1	3.0	4.2
NZF2	9.4	6.0
NZF3	-	N.D.
NZF1 M26A	N.D.	-

Values (in μM) were determined by ITC.
ND, no detectable binding

Extended Experimental Procedures

Plasmids and antibodies. All cDNA constructs for bacterial, insect, and mammalian expression system were generated by the DNA cloning team, Division of Signal transduction Therapy, Medical Research Council Protein Phosphorylation and Ubiquitylation Unit, University of Dundee, United Kingdom (**Table S1**). Recombinant proteins and plasmids generated for the present study are available on our reagents website (https://mrcppureagents.dundee.ac.uk/). Anti-ubiquitin to detect *in vitro* ubiquitylation and polyUb chains from cells were purchased from SIGMA (U5379) and DAKO (Z0458), respectively. Anti-Flag was from SIGMA (F3165) and anti-HA was from Cell Signaling Technology (#3724).

Protein expression and purification. For bacterial expression, recombinant GST-fusion proteins were expressed in BL21 (DE3) *E. coli* cells. Cultures were grown in 2xTY media to OD_{600} of 0.6-0.8 and the protein expression was induced by adding 300 μM IPTG and further incubation at 16 °C overnight. Medium was supplemented with 200 μM $ZnCl_2$ for expression of NZF domains. Cells were lysed by sonication in GST-Lysis Buffer (50 mM Tris-HCl (pH 7.5), 300 mM NaCl, 10% glycerol, 0.075% 2-mercaptoethanol, 1 mM benzamidine, 1 mM AEBSF, and complete protease inhibitor cocktail (Roche)). Bacterial lysate was clarified by centrifugation and incubated subsequently with Glutathione Sepharose 4B resin (GE Healthcare) for 2 h at 4 °C. Resins were washed extensively with high salt buffer (250 mM Tris (pH 7.5), 500 mM NaCl, and 5 mM DTT) and low salt buffer (25 mM Tris (pH 7.5), 150 mM NaCl, 10% glycerol, and 1 mM DTT). Recombinant proteins were eluted from the resin by cleaving the GST-tag using GST-tagged C3 protease.

For insect cell protein expression, recombinant GST-fusion UBE3C (641-1083) was expressed in Sf21 cells using Bac-to-Bac baculovirus expression system (Invitrogen). Sf21 cells cultured at 27 °C in Insect Xpress medium (Lonza) supplemented with Antibiotic-Antimycotic (Invitrogen) were infected with P1 virus stocks and harvested 60 h later. Cells were lysed in Lysis Buffer (50 mM Tris-HCl (pH 7.5), 5% glycerol, 0.1 M EDTA, 0.1 mM EGTA, 1 mM DTT, 1 mM Pefabloc, and 20 μg/ml Leupeptin) using Dounce homogenizer, then centrifuged to remove insoluble material. Total concentration of 250 mM NaCl was added to

the lysate prior to a one-hour incubation with Glutathione agarose (Expedeon). Resins were washed in wash buffer (50 mM Tris (pH 7.5), 500 mM NaCl, 5% glycerol, 1 mM DTT) and then cleavage buffer (50 mM Tris-HCl (pH 7.5), 150 mM NaCl, 1 mM DTT). Recombinant proteins were eluted from the resin by cleaving the GST-tag off using C3 protease.

Ubiquitylation assays. Analytical assays were carried out in 20-µl reactions at 30 °C containing 250 nM UBE1, 2.25 µM E2 (UBE2D1, D2, D3, or L3), 1.56 µM UBE3C, 57 µM Ub, 10 mM ATP, 50 mM Tris-HCl (pH 7.5), 10 mM $MgCl_2$, and 0.6 mM DTT. After 3 h, 2 µM vOTU was added and the reaction was continued for 2 h. The reaction was stopped by addition of 10 µl 4 x LDS sample buffer (Invitrogen), resolved by SDS-PAGE on 4-12% gradient gels (Invitrogen) and subjected to western blot analysis using rabbit polyclonal anti-Ub antibody (SIGMA).

Purification of polyubiquitin chains. Following large-scale assembly of K29-linked polyUb chain, enzymes used in the reaction were precipitated by diluting the Ub chains in a total volume of 50 ml of 50 mM sodium acetate (pH 4.5). After at least 3 h incubation at 4 °C, the solution was passed through a 0.22-µM syringe filter and the K29-linked diUb, triUb, tetraUb, and pentaUb were purified by cation exchange using a Resource S 6 ml column (GE Healthcare), equilibrated in 50 mM sodium acetate (pH 4.5), and eluted in a gradient with elution buffer (50 mM sodium acetate (pH 4.5), 1 M NaCl). For crystallization, peak fractions containing K29-linked diUb were concentrated to 12 mg/ml.

Analysis of polyubiquitin linkages by parallel Reaction Monitoring (pRM) using LC-MS-MS. PolyUb chains that had been previously digested with trypsin were analyzed on an LTQ-Velos mass spectrometer (Thermo) fitted with a Dionex RSLC HPLC system and an Easy-Spray Source (Thermo). Standard diUb chains were purchased from Boston Biochemicals and a synthetic peptide AK(GG)IQDK representing the tryptic Ub K29 linkage was purchased from Pepceuticals (Nottingham, UK). Digests (prepared in 0.1% TFA/water) were loaded onto a 20 x 0.1 mm nanotrap column (Thermo) equilibrated in 0.1% TFA/water (10 µl/min), washed with 10 µl of the same buffer, and then separated on a 150 x 0.075 mm PepMap C18, 3 µm Easy-Spray column (Thermo)

equilibrated with 2% acetonitrile/ 0.1% formic acid/ water at 300 nl/min. It was critical that the samples were loaded and washed in TFA buffers, as the trap column in the presence of formic acid did not retain the tryptic peptide containing the K29 linkage. Peptides were separated at the same flow rate using a discontinuous gradient of buffer B (80% acetonitrile/ 0.1% formic acid/ water) as follows: 0-14 min = 1-30% B, 14-15 min = 30-80% B, 15-20 min = 80% B. LC-MS data was acquired in Data Independent mode with 1 full scan (m/z 350-1800) followed by 8 product ion scans as described below. The voltage applied to the Easy-Spray column was 1.9 kV, the isolation width was set to 1 Da, normalised collision energy was 35 and the activation time was 10 ms. Xcalibur software (Thermo) was used to process the data, with the ion current for the daughter ions being summed for each precursor mass analyzed (**Table S2**). The resultant summed intensities provide the y-axis values for **Figure S1D** and **Figure S7C**. This was a much cleaner and selective analysis method rather than using the extracted ion current for the precursor mass for each ubiquitin chain peptide.

Crystallization and structure determination. Purified K29-linked diUb chains were crystallized at 12 mg/ml in mother liquor containing 100 mM Bis-Tris propane (pH 6.5), 200 mM sodium iodide, 20% PEG3350, 5% ethylene glycol and 5 mM sodium malonate at 20 °C. Further, seeding technique was used to obtain diffraction quality crystals. The single crystals obtained were cryo-protected in mother liquor containing 20% glycerol and 20% PEG400 before freezing in liquid nitrogen.

For crystallization of the complex, TRABID NZF1 and K29 diUb were mixed together in molar ratio of 1:1 and incubated for 3 hours at 4 °C before concentration using Millipore concentrator (MW cut off 3kDa). Crystallization screening was set up using protein complex concentrated to 18 mg/ml and crystallization trays incubated at 12 °C. The complex crystallized in mother liquor containing 100 mM MES (pH 6.5), 200 mM potassium iodide, and 25% PEG4000. The crystals grew to maximum size in one week and were cryo-protected in 100 mM MES (pH 6.5), 10% PEG20000, and 35% PEG400 before vitrification in liquid nitrogen.

Diffraction data for K29 diUb and the NZF1-K29 diUb complex were collected at ESRF beam line ID23-1 and Diamond beam line I04, respectively. All data were processed using XDS (Kabsch, 2010) and scaled using SCALA (Evans, 2006). The structures were solved by molecular replacement using the structures of ubiquitin (1UBQ (Vijay-Kumar et al., 1987)) and the NZF domain of TAB2 (2WWZ (Kulathu et al., 2009)) as search models in Phaser (McCoy et al., 2005). Iterative rounds of refinement was done using Phenix (Adams et al., 2002) and REFMAC (Murshudov et al., 1997) with model building in Coot (Emsley and Cowtan, 2004). Simulated annealing and rigid body refinement was used in the initial stages to remove model bias. Final re-refinement for K29 diUb was done using PDB_REDO (Joosten et al., 2014). Both structures were refined to the final statistics as shown in **Table 1**.

Isothermal Titration Calorimetry. ITC titration were performed on a MicroCal™ iTC$_{200}$ at 25 °C in ITC buffer (50 mM HEPES (pH 7.5), 150 mM NaCl, 250 µM TCEP). Each titration used 25 × 1.5 µl injections. For all titrations, the syringe contained the NZF proteins and the cell contained diUb. Concentration of 300 µM and 20 µM was used for titrations of NZF1 and diUb, respectively. For titration of NZF2 and diUb, concentration of 550 µM and 30 µM was used, respectively. For titration of NZF3 (800 µM) and K33-Ub2 (40 µM), and NZF1 M26A (550 µM) and K29-Ub2 (30 µM), higher concentration of proteins was used to increase the ITC signal.

Assembly and purification of tetraubiquitin chains. K6-linked chains were enzymatically assembled using wild type Ub, UBE2L3 and NleL as described before (Hospenthal et al., 2013). K11-linked tetraUb was purified from ubiquitylation products generated by UBE2S (1-192) and contaminating K63 chains were removed by incubation with AMSH (Bremm et al., 2010). K48- and K63-linked polyUb chains were assembled as described using UBE2R1 and UBE2N/UBE2V1 respectively (Kulathu et al., 2009), while HOIP was used for assembly for M1-linked chains (Stieglitz et al., 2012). K33-linked polyUb chains were assembled as described (Kristariyanto, et al., manuscript submitted). K29-linked polyUb chains were assembled as described above. For each linkage type, polyUb of defined lengths were separated by cation exchange chromatography

and the fractions containing tetraUb were concentrated and dialyzed into 50mM Tris, pH7.5 buffer.

Cell culture, transfection, and cell lysis. HEK293 cells were maintained in DMEM supplemented with 10% (v/v) FBS, 2 mM L-glutamine and antibiotics (100 units/ml penicillin, 0.1 mg/ml streptomycin) and were cultured at 37 °C in a 10% CO2 humidified atmosphere. All 3xFlag-tagged proteins in mammalian expressing vector (**Table S1**) were introduced to HEK293 cells using polyethylenimine (PEI) (Bioscience). Once the cells were confluent, they were harvested in PBS, pelleted, snap frozen, and stored at -80 °C. The cells pellet was thawed and incubated in Lysis Buffer (20 mM HEPES (pH 7.5), 110 mM potassium acetate, 2 mM magnesium acetate, 1 mM EGTA, 1 mM sodium ortho-vanadate, 1 mM NaF, 0.1% NP-40, 1 mM ABSF, 25 mM iodoacetamide, 0.02% benzonase (SIGMA), protease inhibitor cocktail) for 30 min at 4 °C in the dark. Cells were lysed by two times freeze-and-thawing cycle and a final concentration of 150 mM NaCl and 5% glycerol was added. Cell lysates were clarified by centrifugation at 14000 x g for 20 min at 4 °C, supernatants were collected and protein concentrations were determined by the Bradford procedure.

Pull-down assays and deubiquitinase treatments. To isolate transiently expressed Flag-tagged proteins and the interacting polyUb chains from HEK293 cells, 1 mg of the transfected cell lysates was incubated with 10 µl of Flag-M2 resin (SIGMA) for 2 h at 4 °C. To isolate polyUb chains from HEK293 cells using bacterially expressed Halo-tagged UBDs, 1 mg of the cell lysates was incubated with 10 µl Halo-UBD resins for 2 h at 4 °C. The polyUb chains captured by 3xFlag- and Halo-tagged proteins were washed three times with Lysis Buffer containing 150 mM NaCl, and once with DUB buffer (50 mM Tris-HCl (pH 7.5), 150 mM NaCl, and 5 mM DTT). Following the last wash, the beads were resuspended in 2x resin volume of 2 µM vOTU for 1 h at 30 °C. When subsequent DUB assays were carried out after the first incubation with vOTU, the beads were first washed twice with DUB buffer and then incubated with 5 µM DUBs (OTULIN, Cezanne, TRABID, OTUB1, AMSH, OTUB2, vOTU, and USP2) in 2x resin volume for 1 h at 30 °C. Reaction was quenched by adding reducing LDS buffer (Invitrogen), and the polyUb chains were analyzed in by immunoblotting against anti-Ub (DAKO)

or anti-K48 Ub linkage (CST). The immunoprecipitated Flag-tagged proteins were visualized by anti-FLAG (SIGMA).

For the tetraUb pull-down assays, 10 µl of the Halo-UBDs resins were incubated with 1 µg tetraUb chains of of the indicated linkages (M1, K6, K11, K29, K33, K48, and K63 tetraUb) in 500 µl pull-down buffer (50 mMTris-HCl (pH 7.5), 150 mM NaCl, 0.1% NP-40, 5 mM DTT, 0.5 mg/ml BSA) for 2 h at 4 °C. Beads were washed three times in Wash Buffer (50 mM Tris-HCl (pH 7.5), 250 mM NaCl, 0.2% NP-40, and 5 mM DTT). Captured tetraUb chains were separated on 4-12% SDS-PAGE gel and visualized using silver-staining kit (Thermo Scientific).

Supplemental References

Adams, P.D., Grosse Kunstleve, R.W., Hung, L.-W., Ioerger, T.R., McCoy, A.J., Moriarty, N.W., Read, R.J., Sacchettini, J.C., Sauter, N.K., and Terwilliger, T.C. (2002). PHENIX: building new software for automated crystallographic structure determination. Acta Crystallogr. D Biol. Crystallogr. *58*, 1948–1954.

Bremm, A., Freund, S.M.V., and Komander, D. (2010). Lys11-linked ubiquitin chains adopt compact conformations and are preferentially hydrolyzed by the deubiquitinase Cezanne. Nat. Struct. Mol. Biol. *17*, 939–947.

Emsley, P., and Cowtan, K. (2004). Coot: model-building tools for molecular graphics. Acta Crystallogr. D Biol. Crystallogr. *60*, 2126–2132.

Evans, P. (2006). Scaling and assessment of data quality. Acta Crystallogr. D Biol. Crystallogr. *62*, 72–82.

Hospenthal, M.K., Freund, S.M.V., and Komander, D. (2013). Assembly, analysis and architecture of atypical ubiquitin chains. Nat. Struct. Mol. Biol. *20*, 555–565.

Joosten, R.P., Long, F., Murshudov, G.N., and Perrakis, A. (2014). The PDB_REDO server for macromolecular structure model optimization. IUCrJ *1(Pt 4)*, 213-220.

Kabsch, W. (2010). XDS. Acta Crystallogr. D Biol. Crystallogr. *66*, 125–132.

Kulathu, Y., Akutsu, M., Bremm, A., Hofmann, K., and Komander, D. (2009). Two-sided ubiquitin binding explains specificity of the TAB2 NZF domain. Nat. Struct. Mol. Biol. *16*, 1328–1330.

McCoy, A.J., Grosse Kunstleve, R.W., Storoni, L.C., and Read, R.J. (2005). Likelihood-enhanced fast translation functions. Acta Crystallogr. D Biol. Crystallogr. *61*, 458–464.

Murshudov, G.N., Vagin, A.A., and Dodson, E.J. (1997). Refinement of macromolecular structures by the maximum-likelihood method. Acta Crystallogr. D Biol. Crystallogr. *53*, 240–255.

Sato, Y., Fujita, H., Yoshikawa, A., Yamashita, M., Yamagata, A., Kaiser, S.E., Iwai, K., and Fukai, S. (2011). Specific recognition of linear ubiquitin chains by the Npl4 zinc finger (NZF) domain of the HOIL-1L subunit of the linear ubiquitin chain assembly complex. Proc. Natl. Acad. Sci. USA *108*, 20520–20525.

Stieglitz, B., Morris Davies, A.C., Koliopoulos, M.G., Christodoulou, E., and Rittinger, K. (2012). LUBAC synthesizes linear ubiquitin chains via a thioester intermediate. EMBO Rep. *13*, 840–846.

Vijay-Kumar, S., Bugg, C.E., and Cook, W.J. (1987). Structure of ubiquitin refined at 1.8 A resolution. J. Mol. Biol. *194*, 531–544.
