# Supplementary material for: K29-Selective Ubiquitin Binding Domain Reveals Structural Basis of Specificity and Heterotypic Nature of K29 Polyubiquitin
Source: Mol Cell. 2015 Apr 2;58(1):83–94. doi: 10.1016/j.molcel.2015.01.041 (PMC4386640; doi:10.1016/j.molcel.2015.01.041)
Supplement: Document S2. Article plus Supplemental Information [file mmc2.pdf]

Molecular Cell

Article

K29-Selective Ubiquitin Binding Domain Reveals Structural Basis of Specificity and Heterotypic Nature of K29 Polyubiquitin

Graphical Abstract

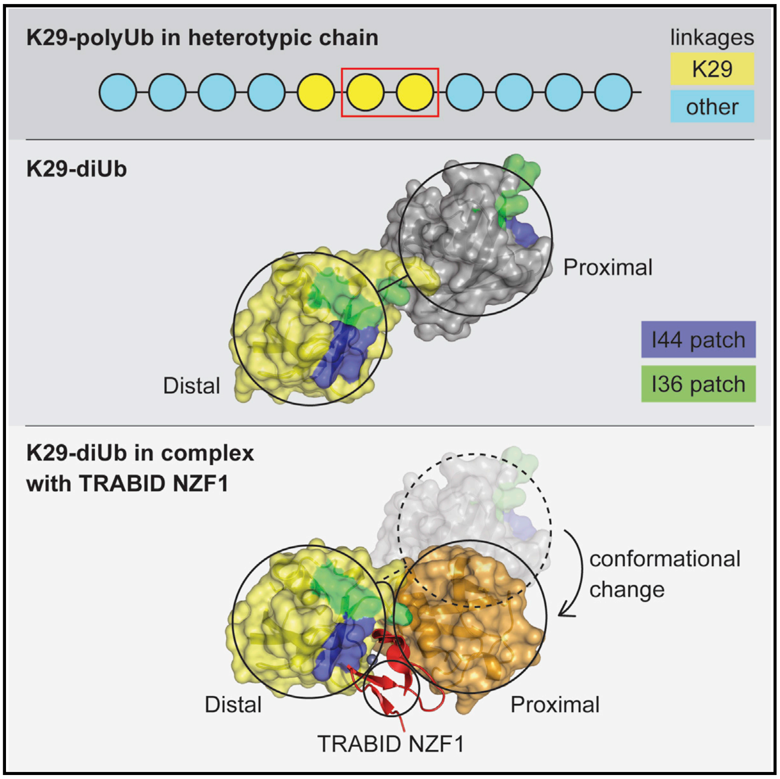

Authors

Yosua Adi Kristariyanto,
Syed Arif Abdul Rehman, ...,
Rachel Toth, Yogesh Kulathu

Correspondence

ykulathu@dundee.ac.uk

In Brief

Kristariyanto et al. find that K29-linked ubiquitin chains are present within ubiquitin chains containing other linkage types. They describe a method to assemble K29 chains, and they characterize a protein domain that selectively binds to these chains.

Highlights

- Large-scale enzymatic assembly and purification of K29-linked polyubiquitin chains
- K29 diubiquitin adopts extended conformation in crystal structure
- Crystal structure of K29 diubiquitin in complex with selective binding domain
- Presence of K29 chains within mixed/branched chains containing other linkages

Accession Numbers

4S22
4S1Z

Kristariyanto et al., 2015, Molecular Cell *58*, 83–94
April 2, 2015 ©2015 The Authors
http://dx.doi.org/10.1016/j.molcel.2015.01.041

Molecular Cell
Article

CellPress

K29-Selective Ubiquitin Binding Domain Reveals Structural Basis of Specificity and Heterotypic Nature of K29 Polyubiquitin

Yosua Adi Kristariyanto,[1] Syed Arif Abdul Rehman,[1] David G. Campbell,[1] Nicholas A. Morrice,[1,2] Clare Johnson,[1] Rachel Toth,[1] and Yogesh Kulathu[1,*]
[1]MRC Protein Phosphorylation and Ubiquitylation Unit, College of Life Sciences, University of Dundee, Dow Street, Dundee DD1 5EH, UK
[2]Present address: AB Sciex UK Limited, Warrington, Cheshire WA1 1RX, UK
*Correspondence: ykulathu@dundee.ac.uk
http://dx.doi.org/10.1016/j.molcel.2015.01.041
This is an open access article under the CC BY license (http://creativecommons.org/licenses/by/4.0/).

SUMMARY

Polyubiquitin chains regulate diverse cellular processes through the ability of ubiquitin to form chains of eight different linkage types. Although detected in yeast and mammals, little is known about K29-linked polyubiquitin. Here we report the generation of K29 chains in vitro using a ubiquitin chain-editing complex consisting of the HECT E3 ligase UBE3C and the deubiquitinase vOTU. We determined the crystal structure of K29-linked diubiquitin, which adopts an extended conformation with the hydrophobic patches on both ubiquitin moieties exposed and available for binding. Indeed, the crystal structure of the NZF1 domain of TRABID in complex with K29 chains reveals a binding mode that involves the hydrophobic patch on only one of the ubiquitin moieties and exploits the flexibility of K29 chains to achieve linkage selective binding. Further, we establish methods to study K29-linked polyubiquitin and find that K29 linkages exist in cells within mixed or branched chains containing other linkages.

INTRODUCTION

Protein ubiquitylation is a reversible posttranslational modification that regulates the activity, function, and fate of modified proteins and is fundamental to diverse biological processes (Komander and Rape, 2012). Topologically distinct ubiquitin (Ub) signals are produced when proteins are monoubiquitylated at one or multiple sites or polyubiquitylated by modification with Ub polymers (Haglund and Dikic, 2005). Homotypic Ub polymers of eight different linkage types can be assembled in which the C terminus of a distal Ub is attached to one of the seven lysine residues (K6, K11, K27, K29, K33, K48, and K63) or to the N-terminal M1 of the proximal Ub. In addition to homotypic chains, heterotypic polyUb of complex topology involving mixed (alternating linkages) or branched (single Ub within a chain modified at two or more sites) types can be formed and have roles in endocytosis, immune signaling, and protein degradation (Boname et al., 2010; Emmerich et al., 2013; Meyer and Rape, 2014).

Differently linked polyUb chains act as functionally distinct signals, and this is mediated in part by Ub binding domains (UBDs) that selectively bind and decode different polyUb modifications (Hurley et al., 2006; Husnjak and Dikic, 2012). UBDs and enzymes of the Ub system exploit the unique conformational and dynamic properties of differently linked polyUb types to achieve linkage selectivity. Structural analyses of five linkage types (K6, K11, K48, K63, and M1) reveal that Ub chains adopt distinct conformations that can be broadly classified as "open" or "compact." In the compact conformation adopted by K6-, K11-, and K48-linked diUb, an intermolecular interface is present between the distal and proximal Ub, whereas in the extended conformation of M1- and K63-linked polyUb, the linkage is the only point of contact between the two Ub molecules (Datta et al., 2009; Komander et al., 2009). However, polyUb is flexible, as compact conformations have been observed for K63 and M1 linkages (Datta et al., 2009; Rohaim et al., 2012).

Proteomic analyses have detected all eight Ub linkages in cells and their relative abundance varies between different cell types and organisms (Dammer et al., 2011; Peng et al., 2003; Ziv et al., 2011). The abundance of particular linkage types increases in response to a specific stimulus as has been observed for M1-, K11-, and K63-linked polyUb (Behrends and Harper, 2011). K48-linked Ub chains, the most abundant linkage type detected in resting cells, function as a signal for proteasomal degradation, while K63- and M1-linked chains have non-proteolytic roles in DNA damage response and immune signaling pathways (Behrends and Harper, 2011; Chau et al., 1989; Spence et al., 1995; Wang et al., 2001). In contrast, the cellular roles of polyUb linked via K6, K27, K29, and K33 (also referred to as atypical polyUb), have yet to be elucidated (Kulathu and Komander, 2012).

Of the atypical linkages, K29 polyUb is the most abundant in resting mammalian cells (Dammer et al., 2011). While the cellular function of this linkage type is unclear, the amount of K29 polyUb increases following proteasomal inhibition. In yeast, K29-linked polyUb has been linked to the Ub-fusion-degradation pathway, where it mediates substrate turnover (Johnson et al., 1995; Koegl et al., 1999). Several HECT E3 ligases, including ITCH, UBR5, and UBE3C, have been suggested to make K29 linkages in cells

CrossMark

(Chastagner et al., 2006; Hay-Koren et al., 2011; You and Pickart, 2001). Recently, the deubiquitinase (DUB) TRABID was identified as having a preference for hydrolyzing K29 and K33 linkages (Licchesi et al., 2012; Virdee et al., 2010). TRABID was discovered as a positive regulator of Wnt signaling and has three tandem NPL4 zinc fingers (NZFs) that are thought to have a preference for binding to K63- and M1-linked polyUb (Komander et al., 2009; Tran et al., 2008).

Research into understanding roles for atypical chains is hampered by the lack of tools and the inability to assemble polyUb chains of different lengths on a large-scale. Although chemical biology approaches can be used to make diUb of all linkage types (Kumar et al., 2010; El Oualid et al., 2010; Virdee et al., 2010), there are no known methods for the large-scale assembly of K27, K29, and K33 linkages using enzymatic means. Further, UBDs that bind specifically to polyUb of a particular linkage type are very useful as polyUb sensors to probe the cellular roles of that linkage (van Wijk et al., 2012). However, no such linkage-selective UBDs have been characterized for atypical chains.

Here we establish a Ub chain-editing complex consisting of an E3 ligase and DUB combination for the large-scale assembly of K29-linked polyUb. This enabled us to determine the structure of K29-linked diUb, which reveals an open conformation. Furthermore, we identify and structurally characterize the first NZF domain (NZF1) of the DUB TRABID as being a highly selective binder of K29- and K33-linked polyUb. Finally, using these selective NZFs, we demonstrate that K29-linked chains feature in mixed or branched chains in cells. Interestingly, short K29-linked chains (diUb to tetraUb) are present in long complex polyUb mixtures suggesting that these heterotypic chains may have distinct properties with specialized function.

RESULTS

Assembly of K29-Linked PolyUb

PolyUb of several linkage types, including K11, K48, and K63, can be assembled in vitro by E2 enzymes (Bremm et al., 2010; Pickart and Raasi, 2005; Ye and Rape, 2009). In addition, HECT-family ligases have been used to catalyze assembly of K6-linked polyUb (Hospenthal et al., 2013). UBE3C, a relatively uncharacterized HECT E3 ligase, has been reported to mainly assemble K29 and K48 linkages (You and Pickart, 2001). In vitro, UBE3C generates small amounts of free diUb and triUb that is rapidly converted to high-molecular weight polyUb, and is mostly autoubiquitylated UBE3C (Figure S1A). Autoubiquitylation of UBE3C occurs in *trans*, as the catalytic dead UBE3C C1051A can be ubiquitylated by wild-type UBE3C (Figure S1C).

In order to obtain free K29 polyUb chains, a DUB that catalyzes removal of contaminating linkages and releases free chains from autoubiquitylated UBE3C is required (Figure 1A). The viral DUB vOTU has recently been shown to catalyze cleavage of all linkages except M1, K27, and K29 (Ritorto et al., 2014). Thus we hypothesized that vOTU may release free polyUb chains from autoubiquitylated UBE3C, thereby enhancing the yield of free chains. Indeed, inclusion of vOTU in the assembly reactions results in unanchored polyUb chains (Figure 1B). We then performed a linkage type analysis by using Ub mutants containing Lys to Arg substitutions. Free polyUb chains that were assembled in the presence of vOTU were not impaired with K6R, K11R, K33R, K48R, or K63R mutants (Figure 1C). By contrast, formation of free polyUb chains was significantly reduced in the presence of Ub K29R, demonstrating that the enzymes UBE3C, UBE2D3, and vOTU can together function as a Ub chain-editing complex for the specific assembly of K29 linkages (Figure 1C). In order to confirm the linkage type of the chains formed, we incubated the chains assembled by this complex with the DUB TRABID that has known specificity for hydrolyzing K29 and K33 linkages. Indeed, chains treated with TRABID were reduced to monoUb, while the M1-specific DUB OTULIN did not hydrolyze these chains (Figure S1B). When the K29only mutant of Ub, where all Lys residues but K29 are mutated to Arg, was used in the assembly reaction, long polyUb chains are assembled and these were hydrolyzed down to monoUb by TRABID (Figure 1D). Using parallel reaction monitoring (pRM) liquid chromatography tandem mass spectrometry (LC-MS/MS) analysis of tryptic fragments (Peterson et al., 2012), we further verified that the Ub chains produced using the described method only contained K29 linkages, and other linkages were not detected (Figure S1D). By scaling up the assembly reactions using the chain-editing complex, we were able to generate and purify K29-linked polyUb chains on a large scale and chains of defined lengths could be separated by cation-exchange chromatography (Figures 1E and 1F). Taken together, these data reveal a robust reproducible method for generating milligram quantities of K29 polyUb.

Crystal Structures of K29-Linked diUb

We crystallized K29-linked Ub dimers, and the structure was determined by molecular replacement and refined to the final statistics as shown in Table 1. The asymmetric unit (ASU) contains two K29-linked diUb molecules (Figure S2A). The distal Ub is bound via its C terminus to K29 of the proximal Ub, and there is visible electron density for the isopeptide linkage (Figure S2B). In the crystal structure, K29-linked diUb adopts an open conformation (Figure 2A). Contacts between the two isopeptide-linked Ub molecules are entirely polar and involve R42 and R72 on the distal Ub and E16, E18, and D21 of the proximal Ub (Figure 2B). The crystal structure reveals an extended conformation, but in solution, K29 chains may adopt other conformations.

This open conformation of K29 diUb is distinct from the completely extended conformation observed for K63 and M1 chains, where there is no contact between the two Ub moieties besides the linker (Figures 2C–2E). The hydrophobic I44 patch (made up of L8, I44, H68, and V70) and the I36 patch (made up of I36, L71, and L73) facilitate the majority of interactions between Ub and UBDs. Like K63 and M1 chains, these hydrophobic patches are exposed and accessible in the observed K29 diUb structure (Figure 2C).

Profiling Linkage Selectivity of UBDs

Because there are no UBDs characterized to date that bind to K29 chains, we next wanted to identify a UBD that can selectively recognize K29-linked chains. In order to systematically test linkage specificity of UBDs, we assembled and purified polyUb tetramers of seven different linkage types (Figure 3A). Each tetramer has a characteristic electrophoretic mobility, allowing

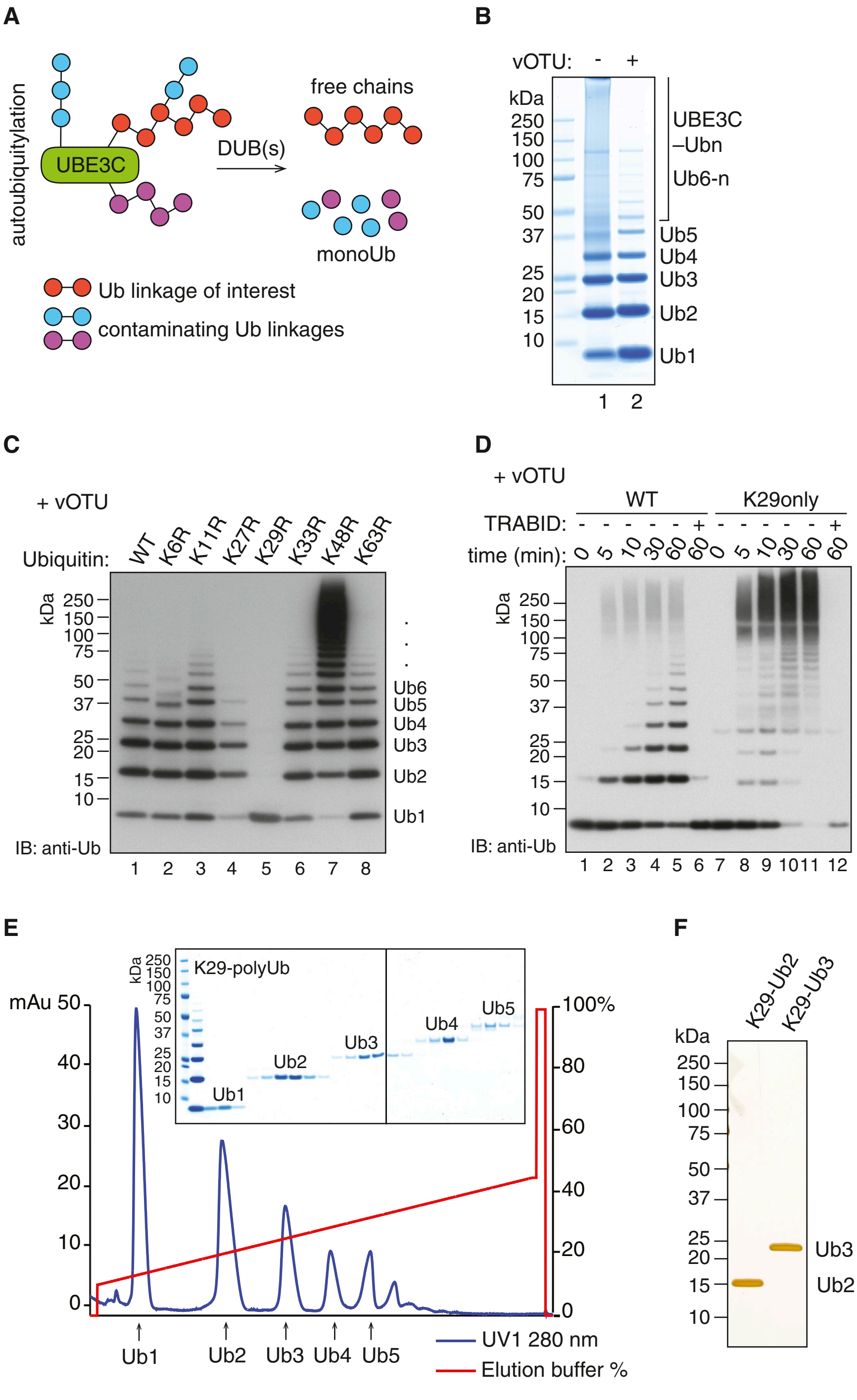

Figure 1. Assembly and Purification of K29-Linked PolyUb

(A) A schematic illustrates how E3 ligase and DUB(s) are used to assemble free polyUb chains.

(B) Large-scale assembly of polyUb chains by UBE3C in the presence of UBE1, UBE2D3, and Ub. The addition of vOTU releases free polyUb chains.

(C) Ubiquitylation assays of UBE3C in the presence of UBE1, UBE2D3, and wild-type Ub or K-to-R Ub mutants. vOTU was added at the end of the ubiquitylation reaction.

(D) Time course of ubiquitylation by UBE3C in the presence of UBE1, UBE2D3, vOTU, and wild-type Ub or K29only Ub mutant. To one half of the reaction stopped at 60 min, apyrase was added to quench ATP followed by DUB hydrolysis with TRABID.

(E) Purification of K29-linked diUb, triUb, tetraUb, and pentaUb by cation-exchange chromatography. Proteins from the corresponding peak fractions were visualized on Coomassie-stained SDS-PAGE gel.

(F) The K29-linked diUb and triUb purified in (E) are visualized in silver-stained SDS-PAGE gel.

See also Figure S1.

distinction between linkage types (Figure 3A). Note that when equal amounts of the different tetramers are present as detected by Coomassie and silver staining, detection by the anti-Ub antibody varies across linkages. To assess the linkage preferences of UBDs, we expressed and purified them as Halo-fusion proteins (Figure S3A). We chose to use Halo-tagged UBDs in this study to characterize polyUb binding as this tag does not dimerize and circumvents the artifacts of tag-induced avidity (Sims and Cohen, 2009). We first analyzed the linkage preference of the proteasome shuttling protein RAD23B that has been well characterized as a K48 binding protein (Rao and Sastry, 2002; Varadan et al., 2005). When compared across seven linkage types, the UBA domains of RAD23B still exhibit exquisite preference for K48 chains (Figure 3B). In contrast, the UBA domain of Ubiquilin-1 (UBQLN1) is non-selective and binds to all linkages, while the NZF of NPL4 binds to all linkages with the exception of K6 and K29 linkages.

Both K29 and K33 linkages are hydrolyzed by the catalytic domain of the DUB TRABID (Licchesi et al., 2012). TRABID is a multi-domain protein with the catalytic domain at the C terminus and UBDs consisting of three tandem NZF domains at the N terminus. We hypothesized that TRABID uses the NZF domains for targeting to its substrates, namely K29 and K33 polyUb. Previous studies using two or three linkage types have shown that TRABID NZFs can bind to K63 and M1 chains (Komander et al., 2009; Tran et al., 2008). When we tested the tandem NZFs of TRABID (NZF1-3), we find that in addition to K63 and M1 chains that they were previously described to bind to, the tandem NZF1-3 domains also bind to K11, K29, and K33 chains (Figure 3B).

In order to test if the binding properties of the isolated NZF domains varied in comparison with the tandem repeats, we

Table 1. Data Collection and Refinement Statistics

Data Collection	K29-linked diUb	K29-linked diUb–TRABID NZF1 complex
Wavelength (Å)	1.033	0.9795
Resolution range (Å)	60.05–2.30 (2.38–2.30)	76.10–3.00 (3.14–3.00)
Space group	$P2_1$	C2
Unit cell		
a, b, c (Å)	33.45 69.25 60.06	99.22 123.97 78.31
α, β, γ (°)	90.00 90.22 90.00	90.00 103.68 90.00
Total reflections	22,801 (2,317)	61,923 (5,809)
Unique reflections	11,542 (1,185)	17,755 (1,691)
Multiplicity	1.9 (2.0)	3.5 (3.4)
Completeness (%)	94.12 (92.88)	98.97 (95.32)
I/σI	8.71 (3.07)	15.64 (2.05)
R_{merge}	0.09621 (0.4483)	0.07207 (0.4869)
CC1/2	0.977 (0.614)	0.998 (0.914)
Refinement		
Number of atoms		
Protein	2,366	3,622
Ligand/ion	16	5
Water	43	0
R_{work}/R_{free}	0.1981/0.2456	0.2222/0.2702
Rmsd		
Bond lengths (Å)	0.018	0.005
Bond angles (°)	1.94	0.84
Average B-factor (Å^2)	29.2	81.9

The highest resolution shell is shown in parentheses.

expressed Halo-fusions spanning permutations of NZF1-3 and individual domains (Figure S3A). In comparison with NZF1-3, the double repeat UBDs NZF1-2 and NZF2-3 showed reduced binding to K63 and M1 chains while maintaining binding to K29 and K33 chains (Figure 3C, lanes 1–4). The individual NZF1 and NZF2 domains showed selectivity toward K29 and K33 chains, binding most of the input tetraUb (Figure 3C, lanes 5 and 6). Interestingly, the binding properties of NZF3 were very different from those of NZF1 and NZF2, as it was able to bind to K33 chains and showed weak binding to K6, K48, and K63 chains but did not interact with K29 chains (Figure 3C, lane 7). In summary, the individual NZF1 and NZF2 domains were selective for K29 and K33 chains, whereas the tandem NZF1-3 domains were able to bind to multiple linkages.

We focused on the NZF1 of TRABID, which binds to K29 and K33 polyUb and pulls down small amounts of M1 and K63 chains (Figure 3D). Our data predict that in a mixed environment, NZF1 will bind preferentially to K29 and K33 chains. We therefore performed a direct competition experiment where a mixture of chains was used in the pull-down assay and the differences in electrophoretic mobility were used to distinguish the different linkage types. As predicted, NZF1 preferentially binds to K29 and K33 chains over K63 and M1 linkages (Figure 3E). We next performed isothermal titration calorimetry (ITC) to compare the binding affinities of the individual NZF domains for diUb of different linkage types. In agreement with the results observed with the pull-down experiments, NZF1 binds to K29- and K33-linked diUb with higher affinities, 3.0 and 4.2 μM, respectively, whereas no detectable binding was observed for monoUb, M1 or K63 diUb (Figures 3F–3J). Although NZF2 has similar binding affinities for K29 and K33 chains as NZF1, the affinity of NZF3 for K33-linked diUb is too low to be measured by ITC (Figures S3B–S3D). In summary, these results reveal that the NZF1 of TRABID preferentially binds to K29 and K33 chains over K63 and M1.

Structural Basis for Linkage-Selective Recognition

To understand the structural basis of selective interaction between TRABID NZF1 and K29 chains, we determined the structure of TRABID NZF1 in complex with K29-linked diUb. Diffraction data were obtained at 3.0 Å resolution, and the structure was solved by molecular replacement and refined to the final statistics as shown in Table 1. TRABID NZF1 crystallized as a stoichiometric complex with K29 dimers, and the ASU contains five Ub and five NZF molecules (Figure S4A). The Ub moieties are arranged in such a way that the C terminus of one Ub points toward K29 of the next to form a continuous polyUb chain, and each NZF recognizes one diUb (Figures S4A and 4A). In the crystal lattice, this arrangement allows the chain to be extended infinitely and gives it the appearance of a helical filament (Figure S4A). The complexes found in the ASU superpose with a root-mean-square deviation (rmsd) between 0.363 and 0.883 (Figure S4B).

The structure of the NZF1 of TRABID is almost identical to the NZF domains of NPL4, TAB2, TAB3, and HOIL-1L (rmsd < 0.9 Å) (Alam et al., 2004; Kulathu et al., 2009; Sato et al., 2011, 2009). The four cysteine residues of TRABID NZF1, C10, C13, C24, and C27, coordinate a zinc ion. The TRABID NZF binds to the distal and proximal Ub moieties with buried surface areas of 350 and 320 Å^2, respectively. Although TRABID NZF1 interacts with both proximal and distal Ub, it does not directly recognize the K29-linked isopeptide bond of the diUb. The NZF domain of TRABID interacts with the I44-centered hydrophobic patch on the distal Ub (Figures 4B and 4C). In most NZFs, distal Ub recognition is mediated by a hydrophobic patch centered on the consensus T-F motif (T14 and Y15 in TRABID NZF1) (Alam et al., 2004; Haglund and Dikic, 2005) (Figure 4B). An additional hydrophobic residue present 10 amino acids away from the T-F motif (M26 in NZF1) also mediates interactions with the I44 patch on the distal Ub (Figures 4B and 4D). Thus in the NZF1 of TRABID, residues T14, Y15, and M26 interact with the distal Ub, and these residues are conserved in evolution (Figure 4D). Indeed, mutation of T14, Y15, or M26 to alanine is sufficient to disrupt the binding of TRABID NZF1 to K29 and K33 chains (Figure 4E).

In contrast to other NZF domains, TRABID NZF1 does not make contacts with either the I44 or F4 hydrophobic patches on the proximal Ub (Figures 4C and S5). Instead, TRABID NZF1 recognizes residues around K29 in the proximal Ub. Interestingly, TRABID M26 makes contacts with both the distal and proximal Ub moieties, and mutation of this residue disrupts NZF1 binding to K29 chains (Figures 4E and S3E). The overall conformation and relative orientation of the distal and proximal Ub moieties of the K29-linked diUb bound to TRABID NZF1

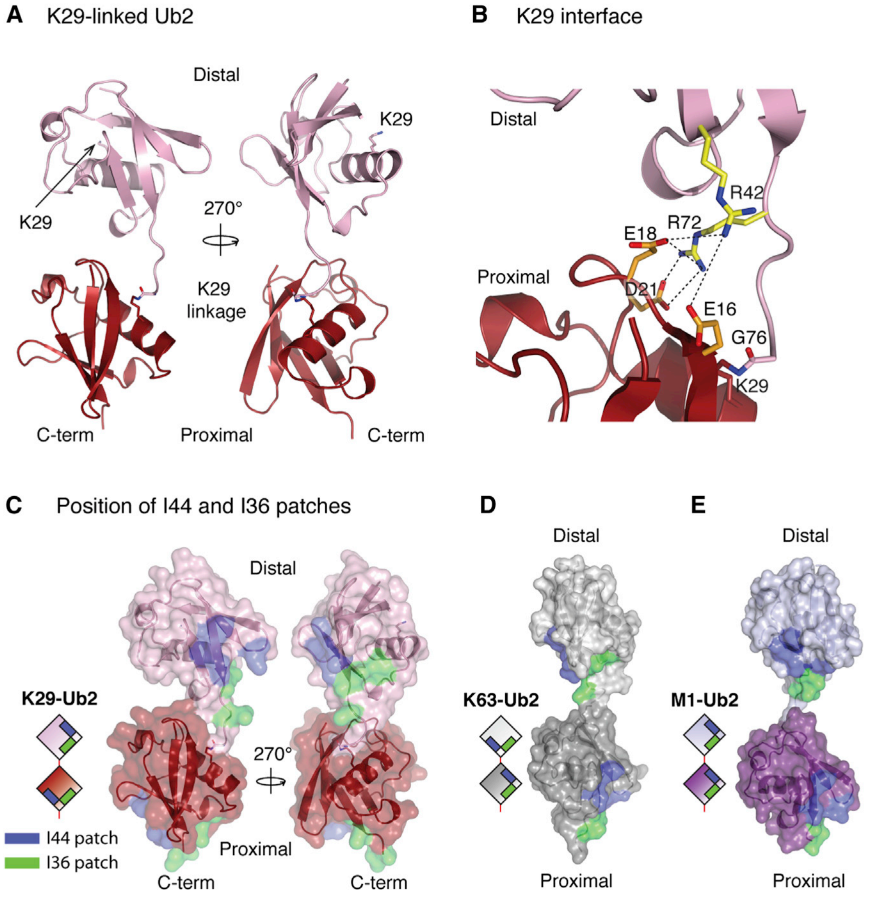

Figure 2. Crystal Structure of K29-Linked diUb

(A) The crystal structure of K29-linked diUb is shown in two orientations.

(B) Residues at the interface between the two Ub moieties are shown in stick representation. Polar interactions are shown as dotted lines.

(C) Positioning of surface hydrophobic patches in the structure of K29-linked diUb. A semi-transparent surface, colored blue for I44 patch (I44, L8, H68, and V70) and green for I36 patch (I36, L71, and L73), shows that the hydrophobic patches are not involved in the interface.

(D and E) Relative orientations of hydrophobic patches in K63-linked (D) and M1-linked (E) diUb. A semitransparent surface as in (C), showing the I36 and I44 patches (PDB accession numbers 2JF5 and 2W9N) (Komander et al., 2009).

See also Figure S2.

are considerably different from that of free K29-linked diUb (Figure 5A). When superposed on the distal Ub, the proximal Ub of the complex is rotated by 45° and moved by around 20 Å, thus remodeling the K29 chain. This remodeling enables TRABID NZF1 to make simultaneous interactions with both the distal and proximal moieties. A network of hydrogen bonds and ionic interactions further stabilizes the complex and is summarized in Table S3. TRABID NZF1, thus binds to K29 chains by binding to the I44 patch on the distal Ub and the Ub helix in the proximal Ub.

Linkage selective polyUb recognition requires simultaneous binding of the NZF to both distal and proximal moieties. If the binding of the NZF to the distal Ub is too strong, then polyUb binding will not depend on contribution from the proximal Ub, and hence polyUb binding will not be linkage selective. An example is the NZF domain of NPL4 that binds to monoUb via a distal Ub recognition site made up of T590, F591, and M602 and is therefore non-selective (Figures 3B and 5B) (Alam et al., 2004). In contrast, the NZF domain of TAB2 or TAB3 and TRABID NZF1 cannot bind to monoUb, as they have sub-optimal distal Ub recognition sites made up of T674, F675, and Q686 (TAB2) or T14, Y15, and M26 (TRABID) (Figure 3J) (Kulathu et al., 2009). Indeed, TRABID-NZF1 requires hydrophobic residues at each of these positions to bind polyUb (Figures 5C and 5D). As expected, strengthening distal Ub binding by mutating the hydrophilic Q686 of TAB2 to methionine (Q686M) not only increases binding of the mutant NZF to K63 chains but also enables it to bind to K29 and K33 chains (Figure 5E).

To address the importance of proximal Ub recognition, we focused on residue T25 of TRABID-NZF1. Although mutation of T25 to alanine does not affect TRABID-NZF1 binding to K29 or K33 chains (Figure 5F), the corresponding residue in TAB2 (E685) interacts with the proximal Ub and is important for binding K63 chains (Kulathu et al., 2009; Sato et al., 2009). This raises the possibility that the type of residue present at this position would influence specificity. Indeed, some T25 mutants show additional binding to K63 chains, suggesting that the residue at this position may influence linkage selectivity (Figure 5F). Interestingly, introducing a negatively charged residue at this position converts TRABID-NZF1 to be able to bind to multiple linkage types (Figure 5G). In summary, achieving linkage-selective polyUb recognition requires binding to the distal Ub with modest affinity and linkage-dependent interactions with the proximal Ub.

Heterotypic PolyUb Containing K29 Linkages Are Captured by TRABID

K29 linkages can be detected in proteomic studies (Dammer et al., 2011). Therefore, we hypothesized that the selective recognition of these linkages by TRABID could be exploited to capture K29 chains from cells. To test this hypothesis, we transiently expressed Flag-tagged versions of different TRABID constructs in HEK293 cells and analyzed the polyUb chains captured from cell extracts by TRABID (Figure 6A). Although full-length wild-type TRABID will bind to K29 linkages but hydrolyze them, the catalytically inactive mutant C443A should bind to K29 chains but not hydrolyze them (Figure S6A). Both full-length TRABID and the tandem NZFs capture high-molecular mass polyUb from resting cells (Figure 6A, lanes 2, 4, and 6). To detect if K29 linkages are present in the captured polyUb material, we

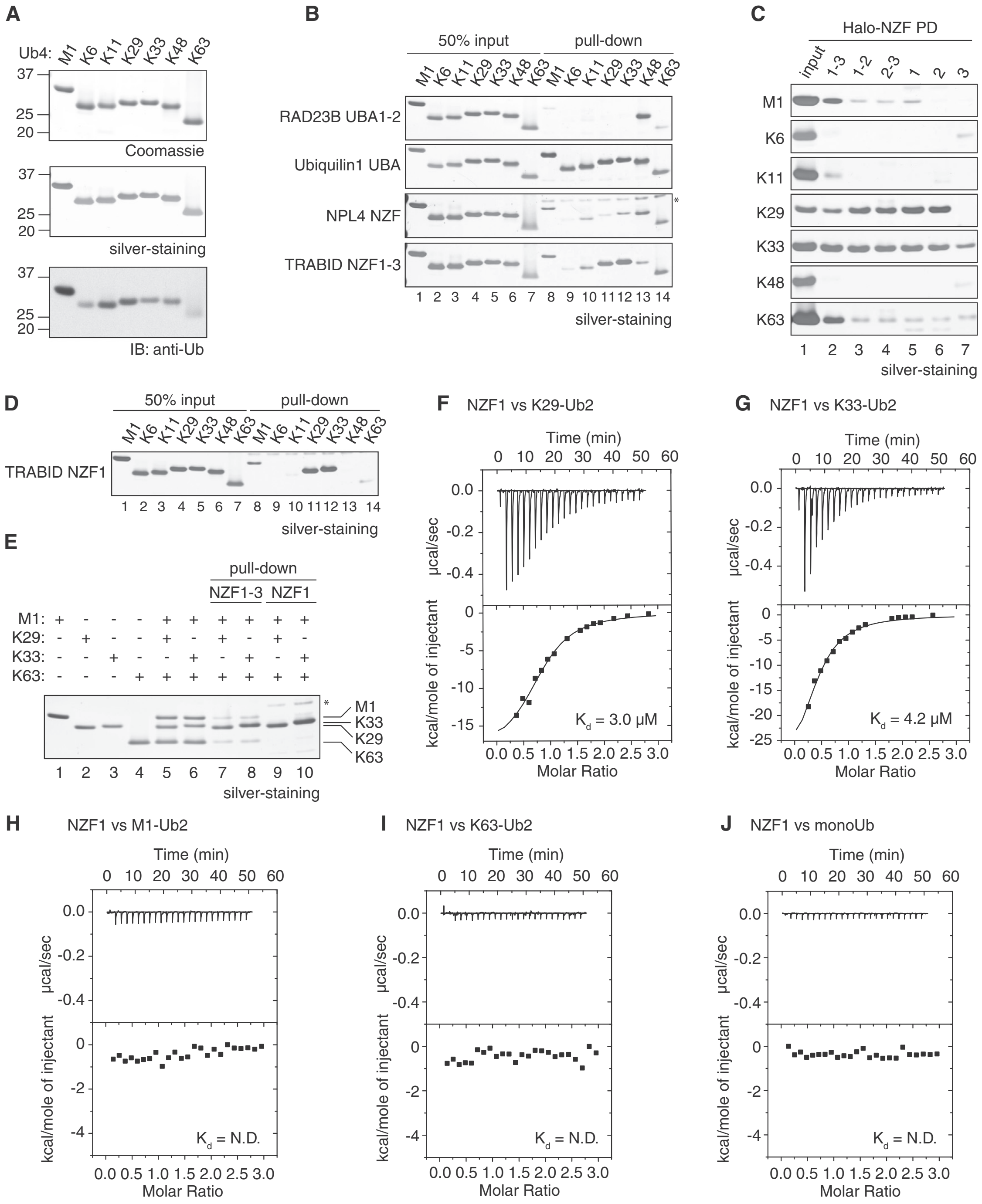

Figure 3. The NZF1 Domain of TRABID Is Highly Selective for K29 and K33 Chains

(A) Purified tetraUb of M1, K6, K11, K29, K33, K48, and K63 linkages were separated on 4% to 12% SDS-PAGE gel and visualized by Coomassie and silver staining, and anti-Ub immunoblotting.

(B) Linkage selectivity of different UBDs was investigated using pull-down assays. Halo-tagged RAD23B UBA1-2, Ubiquilin-1 UBA, NPL4 NZF, and TRABID NZF1-3 were used in pull-down assays with tetraUb of the indicated linkage types. The chains were visualized by silver-staining. Fifty percent of tetraUb input used in the pull-down assay was included as control. Asterisk shows NPL4 released from Halo resins.

(C) Pull-down assays to determine the linkage selectivity of the individual or tandem NZF domains of TRABID. Halo-tagged fusions of the indicated TRABID NZF domains were used in pull-down assays with tetraUb of seven different linkage types. One hundred percent of tetraUb input used in the pull-down assay was included as control.

(D) Linkage selectivity of TRABID NZF1 assayed as in (B).

(legend continued on next page)

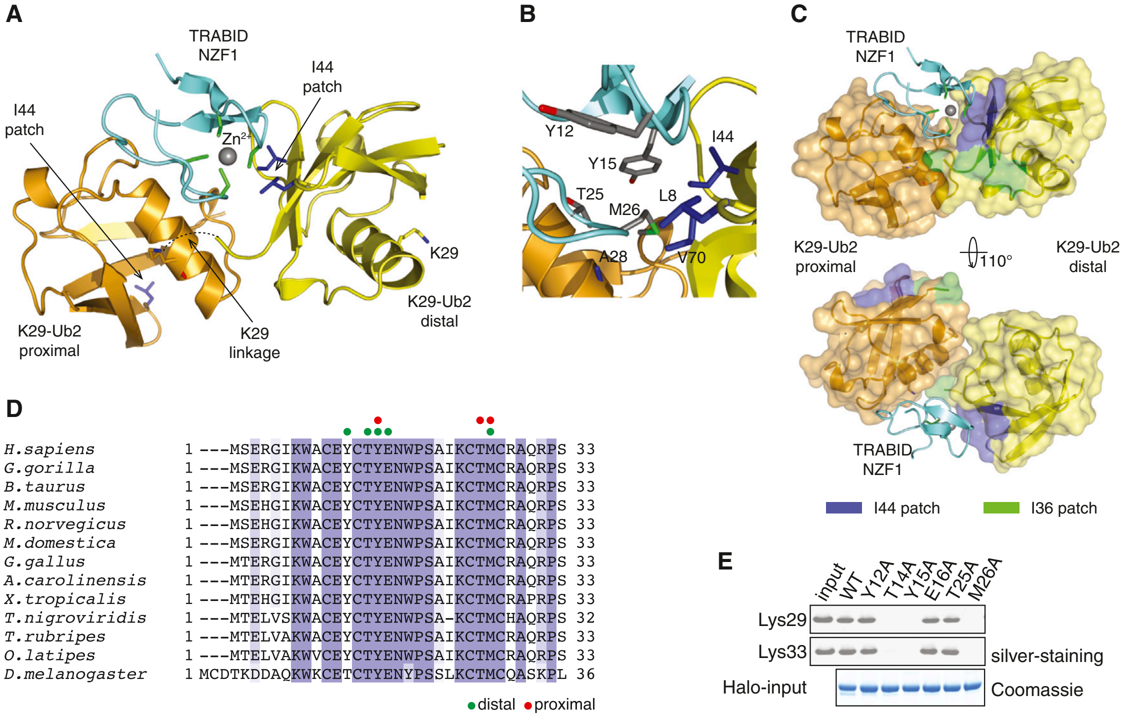

Figure 4. Crystal Structure of TRABID NZF1 in Complex with K29-Linked diUb

(A) Structure of TRABID NZF1 (cyan; zinc as gray sphere coordinated by cysteine residues [green]) bound to diUb (orange, proximal Ub; yellow, distal Ub). I44, V70, and L8 are shown as blue sticks.

(B) Selected residues of TRABID NZF1 (gray) and K29-diUb (blue) in the interface are shown.

(C) A semitransparent surface shows that only the Ile44 patch (blue) of the distal diUb interacts with TRABID NZF1.

(D) Sequence alignment of TRABID NZF1 from different species. Green and red circles indicate residues of TRABID NZF1 involved in binding to the distal and proximal Ub, respectively.

(E) Residues of TRABID NZF1 involved in Ub binding were mutated to alanine and the binding to K29- and K33-tetraUb was assayed as in Figure 3C. Halo-fusion proteins coupled to the resin were visualized on Coomassie-stained SDS-PAGE gel.

See also Figure S4.

incubated the pull-down with the DUB vOTU that does not hydrolyze M1, K27, and K29 linkages (Ritorto et al., 2014). Although incubation with vOTU reduced the high-molecular weight ubiquitylated species captured by wild-type TRABID down to monoUb, free polyUb chains resistant to vOTU cleavage were released from the TRABID C443A isolates (Figure 6A, lanes 3 and 5). Despite their poor expression in HEK293 cells, the tandem NZF domains of TRABID also captured ubiquitylated proteins and free polyUb chains of a similar pattern were released upon vOTU treatment (Figure 6A, lane 7, and Figure S6D).

To ensure that what we were observing was not an artifact of overexpressing TRABID domains in cells, we used bacterially expressed and purified Halo-fusions of TRABID NZFs to capture polyUb from cell extracts. Halo-NZF1-3, NZF1-2, and NZF2-3 all captured polyUb from cell extracts and released short chains when treated with vOTU (Figure 6B). Interestingly, the single NZF1 captured as much polyUb as the tandem fusion, while isolated NZF2 captured only a limited amount of polyUb (Figure 6B, lanes 4, 5, 10, and 11). However, the K33-selective NZF3 does not pull down any polyUb from cells.

We observed that only short chains are released from the Halo-NZF capture after vOTU treatment, in contrast to the longer chains released when the capture was done using Flag-tagged tandem NZF domains expressed in cells (Figures 6A and 6B). This may be because the Flag-tagged NZFs capture longer vOTU-resistant chains, or, alternatively, expression of the UBD in cells protected the chains from hydrolysis by cellular DUBs. To test this idea, we compared the polyUb that we captured from untransfected cells or from cells expressing NZF1-3 of TRABID. Further, we tested if our findings could be recapitulated using a different UBD, for which we used the UBA domain of Ubiquilin-1 (UBQLN1), which is a non-selective polyUb binder

(E) Linkage preference of TRABID NZF domains was assayed using a competition pull-down assay. K29- or K33-linked tetraUb was mixed with M1- and K63-tetraUb chains, and pull-downs performed using Halo-TRABID NZF1-3 or NZF1. Asterisk shows NZF1 released from Halo beads.

(F–J) ITC measurements for the NZF1 domain of TRABID with K29-Ub2 (F), K33-Ub2 (G), M1-Ub2 (H), K63-Ub2 (I), and monoUb (J). The K_d value for each measurement is indicated.

See also Figure S3.

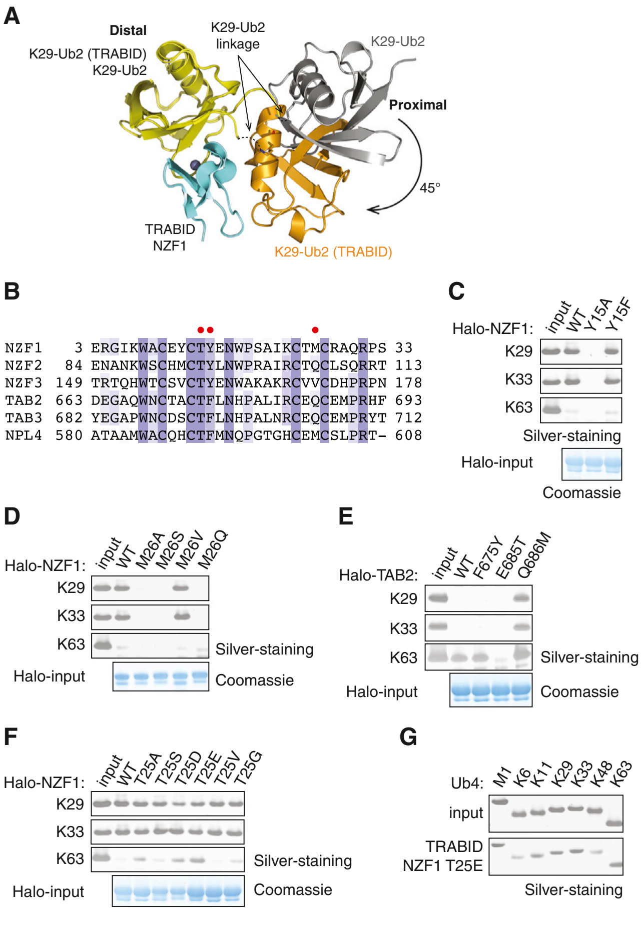

Figure 5. Determinants of NZF Binding to PolyUb

(A) Superposition of the distal Ub moieties (yellow) of K29-diUb alone and K29-diUb in the complex with TRABID NZF1. In the complex, the proximal moiety of free K29-diUb (gray) rotates approximately 45° to interact with NZF1 (cyan).

(B) Sequence alignment of TRABID NZF1, NZF2, and NZF3 with NZF domains of TAB2, TAB3, and NPL4. The hydrophobic residues making up the T-F/Φ binding patch are highlighted by red spheres.

(C and D) TRABID NZF1 mutants Y15 (C) and M26 (D) were assayed for binding to K29, K33, and K63 tetraUb.

(E) TAB2 NZF mutants were assayed as in (C).

(F) T25 of TRABID NZF1 was mutated and polyUb binding was assayed as in (C).

(G) Linkage specificity of TRABID NZF1 T25E mutant was assayed as in Figure 3B.

See also Figure S5.

capable of binding to several linkage types (Figure 3B). Immobilized Halo-NZF1-3, NZF1, and UBQLN1 all captured polyUb from extracts of both untransfected and transfected cells (Figure 6C). When treated with vOTU, polyUb chains of defined lengths were released from the captured material and significantly longer vOTU-resistant chains were present in pull-downs when cells expressed NZF1-3 (Figure 6C).

We have established a method to investigate K29 chains in cells that relies on TRABID-NZF1 binding to K29 and K33 chains and subsequent cleavage with a DUB that does not hydrolyze K29 linkages (Figure S7A). However, because vOTU does not hydrolyze M1 and K27 linkages in addition to K29 linkages, the short Ub chains released by vOTU from the high-molecular weight ubiquitylated mixture could contain these linkages. In order to determine the exact linkage type, we used linkage-selective DUBs. The polyUb species captured from HEK293 cell extracts using Halo-TRABID NZF1 beads were first incubated with vOTU. Interestingly, these short vOTU-resistant chains stayed bound to the Halo-TRABID NZF1 bead fraction, while the supernatant contained only monoUb (Figures 6D and S6B). The bead fraction containing the short chains was first washed to remove vOTU along with any of the released monoUb and then subsequently incubated with different linkage-selective DUBs. USP2, a linkage promiscuous DUB, hydrolyzed these short chains to monoUb, while DUBs that cleave M1 (OTULIN), K11 (CEZANNE), K48 (OTUB1), and K63 (AMSH) did not disassemble these short chains (Figure 6D). Incubation with TRABID cleaved these chains down to monoUb, indicating that the vOTU-resistant chains are K29-linked. Using pRM LC-MS/MS analysis, we further confirmed that these short vOTU resistant chains isolated from cell extracts are indeed K29-linked (Figures S7B and S7C).

K29 Linkages Are Present in Heterotypic Chains

The above experiments reveal that NZF1 captures K29 polyUb present in cells and suggest that these K29 linkages are present in heterotypic chains containing other linkages. We observe similar results when we perform pull-downs from extracts of different mouse tissues, suggesting that these heterotypic chains may be ubiquitous (Figure S8A). With available methods, it is challenging to determine the topology of these chains. Nevertheless, we used a combination of linkage-selective UBDs and DUBs to investigate the different possibilities of how these chains may be present (Figures 7A and 7B). To test if these heterotypic chains are made up of K48 and K29 linkages, we used Halo-RAD23B fusion that binds selectively to K48 chains to capture polyUb from cell extracts and the captured material was subsequently treated with vOTU (Figure 7C). Similar to what we observe with TRABID NZF1, short vOTU-resistant K29 chains were released, but in contrast to NZF1, these chains were not bound to RAD23B but are instead present in the supernatant (Figure 7C, lanes 3 and 4). Although short chains are also released from TRABID NZF1 captured material, these chains remain bound to the beads and the supernatants contained only monoUb (Figure 7C, lanes 7 and 8). Similar results were observed when the non-specific UBD of UBQLN1 was used (Figure 7C, lanes 11 and 12). We confirmed that these short chains are K29 linked, as subsequent incubation of these short chains with the DUB TRABID results in their hydrolysis down to monoUb (Figure S8C). Collectively, these results demonstrate that when we isolate polyUb chains from cells using a K29-selective UBD or a K48-selective UBD, we can detect the presence of short K29 chains.

In a converse experiment, we first captured polyUb from cell extracts using TRABID NZF1 and treated the captured material with TRABID that hydrolyzes K29 and K33 linkages (Figure 7D). The chains released into the supernatant were then subsequently incubated with Halo resin containing either RAD23B or TRABID NZF1. Importantly, the released chains bound only to RAD23B and not to TRABID NZF1.

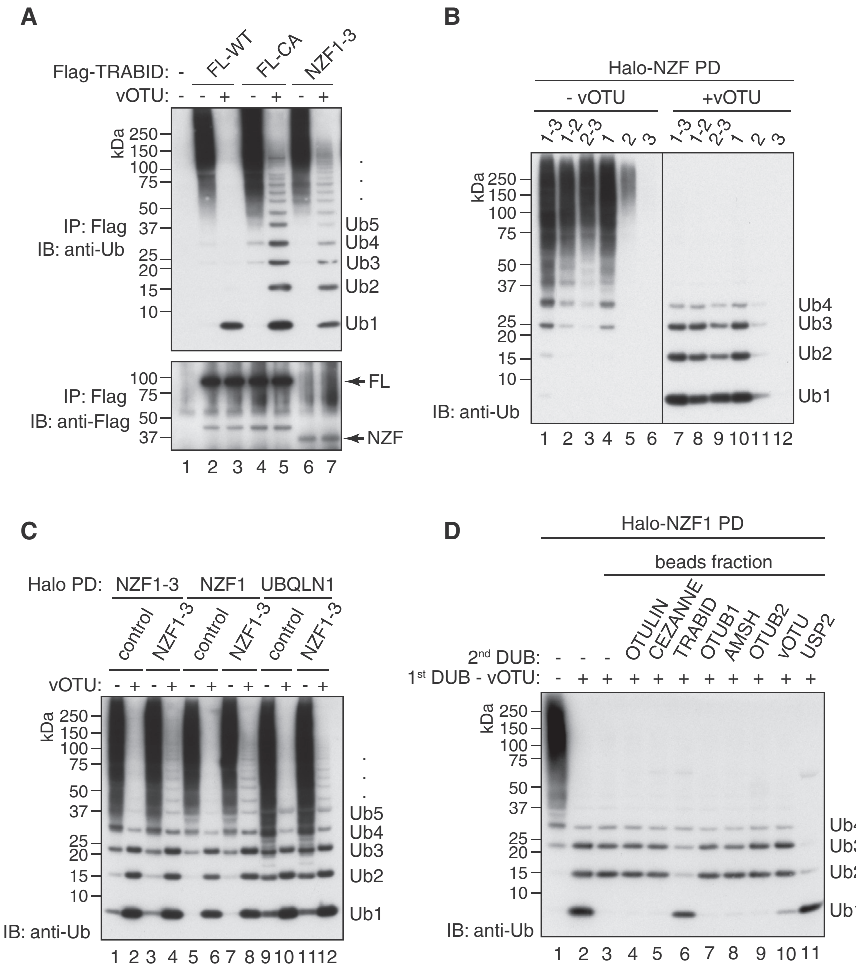

Figure 6. Isolation and Analysis of K29 Chains from Mammalian Cells

(A) Analysis of polyUb captured from HEK293 cells transiently expressing Flag-tagged full-length TRABID (FL-WT), full-length catalytically dead (FL-CA), or tandem NZF1-3 domains (NZF1-3). One half of the Flag-immunopurified sample was incubated with vOTU that hydrolyzes all linkage types excepting M1, K27, and K29 linkages and visualized by anti-Ub immunoblotting.

(B) PolyUb chains from HEK293 extracts were captured using indicated bacterially expressed Halo NZF domains of TRABID, followed by DUB treatment as in (A).

(C) PolyUb chains from untransfected (control) or Flag-TRABID NZF1-3 (NZF1-3)-expressing HEK293 cells were captured using Halo-TRABID NZF1-3, TRABID NZF1, or Ubiquilin-1 (UBQLN1) UBA domain. The isolated polyUb chains were either left untreated or treated with vOTU.

(D) Immunobloting analysis of polyUb chains captured from HEK293 extracts using the minimal NZF1 that is selective for K29 and K33 linkages. Presence of K29 linkages was assayed by incubation with vOTU, which does not hydrolyze K29 linkages. The vOTU-resistant polyUb chains that bound to Halo-TRABID NZF1 were separated from monoUb, washed, and the linkage-types of these chains were assayed by incubation with a panel of DUBs that hydrolyze different linkage types.

See also Figures S6 and S7.

To provide further evidence for the existence of heterotypic chains containing K29 linkages, we performed serial polyUb capture experiments (Figures S8D and S8E). When TRABID NZF1 was used in the first pull-down, this captured all the K29-linked chains from cells. A second pull-down using a K48-specific UBD captured polyUb from the cell extracts, but these did not contain any K29 linkages (Figure S8D). In a converse experiment, if the first capture was done with a K48-specific UBD, all the K29 chains were present in this fraction, and no further K29 chains could be isolated in a subsequent capture with a K29-specific UBD (Figure S8D). These data further support our hypothesis that K29 linkages are present in chains that also contain K48 linkages. Moreover, these results also suggest the existence of two pools of K48 chains, one with and another without K29 linkages. Taken together, these data show that multiple blocks of K29-linked polyUb exist in cells as part of heterotypic chains that also contain K48 linkages.

DISCUSSION

K29 linkages are an abundant atypical linkage type, but their cellular roles are not completely understood, as tools such as linkage-specific antibodies are not available to study these chains. Our work shows that K29-linked polyUb adopt an open conformation and are recognized by the NZF domains of TRABID and has revealed the use of NZF1 of TRABID as a tool to study K29-linked polyUb in cells. Importantly, the use of this tool has shown that K29 chains exist as heterotypic chains exclusively, mainly containing K48 linkages, and furthermore that K48 chains exist in two distinct pools, one containing K29 linkages and one without.

The ability to enzymatically assemble K29 chains in vitro is an advance that will pave the way for many subsequent studies. For the enzymatic assembly of K29 polyUb chains, we have combined the HECT ligase UBE3C with the DUB vOTU. Although we have synthetically created a Ub chain-editing complex for our purpose, such complexes containing ligases and DUBs have been described in vivo. These complexes are an efficient mechanism to rapidly remodel the topology of polyUb on a substrate and thereby modulate the outcome of the Ub signal (Newton et al., 2008; Wertz et al., 2004). Interestingly, several DUBs are found associated with Ub ligases (Sowa et al., 2009), and we speculate that these uncharacterized complexes may assemble atypical chains in cells.

UBE3C, the enzyme used for assembling K29 chains, is involved in regulating protein turnover (Chu et al., 2013). Recent work demonstrated that UBE3C associates with the proteasome and its levels at the proteasome increase upon proteasome inhibition (Besche et al., 2014; Crosas et al., 2006). In yeast, the UBE3C homolog Hul5 functions as an E4 to extend Ub chains

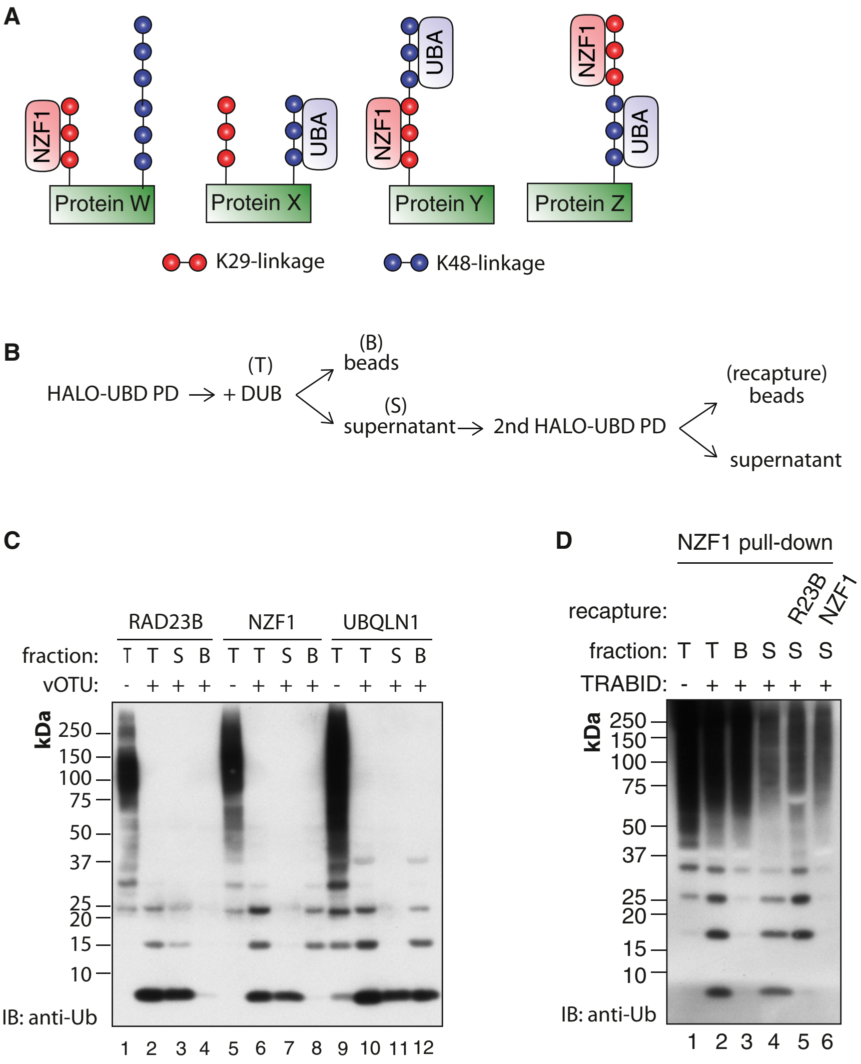

Figure 7. K29-Linked Chains Are Present within Mixed or Branched Heterotypic Chains

(A) Cartoon illustrating the different possibilities of how K29 linkages may be present in the heterotypic chains captured by TRABID NZF1.

(B) A schematic illustrates the experimental procedure for determining the linkage composition of the mixed or branched heterotypic chains isolated by the linkage-selective UBDs.

(C) PolyUb chains from the HEK293 were first captured using Halo-tagged RAD23B (K48-selective binder), TRABID NZF1 (K29- and K33-selective binder), or UBQLN1 (non-selective binder). Subsequently, samples in the indicated lanes were treated with vOTU. The total capture (T), supernatant (S), and beads (B) fractions after the DUB reaction were analyzed separately as shown in (B).

(D) Recapture assay using linkage-selective UBDs to determine the linkage type of the chains remaining after removing K29 linkages. PolyUb chains captured from HEK293 cell extracts using Halo-NZF1 were incubated with TRABID that preferentially hydrolyzes K29 and K33 linkages. The polyUb chains that were released into the supernatant fraction after removing K29 linkages were recaptured using either the K48-selective Halo-RAD23B or the K29 and K33 selective Halo-TRABID NZF1.

See also Figure S8.

on ubiquitylated substrates to promote degradation of misfolded proteins (Fang et al., 2011). Such an extension of polyUb on substrates at the proteasome by UBE3C may increase the residence time of the substrate at the proteasome and leads to the speculation that K29-K48 heterotypic chains provide an important signal for difficult to degrade substrates to be proteolyzed.

The assembly of K29 chains has not only allowed us to structurally characterize these linkages but has also provided insights into their recognition and clues into their biological roles. Until recently, K48 and K63 chains were the only linkage types that could be assembled in vitro, which permitted better analyses of these linkages compared with the remaining six linkages. With the availability of tetraUb of seven linkage types, we can now systematically profile UBDs to reveal their polyUb binding characteristics. The NZF domains of TRABID were previously described as selective K63 and M1 binders, but the availability of tetraUb of seven different linkage types has allowed a more complete assessment of linkage selectivity and led us to discover that the individual NZFs of TRABID interact specifically with K29 and K33 polyUb. Excitingly, this provides the description of a UBD that is selective for these uncharacterized linkages.

In all the structural studies analyzing polyUb binding by NZF domains, the NZF does not make direct contact with the linker between the two Ub moieties but rather exploits the relative orientation of surface hydrophobic patches on Ub (Husnjak and Dikic, 2012). NZF1 of TRABID remodels the open conformation of K29 chains to satisfy a two-sided interaction. In contrast to other NZF domains that recognize the proximal Ub via one of the hydrophobic patches, TRABID NZF1 uses a distinct mode of Ub recognition whereby it makes contacts with the helix in Ub. Given that both K29 and K33 lie on the Ub helix, we speculate that NZF1 and NZF2 may use similar mechanisms to bind to both K29 and K33 chains. In support of this idea, we find that mutations that disrupt binding to K29 chains also affect binding to K33 chains. In tandem, the UBDs have a different polyUb binding preference that could be explained in part by the relative spatial organization of the domains, using a mechanism similar to that described for the tandem UIMs of RAP80 (Sims and Cohen, 2009).

Our results suggest that rheostat-like modulation of the affinity of the NZF for distal and proximal Ub moieties is central to achieving linkage-selective polyUb recognition. NZF domains like that of NPL4 can bind strongly to the distal Ub, thus making them non-specific binders. In contrast, NZF domains like that of TAB2 and TRABID, which have weaker binding to distal Ub, now

rely on additional interactions mediated with the proximal Ub, and these interactions with the proximal Ub are determinants of linkage-selective recognition. It is tempting to speculate that by modulating the proximal Ub binding site designer NZFs specific for desired linkage types can be created.

The availability of linkage-specific antibodies and polyUb sensors has greatly advanced our understanding of the cellular roles and the spatiotemporal dynamics of M1, K11, K48, and K63 polyUb in cells (Matsumoto et al., 2010; Newton et al., 2008; van Wijk et al., 2012). We exploited our identification of UBDs that selectively bind to these two linkages to use these NZFs as an affinity reagent to capture K29 cells from cells. In agreement with previous proteomic analyses, we can detect K29 linkages in resting mammalian cells and in different mouse tissues. Unexpectedly, we find that K29 chains are present as heterotypic chains. It has only recently emerged that mixed and branched polyUb chains serve specialized signaling functions (Emmerich et al., 2013; Meyer and Rape, 2014). Although we have been able to readily isolate heterotypic chains containing short K29-linked polyUb containing no more than four moieties, longer K29 chains were detected only when the tandem NZFs of TRABID was expressed in cells. This suggests that K29 chains do not grow very long and are hydrolyzed by cellular DUBs. Because several proteomic analyses find that the abundance of K29 linkages increases upon proteasomal inhibition, we speculate that short K29-linked chains may be added as capping modifications. Further, the helical nature of K29 polymers, as observed in the structure of K29 chains in complex with TRABID NZF1, may denote a specialized signal.

Determining the precise topology of these heterotypic polyUb chains and whether they are present as mixed or branched chains will be challenging. It will be of great interest to investigate the conditions under which these chains are made in cells and the precise biological processes that are regulated by these heterotypic chains. Given the paucity of tools available to study different linkage types, our work reveals how linkage selective UBDs can be exploited to capture particular linkages from cells and provides a valuable tool to investigate cellular roles of this enigmatic linkage.

EXPERIMENTAL PROCEDURES

An extended version can be found in Supplemental Experimental Procedures.

K29-Linked PolyUb Assembly

Large-scale K29-linked polyUb chain assembly was carried out in 1.5-ml reactions of 25 mg Ub (Sigma), 644 nM UBE1, 9.5 μM UBE2D3, 3 μM UBE3C, 10 mM ATP, 50 mM Tris-HCl (pH 7.5), 10 mM $MgCl_2$, and 0.6 mM DTT at 30°C overnight. To release K29-polyUb chains, a total of 2 μM vOTU and 5 mM DTT were added in to the assembly reaction and incubated further at 30°C overnight. Chains of defined lengths were purified as described in Supplemental Experimental Procedures.

Preparation of Halo-UBD Resins

UBDs used in this study were expressed in *E. coli* as GST-Halo fusion protein (see Table S1). GST was removed using C3 protease. Halo-tagged UBDs (21 nmol) were incubated with 200 μl of the HaloLink resin (Promega) in 1 ml binding buffer (50 mM Tris-HCl [pH 7.5], 150 mM NaCl, 0.05% NP-40, 1 mM DTT) overnight at 4°C. Pull-down and analysis of polyUb chains using Halo-tagged UBDs were performed as described in Supplemental Experimental Procedures.

ACCESSION NUMBERS

The Protein Data Bank (PDB) accession numbers for the structures reported in this paper are 4S22 and 4S1Z.

SUPPLEMENTAL INFORMATION

Supplemental Information includes Supplemental Experimental Procedures, eight figures, and four tables and can be found with this article online at http://dx.doi.org/10.1016/j.molcel.2015.01.041.

AUTHOR CONTRIBUTIONS

Y.A.K. and Y.K. designed, performed, and analyzed all experiments. S.A.A.R. performed crystallographic analyses. D.G.C. and N.A.M. did the mass spectrometry analyses. C.J. helped with protein purification. R.T. cloned all the DNA constructs. Y.K. wrote the manuscript with input from all authors.

ACKNOWLEDGMENTS

We would like to thank A. Knebel, R.A. Ewan, and S. Weidlich for reagents; K. Airey and J. Stark for tissue culture support; and R.J. Martínez-Torres, T. Cardote, and C. Galdeano for help with ITC. We thank H. Walden, R.T. Hay, M. Swamy, V.K. Chaugule, and members of the Kulathu laboratory for discussions and critical comments on the manuscript. Crystallographic data were collected at the European Synchrotron Radiation Facility beamline ID23-1 and Diamond Light Source beamline I24. This work was supported by the Medical Research Council UK, Wellcome Trust (grant number 094090) and the pharmaceutical companies supporting the Division of Signal Transduction Therapy (AstraZeneca, Boehringer-Ingelheim, GlaxoSmithKline, Merck KGaA, Janssen Pharmaceutica, and Pfizer).

Received: September 4, 2014
Revised: December 2, 2014
Accepted: January 27, 2015
Published: March 5, 2015

REFERENCES

Alam, S.L., Sun, J., Payne, M., Welch, B.D., Blake, B.K., Davis, D.R., Meyer, H.H., Emr, S.D., and Sundquist, W.I. (2004). Ubiquitin interactions of NZF zinc fingers. EMBO J. *23*, 1411–1421.

Behrends, C., and Harper, J.W. (2011). Constructing and decoding unconventional ubiquitin chains. Nat. Struct. Mol. Biol. *18*, 520–528.

Besche, H.C., Sha, Z., Kukushkin, N.V., Peth, A., Hock, E.M., Kim, W., Gygi, S., Gutierrez, J.A., Liao, H., Dick, L., and Goldberg, A.L. (2014). Autoubiquitination of the 26S proteasome on Rpn13 regulates breakdown of ubiquitin conjugates. EMBO J. *33*, 1159–1176.

Boname, J.M., Thomas, M., Stagg, H.R., Xu, P., Peng, J., and Lehner, P.J. (2010). Efficient internalization of MHC I requires lysine-11 and lysine-63 mixed linkage polyubiquitin chains. Traffic *11*, 210–220.

Bremm, A., Freund, S.M.V., and Komander, D. (2010). Lys11-linked ubiquitin chains adopt compact conformations and are preferentially hydrolyzed by the deubiquitinase Cezanne. Nat. Struct. Mol. Biol. *17*, 939–947.

Chastagner, P., Israël, A., and Brou, C. (2006). Itch/AIP4 mediates Deltex degradation through the formation of K29-linked polyubiquitin chains. EMBO Rep. *7*, 1147–1153.

Chau, V., Tobias, J.W., Bachmair, A., Marriott, D., Ecker, D.J., Gonda, D.K., and Varshavsky, A. (1989). A multiubiquitin chain is confined to specific lysine in a targeted short-lived protein. Science *243*, 1576–1583.

Chu, B.W., Kovary, K.M., Guillaume, J., Chen, L.C., Teruel, M.N., and Wandless, T.J. (2013). The E3 ubiquitin ligase UBE3C enhances proteasome processivity by ubiquitinating partially proteolyzed substrates. J. Biol. Chem. *288*, 34575–34587.

Crosas, B., Hanna, J., Kirkpatrick, D.S., Zhang, D.P., Tone, Y., Hathaway, N.A., Buecker, C., Leggett, D.S., Schmidt, M., King, R.W., et al. (2006). Ubiquitin chains are remodeled at the proteasome by opposing ubiquitin ligase and deubiquitinating activities. Cell *127*, 1401–1413.

Dammer, E.B., Na, C.H., Xu, P., Seyfried, N.T., Duong, D.M., Cheng, D., Gearing, M., Rees, H., Lah, J.J., Levey, A.I., et al. (2011). Polyubiquitin linkage profiles in three models of proteolytic stress suggest the etiology of Alzheimer disease. J. Biol. Chem. *286*, 10457–10465.

Datta, A.B., Hura, G.L., and Wolberger, C. (2009). The structure and conformation of Lys63-linked tetraubiquitin. J. Mol. Biol. *392*, 1117–1124.

El Oualid, F., Merkx, R., Ekkebus, R., Hameed, D.S., Smit, J.J., de Jong, A., Hilkmann, H., Sixma, T.K., and Ovaa, H. (2010). Chemical synthesis of ubiquitin, ubiquitin-based probes, and diubiquitin. Angew. Chem. Int. *49*, 10149–10153.

Emmerich, C.H., Ordureau, A., Strickson, S., Arthur, J.S.C., Pedrioli, P.G.A., Komander, D., and Cohen, P. (2013). Activation of the canonical IKK complex by K63/M1-linked hybrid ubiquitin chains. Proc. Natl. Acad. Sci. U S A *110*, 15247–15252.

Fang, N.N., Ng, A.H.M., Measday, V., and Mayor, T. (2011). Hul5 HECT ubiquitin ligase plays a major role in the ubiquitylation and turnover of cytosolic misfolded proteins. Nat. Cell Biol. *13*, 1344–1352.

Haglund, K., and Dikic, I. (2005). Ubiquitylation and cell signaling. EMBO J. *24*, 3353–3359.

Hay-Koren, A., Caspi, M., Zilberberg, A., and Rosin-Arbesfeld, R. (2011). The EDD E3 ubiquitin ligase ubiquitinates and up-regulates beta-catenin. Mol. Biol. Cell *22*, 399–411.

Hospenthal, M.K., Freund, S.M.V., and Komander, D. (2013). Assembly, analysis and architecture of atypical ubiquitin chains. Nat. Struct. Mol. Biol. *20*, 555–565.

Hurley, J.H., Lee, S., and Prag, G. (2006). Ubiquitin-binding domains. Biochem. J. *399*, 361–372.

Husnjak, K., and Dikic, I. (2012). Ubiquitin-binding proteins: decoders of ubiquitin-mediated cellular functions. Annu. Rev. Biochem. *81*, 291–322.

Johnson, E.S., Ma, P.C., Ota, I.M., and Varshavsky, A. (1995). A proteolytic pathway that recognizes ubiquitin as a degradation signal. J. Biol. Chem. *270*, 17442–17456.

Koegl, M., Hoppe, T., Schlenker, S., Ulrich, H.D., Mayer, T.U., and Jentsch, S. (1999). A novel ubiquitination factor, E4, is involved in multiubiquitin chain assembly. Cell *96*, 635–644.

Komander, D., and Rape, M. (2012). The ubiquitin code. Annu. Rev. Biochem. *81*, 203–229.

Komander, D., Reyes-Turcu, F., Licchesi, J.D.F., Odenwaelder, P., Wilkinson, K.D., and Barford, D. (2009). Molecular discrimination of structurally equivalent Lys 63-linked and linear polyubiquitin chains. EMBO Rep. *10*, 466–473.

Kulathu, Y., and Komander, D. (2012). Atypical ubiquitylation - the unexplored world of polyubiquitin beyond Lys48 and Lys63 linkages. Nat. Rev. Mol. Cell Biol. *13*, 508–523.

Kulathu, Y., Akutsu, M., Bremm, A., Hofmann, K., and Komander, D. (2009). Two-sided ubiquitin binding explains specificity of the TAB2 NZF domain. Nat. Struct. Mol. Biol. *16*, 1328–1330.

Kumar, K.S.A., Spasser, L., Erlich, L.A., Bavikar, S.N., and Brik, A. (2010). Total chemical synthesis of di-ubiquitin chains. Angew. Chem. Int. Ed. Engl. *49*, 9126–9131.

Licchesi, J.D.F., Mieszczanek, J., Mevissen, T.E.T., Rutherford, T.J., Akutsu, M., Virdee, S., El Oualid, F., Chin, J.W., Ovaa, H., Bienz, M., and Komander, D. (2012). An ankyrin-repeat ubiquitin-binding domain determines TRABID's specificity for atypical ubiquitin chains. Nat. Struct. Mol. Biol. *19*, 62–71.

Matsumoto, M.L., Wickliffe, K.E., Dong, K.C., Yu, C., Bosanac, I., Bustos, D., Phu, L., Kirkpatrick, D.S., Hymowitz, S.G., Rape, M., et al. (2010). K11-linked polyubiquitination in cell cycle control revealed by a K11 linkage-specific antibody. Mol. Cell *39*, 477–484.

Meyer, H.-J., and Rape, M. (2014). Enhanced protein degradation by branched ubiquitin chains. Cell *157*, 910–921.

Newton, K., Matsumoto, M.L., Wertz, I.E., Kirkpatrick, D.S., Lill, J.R., Tan, J., Dugger, D., Gordon, N., Sidhu, S.S., Fellouse, F.A., et al. (2008). Ubiquitin chain editing revealed by polyubiquitin linkage-specific antibodies. Cell *134*, 668–678.

Peng, J., Schwartz, D., Elias, J.E., Thoreen, C.C., Cheng, D., Marsischky, G., Roelofs, J., Finley, D., and Gygi, S.P. (2003). A proteomics approach to understanding protein ubiquitination. Nat. Biotechnol. *21*, 921–926.

Peterson, A.C., Russell, J.D., Bailey, D.J., Westphall, M.S., and Coon, J.J. (2012). Parallel reaction monitoring for high resolution and high mass accuracy quantitative, targeted proteomics. Mol. Cell. Proteomics *11*, 1475–1488.

Pickart, C.M., and Raasi, S. (2005). Controlled synthesis of polyubiquitin chains. Methods Enzymol. *399*, 21–36.

Rao, H., and Sastry, A. (2002). Recognition of specific ubiquitin conjugates is important for the proteolytic functions of the ubiquitin-associated domain proteins Dsk2 and Rad23. J. Biol. Chem. *277*, 11691–11695.

Ritorto, M.S., Ewan, R., Perez-Oliva, A.B., Knebel, A., Buhrlage, S.J., Wightman, M., Kelly, S.M., Wood, N.T., Virdee, S., Gray, N.S., et al. (2014). Screening of DUB activity and specificity by MALDI-TOF mass spectrometry. Nat. Commun. *5*, 4763.

Rohaim, A., Kawasaki, M., Kato, R., Dikic, I., and Wakatsuki, S. (2012). Structure of a compact conformation of linear diubiquitin. Acta Crystallogr. D Biol. Crystallogr. *68*, 102–108.

Sato, Y., Yoshikawa, A., Yamashita, M., Yamagata, A., and Fukai, S. (2009). Structural basis for specific recognition of Lys 63-linked polyubiquitin chains by NZF domains of TAB2 and TAB3. EMBO J. *28*, 3903–3909.

Sato, Y., Fujita, H., Yoshikawa, A., Yamashita, M., Yamagata, A., Kaiser, S.E., Iwai, K., and Fukai, S. (2011). Specific recognition of linear ubiquitin chains by the Npl4 zinc finger (NZF) domain of the HOIL-1L subunit of the linear ubiquitin chain assembly complex. Proc. Natl. Acad. Sci. U S A *108*, 20520–20525.

Sims, J.J., and Cohen, R.E. (2009). Linkage-specific avidity defines the lysine 63-linked polyubiquitin-binding preference of rap80. Mol. Cell *33*, 775–783.

Sowa, M.E., Bennett, E.J., Gygi, S.P., and Harper, J.W. (2009). Defining the human deubiquitinating enzyme interaction landscape. Cell *138*, 389–403.

Spence, J., Sadis, S., Haas, A.L., and Finley, D. (1995). A ubiquitin mutant with specific defects in DNA repair and multiubiquitination. Mol. Cell. Biol. *15*, 1265–1273.

Tran, H., Hamada, F., Schwarz-Romond, T., and Bienz, M. (2008). Trabid, a new positive regulator of Wnt-induced transcription with preference for binding and cleaving K63-linked ubiquitin chains. Genes Dev. *22*, 528–542.

van Wijk, S.J.L., Fiskin, E., Putyrski, M., Pampaloni, F., Hou, J., Wild, P., Kensche, T., Grecco, H.E., Bastiaens, P., and Dikic, I. (2012). Fluorescence-based sensors to monitor localization and functions of linear and K63-linked ubiquitin chains in cells. Mol. Cell *47*, 797–809.

Varadan, R., Assfalg, M., Raasi, S., Pickart, C., and Fushman, D. (2005). Structural determinants for selective recognition of a Lys48-linked polyubiquitin chain by a UBA domain. Mol. Cell *18*, 687–698.

Virdee, S., Ye, Y., Nguyen, D.P., Komander, D., and Chin, J.W. (2010). Engineered diubiquitin synthesis reveals Lys29-isopeptide specificity of an OTU deubiquitinase. Nat. Chem. Biol. *6*, 750–757.

Wang, C., Deng, L., Hong, M., Akkaraju, G.R., Inoue, J., and Chen, Z.J. (2001). TAK1 is a ubiquitin-dependent kinase of MKK and IKK. Nature *412*, 346–351.

Wertz, I.E., O'Rourke, K.M., Zhou, H., Eby, M., Aravind, L., Seshagiri, S., Wu, P., Wiesmann, C., Baker, R., Boone, D.L., et al. (2004). De-ubiquitination and ubiquitin ligase domains of A20 downregulate NF-kappaB signalling. Nature *430*, 694–699.

Ye, Y., and Rape, M. (2009). Building ubiquitin chains: E2 enzymes at work. Nat. Rev. Mol. Cell Biol. *10*, 755–764.

You, J., and Pickart, C.M. (2001). A HECT domain E3 enzyme assembles novel polyubiquitin chains. J. Biol. Chem. *276*, 19871–19878.

Ziv, I., Matiuhin, Y., Kirkpatrick, D.S., Erpapazoglou, Z., Leon, S., Pantazopoulou, M., Kim, W., Gygi, S.P., Haguenauer-Tsapis, R., Reis, N., et al. (2011). A perturbed ubiquitin landscape distinguishes between ubiquitin in trafficking and in proteolysis. Mol. Cell. Proteomics *10*, M111.009753.

Molecular Cell, Volume *58*

Supplemental Information

K29-Selective Ubiquitin Binding Domain Reveals Structural Basis of Specificity and Heterotypic Nature of K29 Polyubiquitin

Yosua Adi Kristariyanto, Syed Arif Abdul Rehman, David G. Campbell, Nicholas A. Morrice, Clare Johnson, Rachel Toth, and Yogesh Kulathu

K29-selective ubiquitin binding domain reveals structural basis of specificity and heterotypic nature of K29 polyubiquitin

Yosua Adi Kristariyanto, Syed Arif Abdul Rehman, David G. Campbell, Nicholas A. Morrice[1], Clare Johnson, Rachel Toth & Yogesh Kulathu[#]

Supplemental Information

MRC Protein Phosphorylation and Ubiquitylation Unit, College of Life Sciences, University of Dundee, Dow Street, Dundee DD1 5EH, UK
[1]Present address: AB Sciex UK Limited, Warrington, Cheshire WA1 1RX, UK

[#]Correspondence should be addressed to Yogesh Kulathu, MRC Protein Phosphorylation and Ubiquitylation Unit, College of Life Sciences, University of Dundee, Dow Street, Dundee DD1 5EH, UK.
Email: ykulathu@dundee.ac.uk, Tel: +44 1382 388163, Fax: +44 1382 223778

Figure S1

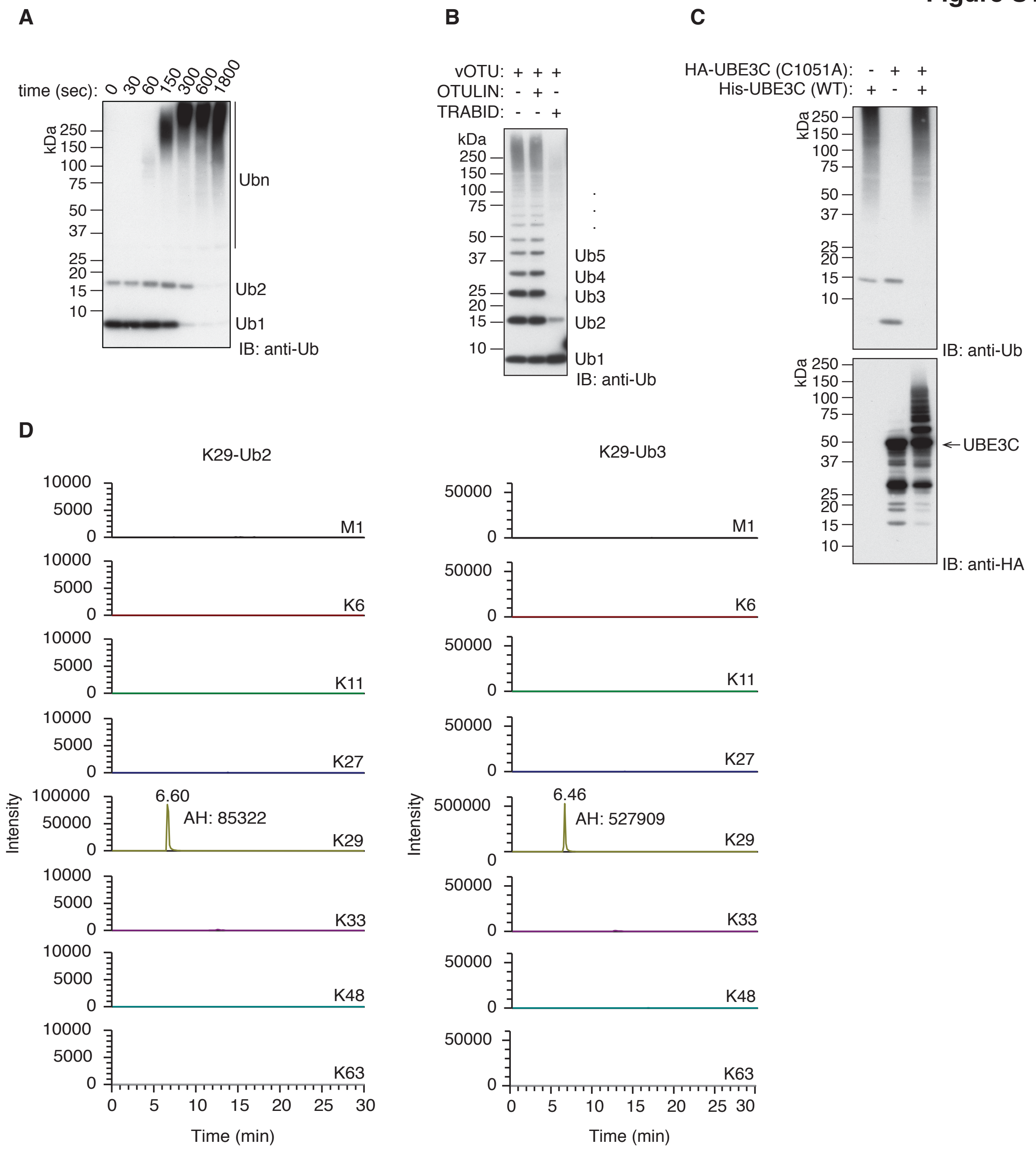
A
time (sec): 0 30 60 150 300 600 1800
kDa
250
150
100
75
50
37
25
20
15
10
Ubn
Ub2
Ub1
IB: anti-Ub
B
vOTU: + + +
OTULIN: - + -
TRABID: - - +
Ub5
Ub4
Ub3
Ub2
Ub1
IB: anti-Ub
C
HA-UBE3C (C1051A): - + +
His-UBE3C (WT): + - +
IB: anti-Ub
UBE3C
IB: anti-HA
D
K29-Ub2
K29-Ub3
M1
K6
K11
K27
K29
K33
K48
K63
Intensity
6.60
AH: 85322
6.46
AH: 527909
Time (min)

Figure S1. Polyubiquitin chains assembled using UBE3C and vOTU are K29-linked, Related to Figure 1.

(**A**) Ubiquitylation kinetics of UBE3C assayed using *in vitro* ubiquitylation reactions containing UBE1, UBE2D3, UBE3C, and Ub were incubated for the indicated time periods.

(**B**) Ubiquitylation assays of UBE3C containing UBE1, UBE2D3, UBE3C and Ub were performed in the presence of vOTU, M1 linkages-specific OTULIN, and K29 linkages-specific TRABID as indicated. The vOTU resistant chains are not hydrolyzed by OTULIN but are cleaved by TRABID.

(**C**) UBE3C is ubiquitylated in *trans.* Ubiquitylation reactions were performed using His tagged UBE3C wild type or HA-UBE3C C1051A or containing both together. Ubiquitylation of catalytically dead UBE3C C1051A was visualized by anti-HA immunoblotting (bottom panel).

(**D**) Parallel Reaction Monitoring (pRM) analysis of seven different Ub linkages in the purified K29-diUb and triUb (see **Figure 1F**). To highlight the purity of the K29-linkage and the absence of other linkages, the intensity scale for the non-K29 linkages was set 10-fold lower. AH: refers to automated height as determined by the XCalibur software.

Figure S2

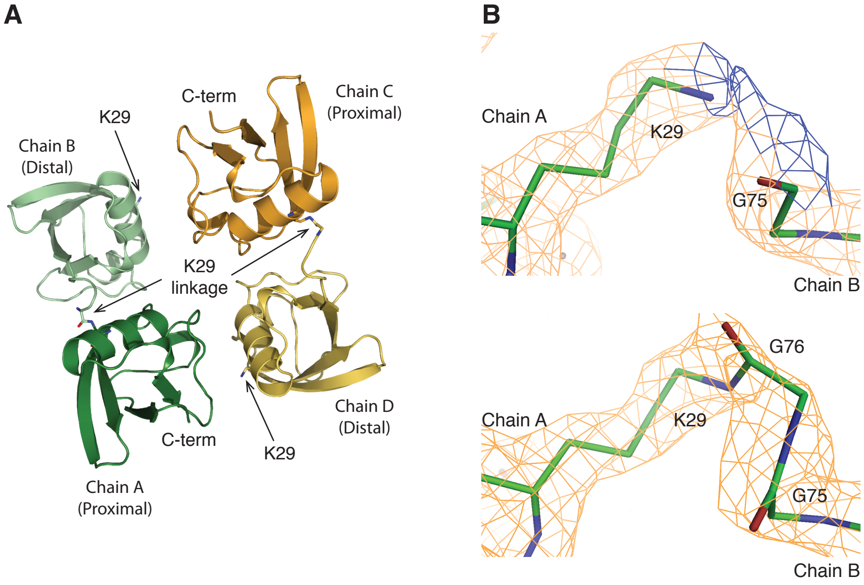

Figure S2. Structure of K29-linked diUb, Related to Figure 2.

(**A**) The asymmetric unit contains four Ub molecules and makes up two K29-linked dimers. Cartoon representation of one dimer (Chain A and B) is colored in shades of green and the other dimer (Chain C and D) is colored in shades of orange. The overall arrangement of the distal and proximal Ub moieties in both K29-linked diUb molecules present in the ASU are similar and both dimers adopt the same conformation.

(**B**) Electron density for the K29 linkage in the diUb structure. Electron density maps (orange represents $2|F_o|-|F_c|$ contoured at 0.7σ; blue represents $|F_o|-|F_c|$ contoured at 3σ) spanning K29 of the proximal Ub and the C-terminus of Ub before (top) and after (bottom) refinement with the isopeptide linkage. The linker region containing the isopeptide linkage has high temperature factors indicative of a high degree of flexibility.

Figure S3

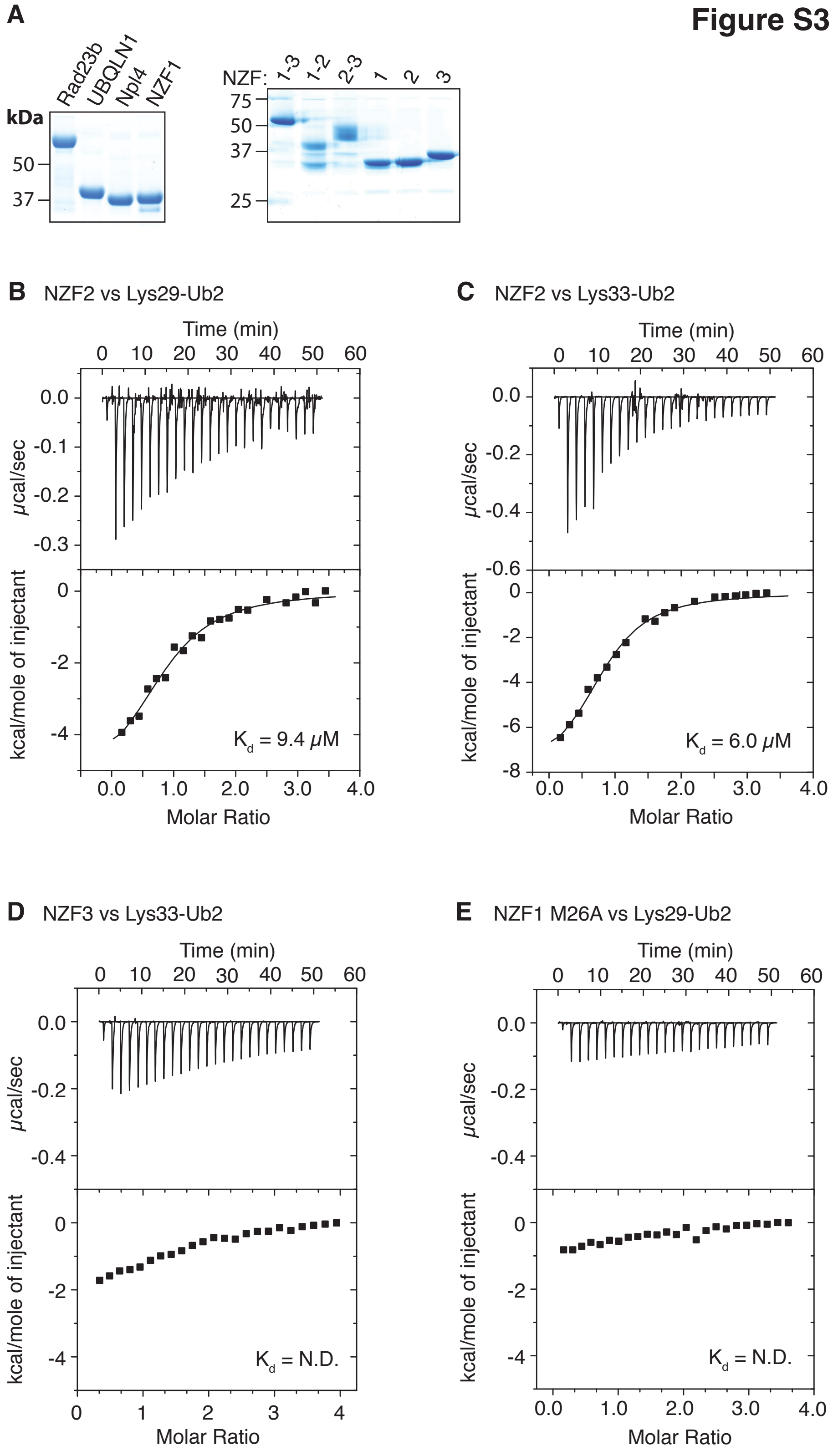
A
Rad23b
UBQLN1
Npl4
NZF1
kDa
50
37
NZF:
1-3
1-2
2-3
1
2
3
75
50
37
25
B NZF2 vs Lys29-Ub2
Time (min)
μcal/sec
kcal/mole of injectant
Kd = 9.4 μM
Molar Ratio
C NZF2 vs Lys33-Ub2
Time (min)
μcal/sec
kcal/mole of injectant
Kd = 6.0 μM
Molar Ratio
D NZF3 vs Lys33-Ub2
Time (min)
μcal/sec
kcal/mole of injectant
Kd = N.D.
Molar Ratio
E NZF1 M26A vs Lys29-Ub2
Time (min)
μcal/sec
kcal/mole of injectant
Kd = N.D.
Molar Ratio

Figure S3. Linkage specificity analysis of TRABID NZF domains, Related to Figure 3.

(**A**) Input proteins (1%) that were coupled to the HaloLink resin in Figure 3B and 3C were visualized on Coomassie-stained SDS-PAGE gel.

(**B-C**) Isothermal titration calorimetry (ITC) measurements for the NZF2 domain of TRABID with K29-Ub2 (B) and K33-Ub2 (C).

(**D**) ITC measurement for NZF3 domain of TRABID with K33-Ub2.

(**E**) ITC measurement for NZF1 M26A mutant of TRABID with K29-Ub2. The K_d value for each measurement is indicated.

Figure S4

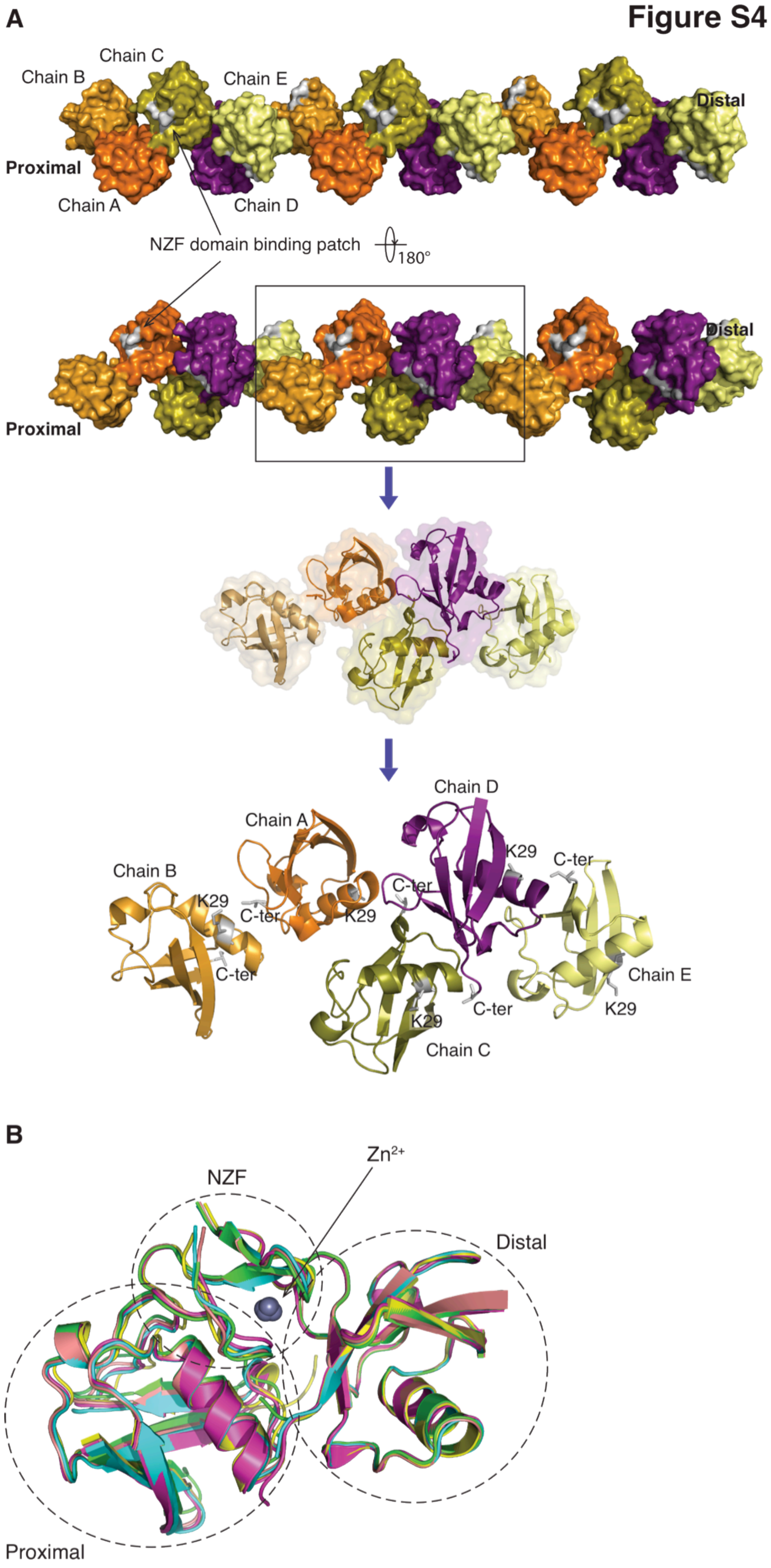
A
Chain B
Chain C
Chain E
Distal
Proximal
Chain A
Chain D
NZF domain binding patch
180°
Distal
Proximal
Chain D
Chain A
K29
C-ter
Chain B
K29
C-ter
C-ter
K29
C-ter
Chain E
C-ter
K29
K29
Chain C
B
Zn2+
NZF
Distal
Proximal

Figure S4. K29-linked chains form a helical filament like structure, Related to Figure 4. (**A**) The asymmetric unit (ASU) of the complex, contains five Ub moieties (chain A, B, C, D, E) and five NZF domains (chain F, G, H, I, J) (top panel). Symmetry related molecules of the ASU are expanded and the orientation of K29 of each Ub moiety and the C-terminal of the adjacent Ub moiety are shown (bottom panel). (**B**) Superposition of all NZF1-K29-diUb complexes within the ASU. The rmsd values range between 0.363 and 0.883 Å.

Figure S5

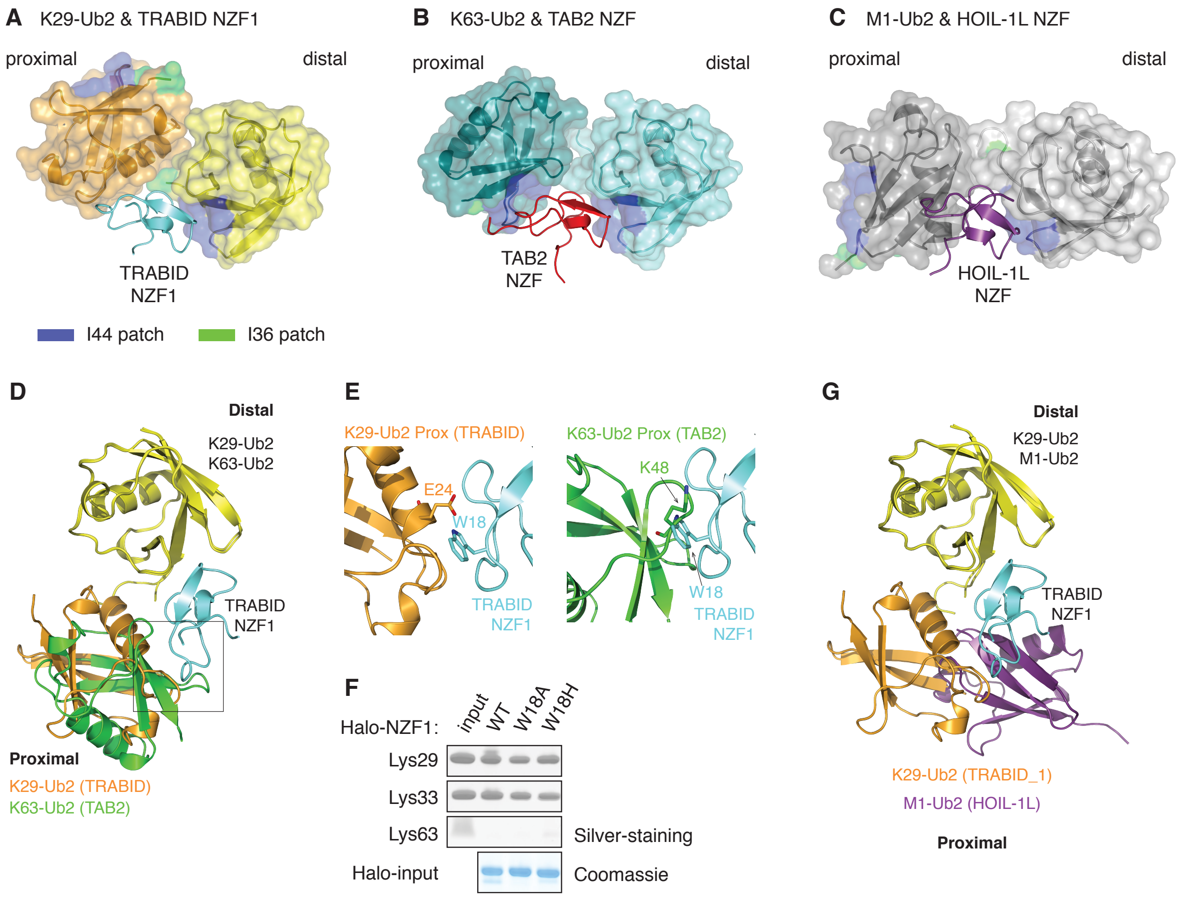
A K29-Ub2 & TRABID NZF1
proximal
distal
TRABID NZF1
I44 patch
I36 patch
B K63-Ub2 & TAB2 NZF
proximal
distal
TAB2 NZF
C M1-Ub2 & HOIL-1L NZF
proximal
distal
HOIL-1L NZF
D
Distal
K29-Ub2
K63-Ub2
TRABID NZF1
Proximal
K29-Ub2 (TRABID)
K63-Ub2 (TAB2)
E
K29-Ub2 Prox (TRABID)
E24
W18
TRABID NZF1
K63-Ub2 Prox (TAB2)
K48
W18
TRABID NZF1
F
Halo-NZF1:
input
WT
W18A
W18H
Lys29
Lys33
Lys63
Silver-staining
Halo-input
Coomassie
G
Distal
K29-Ub2
M1-Ub2
TRABID NZF1
K29-Ub2 (TRABID_1)
M1-Ub2 (HOIL-1L)
Proximal

Figure S5. Two-sided Ub-binding mode of NZF domains, Related to Figure 5.

(**A-C**) A semitransparent surface of diUb and NZF domains in cartoon representative of K29-Ub2 and TRABID NZF1 (A), K63-Ub2 and TAB2 NZF (B), and M1-Ub2 and HOIL-1L NZF (C). Residues comprising I44 patch (I44, L8, H68, and V70) and I36 patch (I36, L71, and L73) are colored blue and green, respectively.

(**D**) Superposition of the distal Ub moieties (yellow) of K29-Ub2-TRABID NZF1 (orange) and K63-Ub2-TAB2 (green). For clarity, only the NZF1 of TRABID is shown (cyan).

(**E**) Zoom-in of the boxed region in (D) was reoriented to show the position of W18 of TRABID NZF1 in proximity of E24 on the proximal Ub of K29-diUb (top) and steric clashes of TRABID NZF1 with the proximal Ub of the superposed K63-diUb (bottom).

(**F**) Mutating W18 residue of NZF1 does not increase binding to K63-Ub2. Halo-tagged NZF1 wild type or mutants were used in pull-down assay with K29-, K33-, and K63-Ub2. The captured chains were visualized by silver-staining. 50% of tetraUb input used in the pull down assay was included as control.

(**G**) Superposition of the distal Ub moieties (yellow) of K29-Ub2-TRABID NZF1 (orange) and Met1-Ub2-HOIL-1L (purple). For clarity, only the NZF1 of TRABID is shown (cyan). PDB ID: 2WWZ, (Kulathu et al., 2009); 3B08, (Sato et al., 2011).

Figure S6

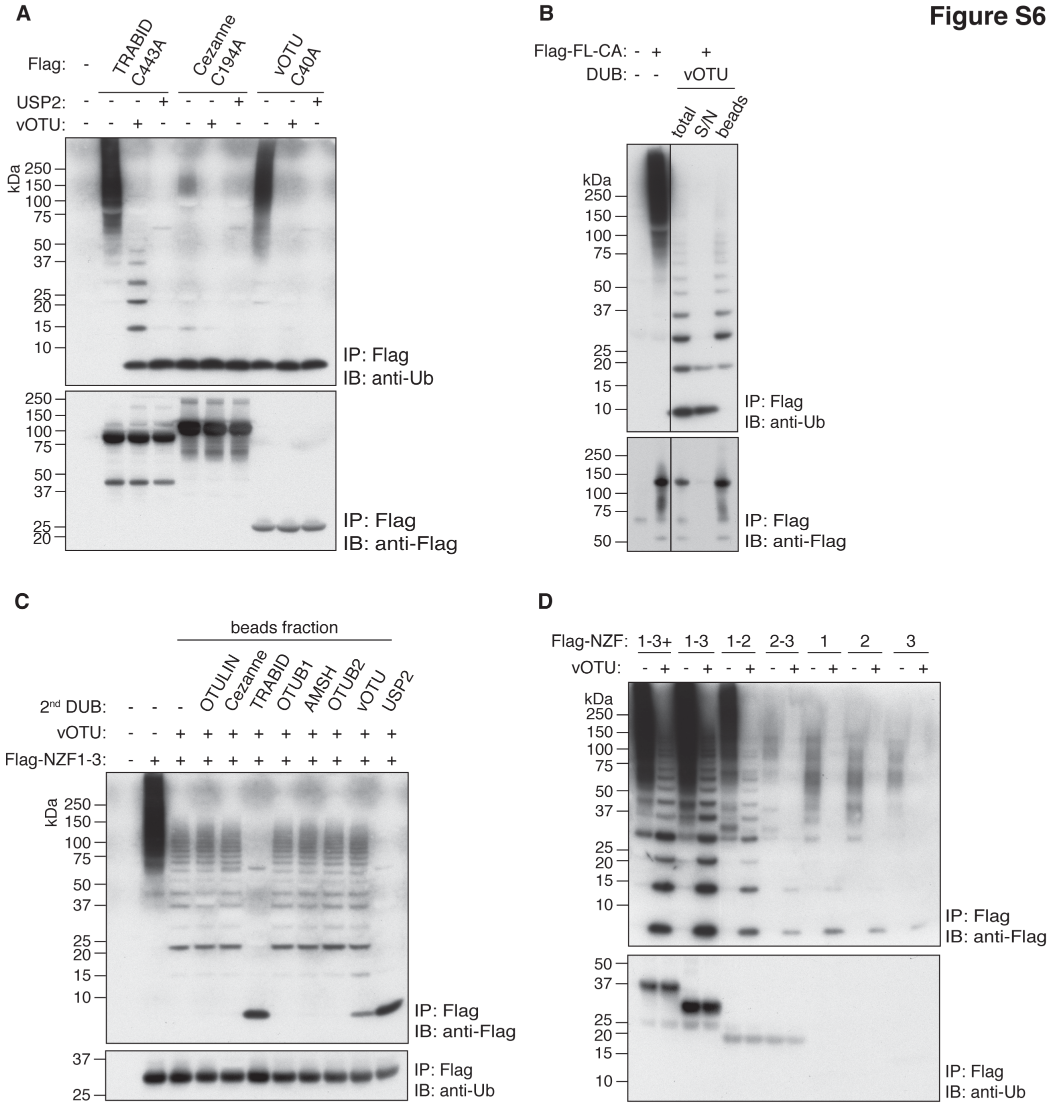

A
Flag:
TRABID C443A
Cezanne C194A
vOTU C40A
USP2:
vOTU:
kDa
250
150
100
75
50
37
25
20
15
10
IP: Flag
IB: anti-Ub
IP: Flag
IB: anti-Flag
B
Flag-FL-CA:
DUB:
vOTU
total
S/N
beads
IP: Flag
IB: anti-Ub
IP: Flag
IB: anti-Flag
C
beads fraction
2nd DUB:
vOTU:
Flag-NZF1-3:
OTULIN
Cezanne
TRABID
OTUB1
AMSH
OTUB2
vOTU
USP2
IP: Flag
IB: anti-Flag
IP: Flag
IB: anti-Ub
D
Flag-NZF:
1-3+
1-3
1-2
2-3
1
2
3
vOTU:
IP: Flag
IB: anti-Flag
IP: Flag
IB: anti-Ub

Figure S6. The tandem NZF1-3 domain of TRABID captures polyUb chains containing K29 linkages from HEK293 cells, Related to Figure 6.

(**A**) Flag-tagged catalytic dead TRABID (C443A), Cezanne (C194A), and vOTU (C40A) were transiently expressed in HEK293 cells. The Flag-fusion proteins were immunoprecipitated and the captured polyUb material was incubated with vOTU to determine if K29 linkages are present. Short chains are released from TRABID isolates but not from vOTU or Cezanne pull-downs.

(**B**) K29 chains are present in the bead fraction bound to TRABID. Flag-tagged full-length catalytic dead TRABID (FL-CA) was transiently expressed in HEK293 cells. The Flag-fusion proteins were immunoprecipitated and the captured polyUb material was incubated with vOTU. The ubiquitin content of the supernatant and bead fraction were analyzed by anti-Ub immunoblotting.

(**C**) Flag-tagged TRABID NZF1-3 was immunoprecipitated from transfected HEK293 cells and incubated with vOTU. The bead fraction containing vOTU resistant chains was assayed for its Ub linkage content by incubation with DUBs that exhibit preference for cleaving different linkages.

(**D**) The indicated Flag-tagged TRABID constructs were transiently expressed in HEK293 cells, immunoprecipitated using Flag-M2 beads, and the captured polyUb material was treated with vOTU. Ectopic TRABID NZF expression was visualized by anti-FLAG immunoblotting.

Figure S7

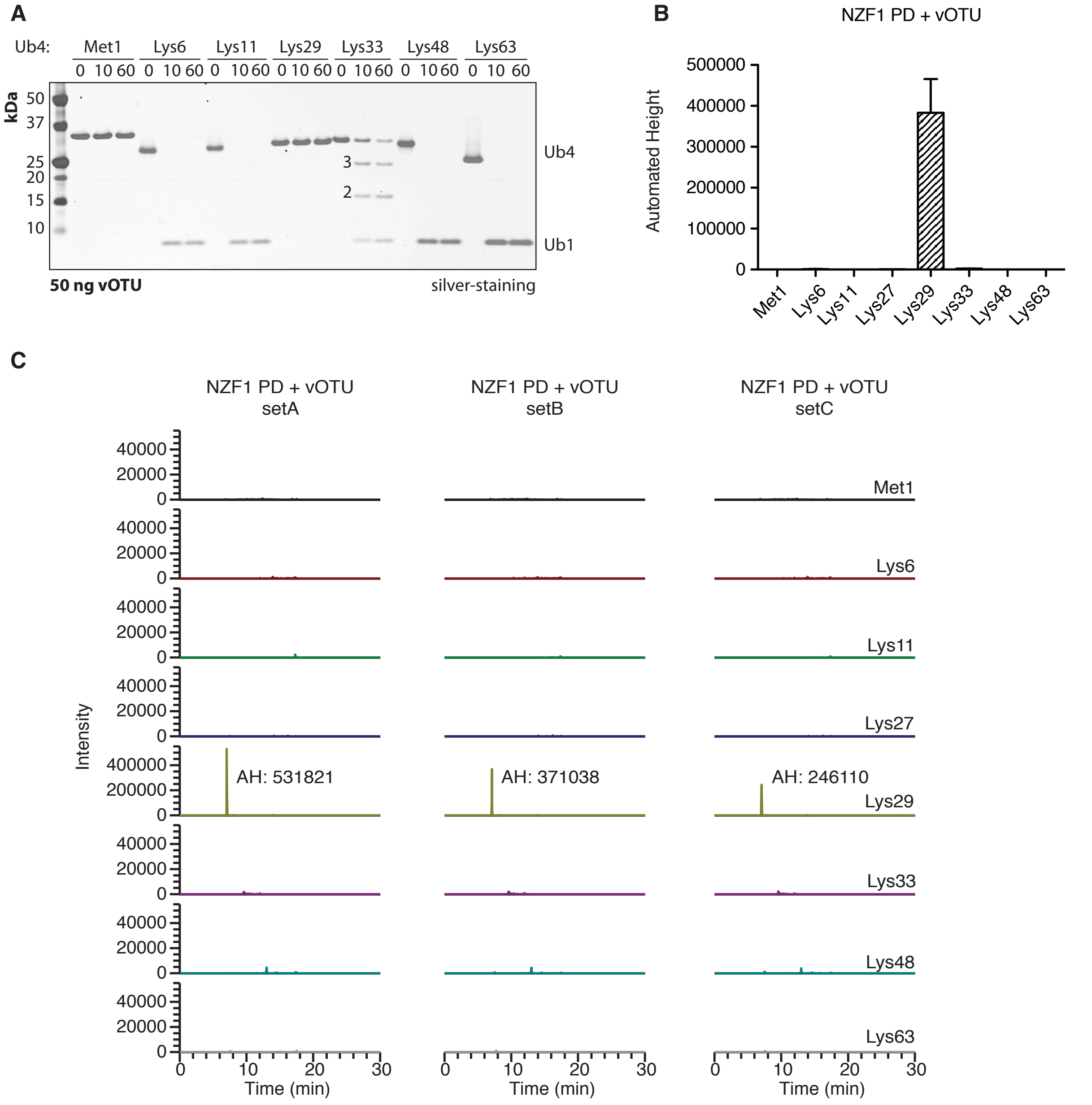
A
Ub4:
Met1
Lys6
Lys11
Lys29
Lys33
Lys48
Lys63
0 10 60 0 10 60 0 10 60 0 10 60 0 10 60 0 10 60 0 10 60
kDa
50
37
25
20
15
10
3
2
Ub4
Ub1
50 ng vOTU
silver-staining
B
NZF1 PD + vOTU
Automated Height
500000
400000
300000
200000
100000
0
Met1
Lys6
Lys11
Lys27
Lys29
Lys33
Lys48
Lys63
C
NZF1 PD + vOTU
setA
NZF1 PD + vOTU
setB
NZF1 PD + vOTU
setC
Intensity
40000
20000
0
400000
200000
Met1
Lys6
Lys11
Lys27
Lys29
Lys33
Lys48
Lys63
AH: 531821
AH: 371038
AH: 246110
0
10
20
30
Time (min)

Figure S7. Verification that the linkage type present in the vOTU-resistant polyUb chains is K29, Related to Figure 6.

(**A**) Deubiquitinase reactions were carried out in 10 µl reaction of 50 ng vOTU and 0.5 µg tetraUb of seven linkages at 30 °C for the indicated time. Reaction was quenched by adding LDS sample buffer and analyzed on silver-stained 4-12% SDS-PAGE gel.

(**B**) PolyUb chains captured from HEK293 cells using Halo-NZF1 were treated with vOTU. The abundance of Ub linkages of the vOTU-resistant chains that were still captured on the resin was analyzed by parallel reaction monitoring (pRM) as in Figure S1D. The error bar represents the standard error of the mean of three measurements.

(**C**) The extracted-ion chromatography of vOTU-resistant chains analyzed by pRM in (B). To highlight the abundance of the K29-linkage over other linkages, the intensity scale for the non-K29 linkages was set 10-fold lower. AH: refers to automated height as determined by the XCalibur software.

Figure S8

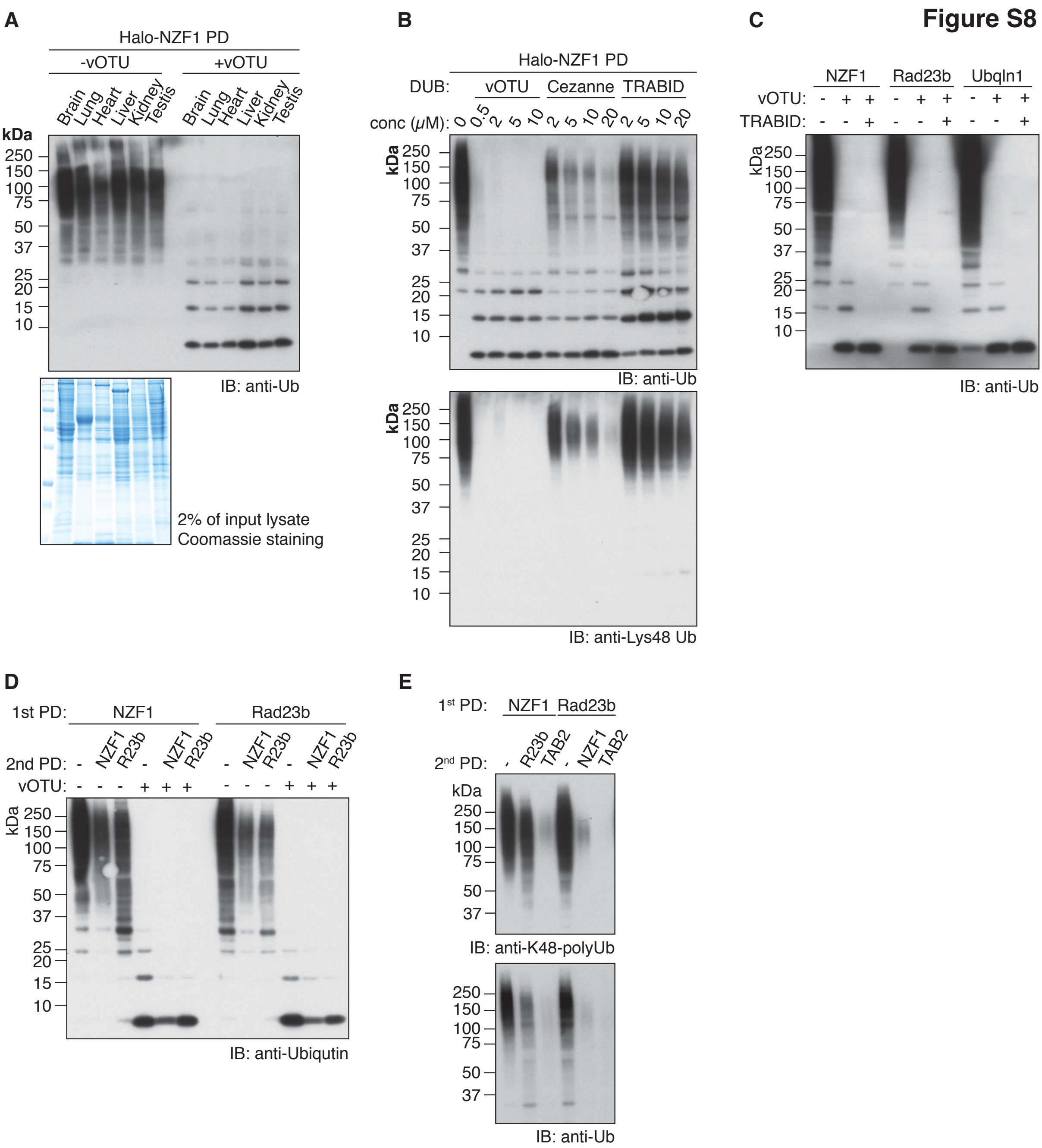
A
Halo-NZF1 PD
-vOTU
+vOTU
Brain Lung Heart Liver Kidney Testis
Brain Lung Heart Liver Kidney Testis
kDa
250
150
100
75
50
37
25
20
15
10
IB: anti-Ub
2% of input lysate
Coomassie staining
B
Halo-NZF1 PD
DUB:
vOTU
Cezanne
TRABID
conc (μM): 0 0.5 2 5 10 2 5 10 20 2 5 10 20
kDa
IB: anti-Ub
IB: anti-Lys48 Ub
C
NZF1
Rad23b
Ubqln1
vOTU:
TRABID:
kDa
IB: anti-Ub
D
1st PD:
NZF1
Rad23b
2nd PD:
NZF1
R23b
vOTU:
kDa
IB: anti-Ubiqutin
E
1st PD:
NZF1
Rad23b
2nd PD:
R23b
TAB2
NZF1
TAB2
kDa
IB: anti-K48-polyUb
IB: anti-Ub

Figure S8. K29-linked chains are present within mixed or branched heterotypic chains, Related to Figure 7.

(**A**) PolyUb chains from the indicated mouse tissues were captured using Halo-TRABID NZF1 and the presence of K29 chains was assayed by treating one half of the samples with vOTU. For a loading control, 2% of input lysate for IP was separated and visualized on Coomassie-stained SDS-PAGE gel.

(**B**) PolyUb chains from HEK293 were captured using Halo-TRABID NZF1 and incubated with an increasing concentration of vOTU, Cezanne, and TRABID for 1 h, 2 h, and 2 h, respectively. The K48-linked polyUb chains were analyzed using anti-K48 Ub linkage.

(**C**) PolyUb materials from HEK293 cells captured by Halo-TRABID NZF1, Halo-Rad23b UBA1-2, and Halo-Ubiquilin1 UBA were treated with vOTU and TRABID as indicated.

(**D**) PolyUb materials from HEK293 cells were captured by Halo-TRABID NZF1 or Halo-Rad23b, and where indicated, the supernatants from the first pull-down were subjected to a second pull-down. The presence of K29 linkages was assayed by incubating one half of the pull-down material with vOTU.

(**E**) As in (D), except the captured polyUb species were not treated with vOTU. The presence of K48 linkages in the pulled-down polyUb material was visualized using anti-K48 polyUb immunoblotting.

Table S1. cDNA constructs used in this study, Related to Figure 1, 3-7, S1, S3, S5-S8.

Protein	Tag	Accession No	Construct Boundaries	Expression system	Plasmid	DU number
UBE3C	GST-cleaved	Q15386	641-1083	insect	pFastbac	45301
vOTU	GST-cleaved	3ZNH_A	1-183	bacterial	pGEX6P	45351
TRABID NZF1	GST-cleaved	Q9UGI0	3-33	bacterial	pGEX6P	23225
TRABID NZF1-3	GST-cleaved, Halo	Q9UGI0	3-178	bacterial	pGEX6P	24214
TRABID NZF1-2	GST-cleaved, Halo	Q9UGI0	3-110	bacterial	pGEX6P	49556
TRABID NZF2-3	GST-cleaved, Halo	Q9UGI0	82-187	bacterial	pGEX6P	24485
TRABID NZF1	GST-cleaved, Halo	Q9UGI0	1-33	bacterial	pGEX6P	24486
TRABID NZF2	GST-cleaved, Halo	Q9UGI0	82-113	bacterial	pGEX6P	24487
TRABID NZF3	GST-cleaved, Halo	Q9UGI0	139-187	bacterial	pGEX6P	24488
Npl4 NZF	GST-cleaved, Halo	Q8TAT6	575-608	bacterial	pGEX6P	49635
Ubiquilin1 UBA	GST-cleaved, Halo	Q9UMX0	539-587	bacterial	pGEX6P	49634
Rad23b UBA1-2	GST-cleaved, Halo	P54727	186-407	bacterial	pGEX6P	49626
TAB2	GST-cleaved, Halo	Q9NYJ8	663-693	bacterial	pGEX6P	49635
TRABID FL-WT	3xFlag	Q9UGI0	full length	mammalian	pcDNA5	49067
TRABID FL-C443A	3xFlag	Q9UGI0	full length	mammalian	pcDNA5	49089
TRABID NZF1-3	3xFlag	Q9UGI0	1-187	mammalian	pcDNA5	24389
Cezanne C194A	3xFlag	Q6GQQ9	full length	mammalian	pcDNA5	49193
vOTU C40A	3xFlag	3ZNH_A	1-183	mammalian	pcDNA5	49177
TRABID NZF1-3+	3xFlag	Q9UGI0	1-245	mammalian	pcDNA5	49140
TRABID NZF1-2	3xFlag	Q9UGI0	1-113	mammalian	pcDNA5	24391
TRABID NZF2-3	3xFlag	Q9UGI0	82-187	mammalian	pcDNA5	24390
TRABID NZF1	3xFlag	Q9UGI0	1-33	mammalian	pcDNA5	24394
TRABID NZF2	3xFlag	Q9UGI0	82-113	mammalian	pcDNA5	24393
TRABID NZF3	3xFlag	Q9UGI0	139-187	mammalian	pcDNA5	24392

Table S2. Parameter used in the parallel Reaction Monitoring (pRM) analysis, Related to Figure S1 and S7.

Linkage	Peptide Sequence	Charge	Retention Time	Precursor (m/z)	Daughter masses used for quantification
M1	M(gg)QIFVK	2+	8.7	440.34	y_3, 393.25; y_4, 506.33; y_5, 634.39; b_7, 733.37
K6	MQIFVK(gg)TLTGK	2+	13.6	690.50	y_6, 761.45; y_7, 860.50; y_8, 1007.59; y_9, 1120.67
K11	TLTGK(gg)TITLEVEPSDTIENVK	3+	14.6	801.84	y_8, 905.46; y_9 1002.51; y_{10}, 1131.55
K27	TITLEVEPSDTIENVK(gg)AK	2+	13.9	1051.56	y_8, 1016.57; y_{11}, 1315.68; y_{12}, 1444.73; y_{13}, 1543.79
K29	AK(gg)IQDK	2+	6.6	408.80	b_3, 427.26; y_4, 503.30; b_5, 670.35; y_5, 745.40
K33	IQDK(gg)EGIPPDQQR	3+	9.3	546.61	y_6^{2+}, 370.90; y_6, 740.30; b_7, 898.30
K48	LIFAGK(gg)QLEDGR	2+	12.7	731.36	y_4, 476.40; y_5, 589.45; y_6, 717.20
K63	TLSDYNIQK(gg)ESTLHLVLR	2+	14.5	1122.67	y_5, 637.41; y_8,938.58; y_9, 1067.62

Table S3. Summary of interactions in K29-linked diUb – TRABID NZF1 complex, Related to Figure 4.

Distal Ub with NZF

Ub^{dist}	NZF	Interaction
Leu8	Tyr12	Hydrophobic
Ile44	Tyr15	Hydophobic
Ile44	Met26	Hydophobic
Val70	Met26	Hydophobic
Gly47	Glu16	Hydophobic
Gln49	Met26	Hydrogen

Proximal Ub with NZF

Ub^{prox}	NZF	Interaction
Ala28	Met26	Hydophobic
Glu24	Tyr15	Hydrogen
Glu24	Thr25	Hydrogen

Distal Ub with Proximal Ub

Ub^{dist}	Ub^{prox}	Interaction
Arg42	Asp32	Ionic, Hydrogen
Gln49	Gln31	Hydrogen
Leu73	Asp32	Hydrogen
Arg72	Asp32	Ionic

Table S4. K_d values of TRABID NZF domain binding to polyubiquitin, Related to Figure 3 and S3.

	K29-Ub2	K33-Ub2
NZF1	3.0	4.2
NZF2	9.4	6.0
NZF3	-	N.D.
NZF1 M26A	N.D.	-

Values (in μM) were determined by ITC.
ND, no detectable binding

Extended Experimental Procedures

Plasmids and antibodies. All cDNA constructs for bacterial, insect, and mammalian expression system were generated by the DNA cloning team, Division of Signal transduction Therapy, Medical Research Council Protein Phosphorylation and Ubiquitylation Unit, University of Dundee, United Kingdom (**Table S1**). Recombinant proteins and plasmids generated for the present study are available on our reagents website (https://mrcppureagents.dundee.ac.uk/). Anti-ubiquitin to detect *in vitro* ubiquitylation and polyUb chains from cells were purchased from SIGMA (U5379) and DAKO (Z0458), respectively. Anti-Flag was from SIGMA (F3165) and anti-HA was from Cell Signaling Technology (#3724).

Protein expression and purification. For bacterial expression, recombinant GST-fusion proteins were expressed in BL21 (DE3) *E. coli* cells. Cultures were grown in 2xTY media to OD_{600} of 0.6-0.8 and the protein expression was induced by adding 300 μM IPTG and further incubation at 16 °C overnight. Medium was supplemented with 200 μM $ZnCl_2$ for expression of NZF domains. Cells were lysed by sonication in GST-Lysis Buffer (50 mM Tris-HCl (pH 7.5), 300 mM NaCl, 10% glycerol, 0.075% 2-mercaptoethanol, 1 mM benzamidine, 1 mM AEBSF, and complete protease inhibitor cocktail (Roche)). Bacterial lysate was clarified by centrifugation and incubated subsequently with Glutathione Sepharose 4B resin (GE Healthcare) for 2 h at 4 °C. Resins were washed extensively with high salt buffer (250 mM Tris (pH 7.5), 500 mM NaCl, and 5 mM DTT) and low salt buffer (25 mM Tris (pH 7.5), 150 mM NaCl, 10% glycerol, and 1 mM DTT). Recombinant proteins were eluted from the resin by cleaving the GST-tag using GST-tagged C3 protease.

For insect cell protein expression, recombinant GST-fusion UBE3C (641-1083) was expressed in Sf21 cells using Bac-to-Bac baculovirus expression system (Invitrogen). Sf21 cells cultured at 27 °C in Insect Xpress medium (Lonza) supplemented with Antibiotic-Antimycotic (Invitrogen) were infected with P1 virus stocks and harvested 60 h later. Cells were lysed in Lysis Buffer (50 mM Tris-HCl (pH 7.5), 5% glycerol, 0.1 M EDTA, 0.1 mM EGTA, 1 mM DTT, 1 mM Pefabloc, and 20 μg/ml Leupeptin) using Dounce homogenizer, then centrifuged to remove insoluble material. Total concentration of 250 mM NaCl was added to

the lysate prior to a one-hour incubation with Glutathione agarose (Expedeon). Resins were washed in wash buffer (50 mM Tris (pH 7.5), 500 mM NaCl, 5% glycerol, 1 mM DTT) and then cleavage buffer (50 mM Tris-HCl (pH 7.5), 150 mM NaCl, 1 mM DTT). Recombinant proteins were eluted from the resin by cleaving the GST-tag off using C3 protease.

Ubiquitylation assays. Analytical assays were carried out in 20-μl reactions at 30 °C containing 250 nM UBE1, 2.25 μM E2 (UBE2D1, D2, D3, or L3), 1.56 μM UBE3C, 57 μM Ub, 10 mM ATP, 50 mM Tris-HCl (pH 7.5), 10 mM $MgCl_2$, and 0.6 mM DTT. After 3 h, 2 μM vOTU was added and the reaction was continued for 2 h. The reaction was stopped by addition of 10 μl 4 x LDS sample buffer (Invitrogen), resolved by SDS-PAGE on 4-12% gradient gels (Invitrogen) and subjected to western blot analysis using rabbit polyclonal anti-Ub antibody (SIGMA).

Purification of polyubiquitin chains. Following large-scale assembly of K29-linked polyUb chain, enzymes used in the reaction were precipitated by diluting the Ub chains in a total volume of 50 ml of 50 mM sodium acetate (pH 4.5). After at least 3 h incubation at 4 °C, the solution was passed through a 0.22-μM syringe filter and the K29-linked diUb, triUb, tetraUb, and pentaUb were purified by cation exchange using a Resource S 6 ml column (GE Healthcare), equilibrated in 50 mM sodium acetate (pH 4.5), and eluted in a gradient with elution buffer (50 mM sodium acetate (pH 4.5), 1 M NaCl). For crystallization, peak fractions containing K29-linked diUb were concentrated to 12 mg/ml.

Analysis of polyubiquitin linkages by parallel Reaction Monitoring (pRM) using LC-MS-MS. PolyUb chains that had been previously digested with trypsin were analyzed on an LTQ-Velos mass spectrometer (Thermo) fitted with a Dionex RSLC HPLC system and an Easy-Spray Source (Thermo). Standard diUb chains were purchased from Boston Biochemicals and a synthetic peptide AK(GG)IQDK representing the tryptic Ub K29 linkage was purchased from Pepceuticals (Nottingham, UK). Digests (prepared in 0.1% TFA/water) were loaded onto a 20 x 0.1 mm nanotrap column (Thermo) equilibrated in 0.1% TFA/water (10 μl/min), washed with 10 μl of the same buffer, and then separated on a 150 x 0.075 mm PepMap C18, 3 μm Easy-Spray column (Thermo)

equilibrated with 2% acetonitrile/ 0.1% formic acid/ water at 300 nl/min. It was critical that the samples were loaded and washed in TFA buffers, as the trap column in the presence of formic acid did not retain the tryptic peptide containing the K29 linkage. Peptides were separated at the same flow rate using a discontinuous gradient of buffer B (80% acetonitrile/ 0.1% formic acid/ water) as follows: 0-14 min = 1-30% B, 14-15 min = 30-80% B, 15-20 min = 80% B. LC-MS data was acquired in Data Independent mode with 1 full scan (m/z 350-1800) followed by 8 product ion scans as described below. The voltage applied to the Easy-Spray column was 1.9 kV, the isolation width was set to 1 Da, normalised collision energy was 35 and the activation time was 10 ms. Xcalibur software (Thermo) was used to process the data, with the ion current for the daughter ions being summed for each precursor mass analyzed (**Table S2**). The resultant summed intensities provide the y-axis values for **Figure S1D** and **Figure S7C**. This was a much cleaner and selective analysis method rather than using the extracted ion current for the precursor mass for each ubiquitin chain peptide.

Crystallization and structure determination. Purified K29-linked diUb chains were crystallized at 12 mg/ml in mother liquor containing 100 mM Bis-Tris propane (pH 6.5), 200 mM sodium iodide, 20% PEG3350, 5% ethylene glycol and 5 mM sodium malonate at 20 °C. Further, seeding technique was used to obtain diffraction quality crystals. The single crystals obtained were cryo-protected in mother liquor containing 20% glycerol and 20% PEG400 before freezing in liquid nitrogen.

For crystallization of the complex, TRABID NZF1 and K29 diUb were mixed together in molar ratio of 1:1 and incubated for 3 hours at 4 °C before concentration using Millipore concentrator (MW cut off 3kDa). Crystallization screening was set up using protein complex concentrated to 18 mg/ml and crystallization trays incubated at 12 °C. The complex crystallized in mother liquor containing 100 mM MES (pH 6.5), 200 mM potassium iodide, and 25% PEG4000. The crystals grew to maximum size in one week and were cryo-protected in 100 mM MES (pH 6.5), 10% PEG20000, and 35% PEG400 before vitrification in liquid nitrogen.

Diffraction data for K29 diUb and the NZF1-K29 diUb complex were collected at ESRF beam line ID23-1 and Diamond beam line I04, respectively. All data were processed using XDS (Kabsch, 2010) and scaled using SCALA (Evans, 2006). The structures were solved by molecular replacement using the structures of ubiquitin (1UBQ (Vijay-Kumar et al., 1987)) and the NZF domain of TAB2 (2WWZ (Kulathu et al., 2009)) as search models in Phaser (McCoy et al., 2005). Iterative rounds of refinement was done using Phenix (Adams et al., 2002) and REFMAC (Murshudov et al., 1997) with model building in Coot (Emsley and Cowtan, 2004). Simulated annealing and rigid body refinement was used in the initial stages to remove model bias. Final re-refinement for K29 diUb was done using PDB_REDO (Joosten et al., 2014). Both structures were refined to the final statistics as shown in **Table 1**.

Isothermal Titration Calorimetry. ITC titration were performed on a MicroCal™ iTC_{200} at 25 °C in ITC buffer (50 mM HEPES (pH 7.5), 150 mM NaCl, 250 μM TCEP). Each titration used 25 × 1.5 μl injections. For all titrations, the syringe contained the NZF proteins and the cell contained diUb. Concentration of 300 μM and 20 μM was used for titrations of NZF1 and diUb, respectively. For titration of NZF2 and diUb, concentration of 550 μM and 30 μM was used, respectively. For titration of NZF3 (800 μM) and K33-Ub2 (40 μM), and NZF1 M26A (550 μM) and K29-Ub2 (30 μM), higher concentration of proteins was used to increase the ITC signal.

Assembly and purification of tetraubiquitin chains. K6-linked chains were enzymatically assembled using wild type Ub, UBE2L3 and NleL as described before (Hospenthal et al., 2013). K11-linked tetraUb was purified from ubiquitylation products generated by UBE2S (1-192) and contaminating K63 chains were removed by incubation with AMSH (Bremm et al., 2010). K48- and K63-linked polyUb chains were assembled as described using UBE2R1 and UBE2N/UBE2V1 respectively (Kulathu et al., 2009), while HOIP was used for assembly for M1-linked chains (Stieglitz et al., 2012). K33-linked polyUb chains were assembled as described (Kristariyanto, et al., manuscript submitted). K29-linked polyUb chains were assembled as described above. For each linkage type, polyUb of defined lengths were separated by cation exchange chromatography

and the fractions containing tetraUb were concentrated and dialyzed into 50mM Tris, pH7.5 buffer.

Cell culture, transfection, and cell lysis. HEK293 cells were maintained in DMEM supplemented with 10% (v/v) FBS, 2 mM L-glutamine and antibiotics (100 units/ml penicillin, 0.1 mg/ml streptomycin) and were cultured at 37 °C in a 10% CO2 humidified atmosphere. All 3xFlag-tagged proteins in mammalian expressing vector (**Table S1**) were introduced to HEK293 cells using polyethylenimine (PEI) (Bioscience). Once the cells were confluent, they were harvested in PBS, pelleted, snap frozen, and stored at -80 °C. The cells pellet was thawed and incubated in Lysis Buffer (20 mM HEPES (pH 7.5), 110 mM potassium acetate, 2 mM magnesium acetate, 1 mM EGTA, 1 mM sodium ortho-vanadate, 1 mM NaF, 0.1% NP-40, 1 mM ABSF, 25 mM iodoacetamide, 0.02% benzonase (SIGMA), protease inhibitor cocktail) for 30 min at 4 °C in the dark. Cells were lysed by two times freeze-and-thawing cycle and a final concentration of 150 mM NaCl and 5% glycerol was added. Cell lysates were clarified by centrifugation at 14000 x g for 20 min at 4 °C, supernatants were collected and protein concentrations were determined by the Bradford procedure.

Pull-down assays and deubiquitinase treatments. To isolate transiently expressed Flag-tagged proteins and the interacting polyUb chains from HEK293 cells, 1 mg of the transfected cell lysates was incubated with 10 µl of Flag-M2 resin (SIGMA) for 2 h at 4 °C. To isolate polyUb chains from HEK293 cells using bacterially expressed Halo-tagged UBDs, 1 mg of the cell lysates was incubated with 10 µl Halo-UBD resins for 2 h at 4 °C. The polyUb chains captured by 3xFlag- and Halo-tagged proteins were washed three times with Lysis Buffer containing 150 mM NaCl, and once with DUB buffer (50 mM Tris-HCl (pH 7.5), 150 mM NaCl, and 5 mM DTT). Following the last wash, the beads were resuspended in 2x resin volume of 2 µM vOTU for 1 h at 30 °C. When subsequent DUB assays were carried out after the first incubation with vOTU, the beads were first washed twice with DUB buffer and then incubated with 5 µM DUBs (OTULIN, Cezanne, TRABID, OTUB1, AMSH, OTUB2, vOTU, and USP2) in 2x resin volume for 1 h at 30 °C. Reaction was quenched by adding reducing LDS buffer (Invitrogen), and the polyUb chains were analyzed in by immunoblotting against anti-Ub (DAKO)

or anti-K48 Ub linkage (CST). The immunoprecipitated Flag-tagged proteins were visualized by anti-FLAG (SIGMA).

For the tetraUb pull-down assays, 10 µl of the Halo-UBDs resins were incubated with 1 µg tetraUb chains of of the indicated linkages (M1, K6, K11, K29, K33, K48, and K63 tetraUb) in 500 µl pull-down buffer (50 mMTris-HCl (pH 7.5), 150 mM NaCl, 0.1% NP-40, 5 mM DTT, 0.5 mg/ml BSA) for 2 h at 4 °C. Beads were washed three times in Wash Buffer (50 mM Tris-HCl (pH 7.5), 250 mM NaCl, 0.2% NP-40, and 5 mM DTT). Captured tetraUb chains were separated on 4-12% SDS-PAGE gel and visualized using silver-staining kit (Thermo Scientific).

Supplemental References

Adams, P.D., Grosse Kunstleve, R.W., Hung, L.-W., Ioerger, T.R., McCoy, A.J., Moriarty, N.W., Read, R.J., Sacchettini, J.C., Sauter, N.K., and Terwilliger, T.C. (2002). PHENIX: building new software for automated crystallographic structure determination. Acta Crystallogr. D Biol. Crystallogr. *58*, 1948–1954.

Bremm, A., Freund, S.M.V., and Komander, D. (2010). Lys11-linked ubiquitin chains adopt compact conformations and are preferentially hydrolyzed by the deubiquitinase Cezanne. Nat. Struct. Mol. Biol. *17*, 939–947.

Emsley, P., and Cowtan, K. (2004). Coot: model-building tools for molecular graphics. Acta Crystallogr. D Biol. Crystallogr. *60*, 2126–2132.

Evans, P. (2006). Scaling and assessment of data quality. Acta Crystallogr. D Biol. Crystallogr. *62*, 72–82.

Hospenthal, M.K., Freund, S.M.V., and Komander, D. (2013). Assembly, analysis and architecture of atypical ubiquitin chains. Nat. Struct. Mol. Biol. *20*, 555–565.

Joosten, R.P., Long, F., Murshudov, G.N., and Perrakis, A. (2014). The PDB_REDO server for macromolecular structure model optimization. IUCrJ *1(Pt 4)*, 213-220.

Kabsch, W. (2010). XDS. Acta Crystallogr. D Biol. Crystallogr. *66*, 125–132.

Kulathu, Y., Akutsu, M., Bremm, A., Hofmann, K., and Komander, D. (2009). Two-sided ubiquitin binding explains specificity of the TAB2 NZF domain. Nat. Struct. Mol. Biol. *16*, 1328–1330.

McCoy, A.J., Grosse Kunstleve, R.W., Storoni, L.C., and Read, R.J. (2005). Likelihood-enhanced fast translation functions. Acta Crystallogr. D Biol. Crystallogr. *61*, 458–464.

Murshudov, G.N., Vagin, A.A., and Dodson, E.J. (1997). Refinement of macromolecular structures by the maximum-likelihood method. Acta Crystallogr. D Biol. Crystallogr. *53*, 240–255.

Sato, Y., Fujita, H., Yoshikawa, A., Yamashita, M., Yamagata, A., Kaiser, S.E., Iwai, K., and Fukai, S. (2011). Specific recognition of linear ubiquitin chains by the Npl4 zinc finger (NZF) domain of the HOIL-1L subunit of the linear ubiquitin chain assembly complex. Proc. Natl. Acad. Sci. USA *108*, 20520–20525.

Stieglitz, B., Morris Davies, A.C., Koliopoulos, M.G., Christodoulou, E., and Rittinger, K. (2012). LUBAC synthesizes linear ubiquitin chains via a thioester intermediate. EMBO Rep. *13*, 840–846.

Vijay-Kumar, S., Bugg, C.E., and Cook, W.J. (1987). Structure of ubiquitin refined at 1.8 A resolution. J. Mol. Biol. *194*, 531–544.
